# Concomitant deletion of HRAS and NRAS leads to pulmonary immaturity, respiratory failure and neonatal death in mice

**DOI:** 10.1038/s41419-019-2075-2

**Published:** 2019-11-04

**Authors:** Rocío Fuentes-Mateos, David Jimeno, Carmela Gómez, Nuria Calzada, Alberto Fernández-Medarde, Eugenio Santos

**Affiliations:** Centro de Investigación del Cáncer-Instituto de Biología Molecular y Celular del Cáncer (CSIC- Universidad de Salamanca) and CIBERONC, 37007 Salamanca, Spain

**Keywords:** Differentiation, Organogenesis

## Abstract

We reported previously that adult (*HRAS*^*−/−*^; *NRAS*^*−/−*^) double knockout (DKO) mice showed no obvious external phenotype although lower-than-expected numbers of weaned DKO animals were consistently tallied after crossing NRAS-KO and HRAS-KO mice kept on mixed genetic backgrounds. Using mouse strains kept on pure C57Bl/6 background, here we performed an extensive analysis of the offspring from crosses between HRAS-KO and NRAS-KO mice and uncovered the occurrence of very high rates of perinatal mortality of the resulting DKO littermates due to respiratory failure during the first postnatal 24–48 h. The lungs of newborn DKO mice showed normal organ structure and branching but displayed marked defects of maturation including much-reduced alveolar space with thick separating septa and significant alterations of differentiation of alveolar (AT1, AT2 pneumocytes) and bronchiolar (ciliated, Clara cells) cell lineages. We also observed the retention of significantly increased numbers of undifferentiated progenitor precursor cells in distal lung epithelia and the presence of substantial accumulations of periodic acid-Schiff-positive (PAS+) material and ceramide in the lung airways of newborn DKO mice. Interestingly, antenatal dexamethasone treatment partially mitigated the defective lung maturation phenotypes and extended the lifespan of the DKO animals up to 6 days, but was not sufficient to abrogate lethality in these mice. RNA microarray hybridization analyses of the lungs of dexamethasone-treated and untreated mice uncovered transcriptional changes pointing to functional and metabolic alterations that may be mechanistically relevant for the defective lung phenotypes observed in DKO mice. Our data suggest that delayed alveolar differentiation, altered sphingolipid metabolism and ceramide accumulation are primary contributors to the respiratory stress and neonatal lethality shown by DKO mice and uncover specific, critical roles of HRAS and NRAS for correct lung differentiation that are essential for neonatal survival and cannot be substituted by the remaining KRAS function in this organ.

## Introduction

RAS GTPases play critical roles in control of cellular proliferation, differentiation or death^1–3^ acting as biochemical switches shifting between inactive (RAS-GDP) and active (RAS-GTP) conformations in a cycle modulated by negative (GAPs, GTPase activating proteins) and positive (RAS-GEFs, guanine nucleotide exchange factors) regulators^4–7^. Activating point mutations trigger different tumor types (somatic mutations) or inherited developmental syndromes (germline mutations)^2,5,8^. Although the canonical RAS genes are ubiquitous in mammals, they exhibit different expression levels depending on cell type, tissue or developmental stage under study^2,9^.

Prior reports support the functional specificity of different Ras isoforms under various physiological and pathological contexts by demonstrating preferential association of specific RAS isoforms with different tumor types, intracellular processing pathways, subcellular locations or functional interactions with regulators and effectors^2,5,8,10,11^. Analysis of genetically modified mouse strains also supports the functional specificity of the RAS isoforms. Among RAS family members, only KRAS is essential for mouse development and viability whereas HRAS and NRAS are dispensable^12–18^. Transcriptomic analyses have identified specific transcriptional programs controlled by each RAS isoform^2^ and suggested preferential involvement of HRAS with cell growth and proliferation, NRAS with immunomodulatory and apoptotic responses^19,20^, and KRAS with control of cell cycle progression^21,22^. Our earlier studies showed that HRAS/NRAS-DKO mice (expressing only KRAS) were viable and presented no obvious phenotypes, but significantly lower-than-expected numbers of adult DKO animals were obtained when breeding NRAS-KO (HRAS^+/−^;NRAS^−/−^) and HRAS-KO (HRAS^−/−^;NRAS^+/−^) mice kept on mixed genetic background^12^.

To get mechanistic clues for these observations and to ascertain possible differential roles of RAS isoforms in control of tissue/organ development during embryonic or adult stages, here we carried out an extensive breeding program between mice kept on pure C57Bl/6 background to generate single- or double-KO offspring for HRAS and NRAS that were then studied at different embryonic or adult stages by means of immunochemical or transcriptomic analyses. Most DKO offspring died immediately after birth while showing significant respiratory distress and marked signs of pulmonary immaturity and defective differentiation of specialized lung cell types. The lungs of these DKO mice showed also significant transcriptional alterations of components of sphingolipid metabolic pathways that correlated with abnormal accumulations of ceramide, a common feature of various lung diseases in humans^23,24^. These findings indicate that HRAS and NRAS play specific functions during lung maturation that are critical for neonatal survival and cannot be provided by the remaining KRAS isoform in this organ.

## Results

### Simultaneous loss of HRAS and NRAS leads to neonatal death in mice

**Fig. 1:**
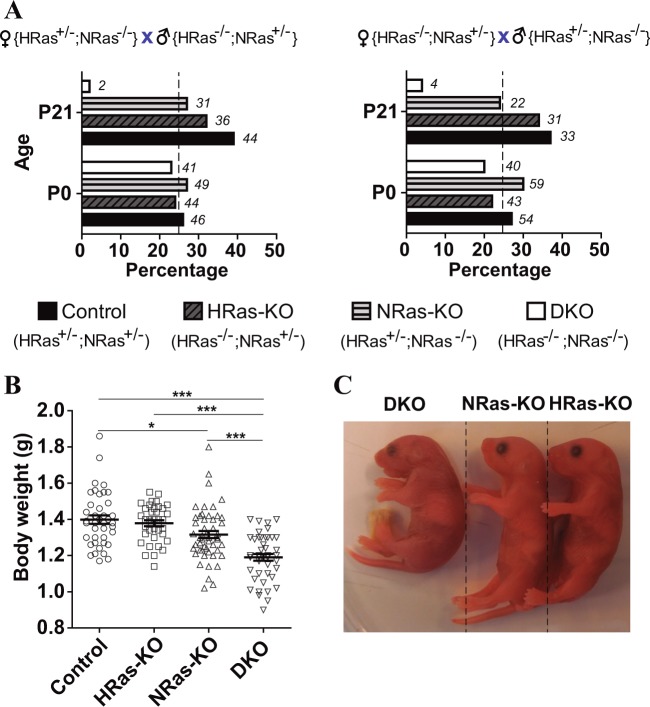
Analysis of the offspring from crosses between HRAS-KO and/or NRAS-KO mice. **a** Bar graphs depicting percentage (%) and absolute numbers (in italics) of individuals of the indicated genotypes (Control*;* HRAS-KO*;* NRAS-KO; DKO) counted at time of birth (P0) or at weaning time (P21) in the litters resulting from parental crosses between heterozygous HRAS-KO and NRAS-KO mice of the indicated sex. **b** Body weight distribution of living, newborn P0 mice of the indicated genotypes at time of birth. Data represented as the mean ± s.e.m. for each genotype. Control, *n* = 41; HRAS-KO, *n* = 39; NRAS-KO, *n* = 52; DKO, *n* = 45. *p < 0.05, ***p < 0.001. **c** Representative picture of a cyanotic DKO pup (left) next to healthy HRAS-KO and NRAS-KO littermates, immediately after birth (P0). See also respiratory distress in Supplementary video 1

We reported previously that significantly less-than-expected adult HRAS/NRAS-DKO mice resulted from crosses between single HRAS-KO and NRAS-KO mice kept on mixed (129/Bl/6) genetic background^12^. To identify possible causes for that observation, here we crossed heterozygous HRAS-KO and NRAS-KO strains kept on pure C57Bl/6 background and counted the numbers of Control, HRAS-KO, NRAS-KO and DKO offspring mice at different developmental stages including E18.5 embryos, P0 neonates or P21 weaned pups (Fig. 1a).

The number of alive DKO E18.5 embryos (not shown) and newborn P0 pups followed expected mendelian rates (~25%) and these frequencies were not sexually biased, as similar percentages were measured independently of the sex of the parental breeding partners (Fig. 1a). In contrast, the percentage of surviving DKO mice pups counted after weaning (P21) was 5–6 fold lower than around birth time (Fig. 1a), and we observed that concomitant HRAS and NRAS loss caused significant neonatal lethality within the first 1-2 postnatal days. The DKO neonates exhibited also significant reduction of body weight (15–20%) and size in comparison to their Control or single HRAS-KO and NRAS-KO littermates (Fig. 1b, c). Notice that single NRAS-KO animals exhibit also a slight reduction of body weight as compared to the Control group (Fig. 1b).

### HRAS/NRAS-DKO mice exhibit impaired lung maturation

**Fig. 2:**
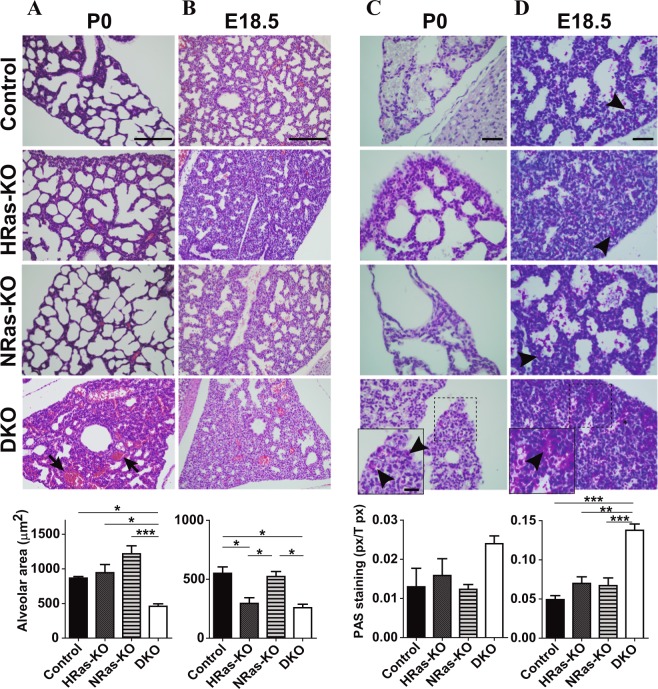
Histological analysis of the lungs of newborn pups (P0) and late embryos (E18.5) of HRAS-KO and/or NRAS-KO mice. **a**, **b**. Representative images of Hematoxylin-Eosin (H&E)-stained sections of lungs from P0 newborn pups (panel **a**) or from E18.5 embryos (panel **b**) of the indicated genotypes. Arrows indicate hemorrhagic regions. Scale bars: 100 µm. The bar graphs at bottom of the panels quantify the average area (µm^2^) of the individual alveolar sacs in the lungs of, respectively, P0 (panel **a**) and E18.5 individuals (panel **b**) of the indicated genotypes. Data are expressed as the mean ± s.e.m. *n* = 3 individuals for each genotype. *p < 0.05, ***p < 0.001. **c**, **d** Representative images of PAS-stained lung sections from P0 newborn mice (panel **c**) or from E18.5 embryos (panel **d**) of the indicated genotypes. Scale bars: 20 µm and 10 µm in magnified, boxed areas. Black arrowheads point to cytoplasmic and extracellular accumulations of PAS-positive label in alveolar areas of the indicated genotypes. The bar graphs in these panels quantify the relative levels of PAS-staining (ratio of PAS+ pixels relative to total number of pixels) in the lungs of P0 (panel **c**) and E18.5 (panel **d**) individuals of the indicated genotypes. Data are expressed as the mean ± s.e.m. *n* = 3 individuals for all genotypes in E18.5 lungs; *n* = 4 for DKO and *n* = 3 for rest of genotypes of P0 lungs. **p < 0.01, ***p < 0.001

In contrast to the other genotypes, most DKO neonates were cyanotic and showed severe respiratory distress, displaying noticeable breathing efforts (Fig. 1c; Supplementary movie 1). Given the recognized connection between impaired respiratory activity and neonatal mortality^25–28^, we examined overall lung morphology and structure in the newborn pups. No morphological or branching differences were found between the lungs of DKO, HRAS-KO, NRAS-KO, and Control mice. However, the lungs of most P0 DKO neonates showed extensive atelectasis and occasional hemorrhages probably related to their early postnatal death (Fig. 2a, arrows). Interestingly, the lungs of the small number of DKO mice that survived to adulthood displayed much smaller patches of atelectasis affecting only limited areas of the structure of otherwise normal organs (Supplementary Fig. S1).

Hematoxylin-Eosin-staining (H&E) revealed that, in contrast to all other genotypes, the lungs of DKO neonates showed also very significant reduction of the alveolar saccular space with notably thicker separating septa (Fig. 2a). The reduced saccular space was already visible at earlier embryonal stages (E18.5) not only in DKO but also in HRAS-KO animals (Fig. 2b). Notice that this defect had disappeared at birth time in HRAS-KO but not in DKO lungs (Fig. 2a, b). PAS-staining of lung sections revealed also significant polysaccharide accumulations in alveolar areas of the lungs of E18.5 DKO mice that were not seen in the three other genotypes (Fig. 2d) and a similar tendency for PAS+ accumulations, though not statistically significant, was also seen in the P0 DKO lungs (Fig. 2c).

### Defective/delayed differentiation of alveolar cell lineages in HRAS/NRAS-DKO mice

**Fig. 3:**
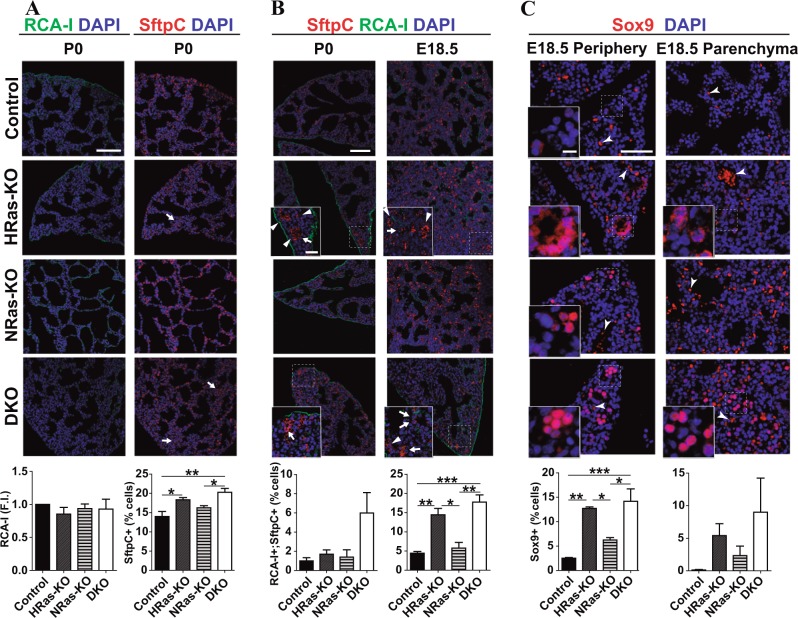
Immunostaining of alveolar differentiation markers in the lungs of HRAS-KO and/or NRAS-KO mice. **a** Representative images of immunostaining for *Ricinus communis* agglutinin-I (RCA-I, AT1 lineage, green) and Surfactant protein-C (SftpC, AT2 lineage, red) in paraffin sections of the lungs of newborn P0 mice of the indicated genotypes. Regions of SftpC+ cell accumulations are marked by tailed arrows. Scale bar: 75 µm. The bottom bar graphs quantify, respectively, the average fluorescence intensity (F.I.) of the RCA-I immunoassay signals (relative to Control animals), and the percentage of SftpC+ pneumocytes (relative to total nuclei) in the lungs of P0 individuals of the indicated genotypes. Data expressed as the mean ± s.e.m. Ten separate microscopic fields were quantified for each individual analyzed in each genotype, *n* = 3 for all genotypes in RCA-I quantification, and *n* = 3 for Control and *n* = 4 for the rest of genotypes in SftpC quantification. *p < 0.05, **p < 0.01. **b** Representative images of immunostaining for Surfactant protein-C (SftpC, AT2 lineage, red) and *Ricinus communis* agglutinin-I (RCA-I, AT1 lineage, green) in paraffin sections of the lungs of newborn P0 and E18.5 mice of the indicated genotypes. Distal-tip like alveolar structures are marked by arrow heads. Co-immunolabeled, alveolar bi-potent progenitor cells are marked by tailed arrows. Scale bar: 75 µm and 25 µm in the magnified boxed areas. The bottom bar graphs quantify the percentage of alveolar bi-potent cells (RCA-I+/SftpC+) relative to total number of SftpC+ cells in the lungs of P0 or E18.5 individuals of the indicated genotypes. Data expressed as the mean ± s.e.m. Ten separate microscopic fields were quantified for each individual analyzed in each genotype. *n* = 3 for all genotypes of E18.5 lungs, *n* = 3 for Controls and *n* = 4 for the rest of genotypes in P0 lungs. *p < 0.05, **p < 0.01, ***p < 0.001. **c** Representative images of immunostainings for Sox9 (distal-tip progenitors, red) in peripheral or inner parenchymal areas of E18.5 lungs from the indicated genotypes. Scale bar: 50 µm and 10 µm in the magnified boxed areas. The bottom bar graphs quantify the percentage of Sox9+ cells relative to total number of cells. Data expressed as the mean ± s.e.m. Non-specific erythrocyte staining indicated by arrow heads. Ten separate microscopic fields were quantified for each of the three individuals analyzed in each genotype. *n* = 3 individuals for all genotypes. *p < 0.05, **p < 0.01, ***p < 0.001

The differentiation of specialized cell types in distal alveolar epithelia, including gas-exchanging AT1 squamous pneumocytes and surfactant-producing AT2 cuboidal pneumocytes, was evaluated using specific markers^29,30^ (Fig. 3).

Consistent with the notion of pulmonary immaturity^31^, the lungs of newborn DKO (and also HRAS-KO) mice exhibited significantly elevated numbers of Surfactant protein C-positive (SftpC+) AT2 cells as compared to normal Controls and NRAS-KO, and these SftpC+ pneumocytes were frequently misplaced in inner parenchymal accumulations instead of being exclusively distributed throughout the luminal surface of the alveolar sacs (Fig. 3a).

Enrichment in PAS+ intracellular content is also a major feature of immature AT2 cells since cytoplasmic glycogen granules are building blocks for surfactant phospholipids^32,33^. Consistent with the SftpC immunoassays, strong increase of PAS+ immunostaining was observed in the lungs of P0 and E18.5 DKO mice as compared to the other genotypes (Fig. 2c, d).

Simultaneous immunoassays against *Ricinus communis* agglutinin-I (RCA-I) (AT1 lineage) and SftpC (AT2 lineage), two markers co-localizing only in the bi-potent alveolar progenitor cells known to differentiate and disappear from normal mouse embryonic lungs before E18.3^29,34,35^, we observed that the lungs of E18.5 and P0 DKO embryos (also the E18.5 HRAS-KO lungs) retained abnormally high numbers of bi-potent alveolar progenitors (originating both AT1 and AT2 lineages) in comparison to normal Controls (Fig. 3b).

The retention of undifferentiated progenitors in alveoli of our KO strains was also monitored with immunoassays of Sex-determining region Y-box 9 (Sox9), a well-established marker of alveolar distal-tip progenitors^36,37^. We detected strong nuclear Sox9 staining in lung distal-tip structures of E18.5 HRAS-KO and DKO mice as compared to Controls. These Sox9+ cells were detected not only in peripheral zones (where distal-tip structures are usually located) but also in inner parenchymal areas of the lungs of HRAS-KO and DKO mice (Fig. 3c). Altogether, these observations point to delayed differentiation of the alveolar cell lineages in DKO mice.

### Alterations of bronchiolar cell lineages in HRAS/NRAS-DKO mice

**Fig. 4:**
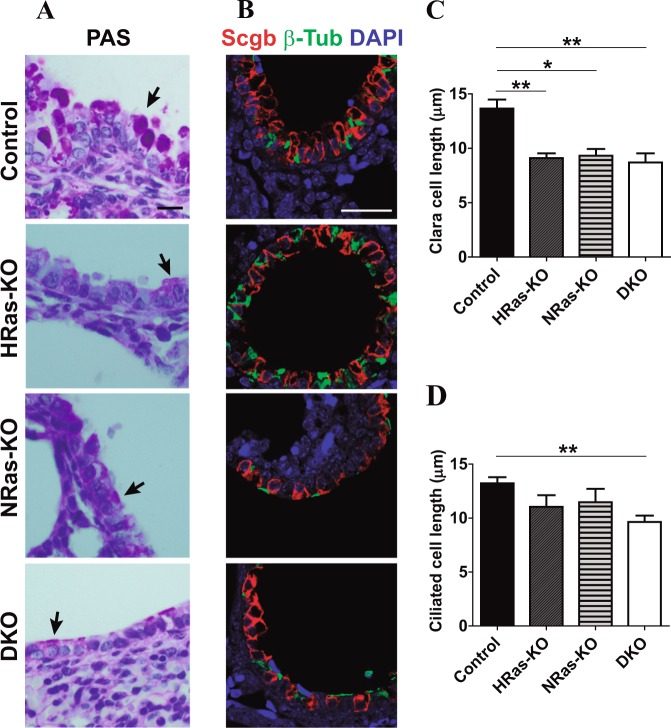
Immunostaining of bronchiolar differentiation markers in the lungs of HRAS-KO and/or NRAS-KO mice. **a** Representative images of PAS-stained lung sections from P0 mice of the indicated genotypes. Tailed arrows point to PAS+ accumulations located in the apical cytoplasmic region of bronchiolar Clara cells. Scale bar: 10 µm. **b** Representative images of immunostaining for Secretoglobulin (Scgb, Clara cells, red) and β-Tubulin (β-Tub, Ciliated cells, green), counterstained with DAPI (blue), in paraffin sections of bronchiolar regions of lungs from P0 mice of the indicated genotypes. Scale bar: 25 µm. **c**, **d** Cell length measurements (from basal to apical membrane) of Clara (panel **c**) and Ciliated (panel **d**) cells stained as in panel (**b**). Data expressed as the mean ± s.e.m. Ten separate microscopy fields were quantified for each individual analyzed in each genotype. *n* = 4 individuals for DKO and *n* = 3 for rest of genotypes. *p < 0.05, **p < 0.01

PAS-staining of lung bronchioles from newborn P0 mice showed that the typical columnar morphology of the PAS+ Clara cells (located in the luminal layer of the bronchioles of normal Control mice) was significantly altered in DKO and single KO littermates (Fig. 4a). These alterations included overall reduction of glycosaminoglycan (PAS) labeling, as well as noticeable morphological flattening linked to shortening of their cytoplasmic, apical vesicular area (Fig. 4a, c).

Immunostaining Clara cells with antibodies to Secretoglobulin (Scgb) and their derived ciliated cells with anti-β-Tubulin (β-Tub) confirmed the columnar-to-cuboidal morphological change occurring in Clara cells of DKO and single KO animals (Fig. 4b, c). Regarding ciliated cells, we only detected statistically significant shortening of this cell type in DKO mice as compared to normal Controls (Fig. 4d).

### Increased rates of proliferation, apoptosis, and infiltrating neutrophils in the lungs of HRAS/NRAS-DKO mice

**Fig. 5:**
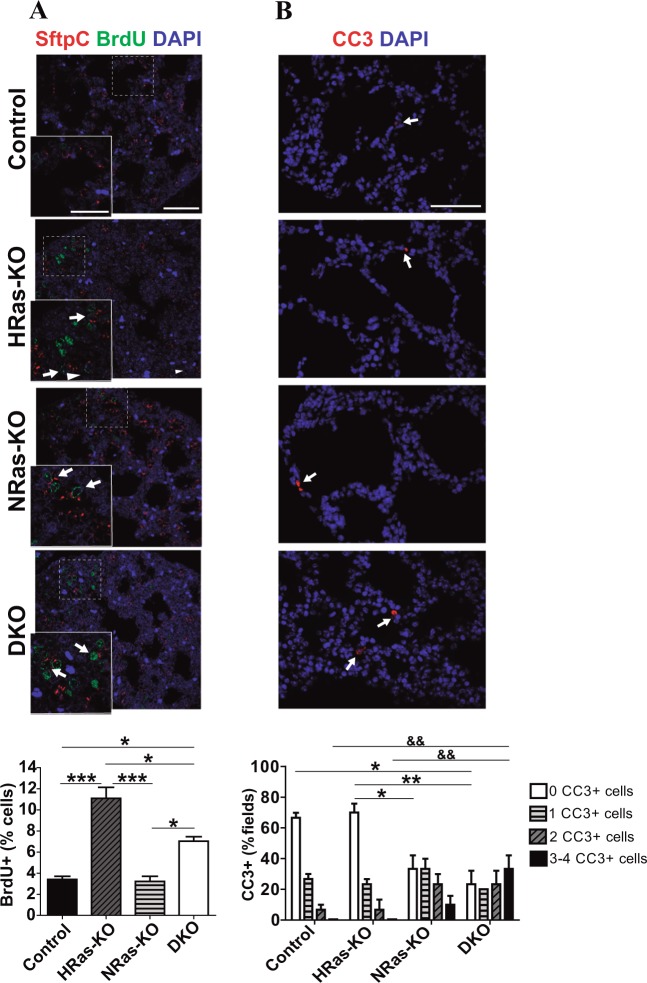
Analysis of proliferative and apoptotic rates in the lungs of HRAS-KO and/or NRAS-KO mice. **a** Representative images of immunostaining for Bromodeoxyridine (BrdU, green) and SftpC (red), counterstained with DAPI (blue), in paraffin sections of E18.5 lungs of the indicated genotypes. Tailed arrows point to cells presenting double staining with BrdU and SftpC. Scale bar: 50 µm and 25 µm for magnified box areas. Bar graphs quantifying percentage of BrdU+ cells relative to total number of cells. Data expressed as mean ± s.e.m. Ten separate microscopy fields were quantified for each individual analyzed. *n* = 3 individuals for all genotypes. *p < 0.05, ***p < 0.001. **b** Representative images of immunostaining for Cleaved caspase-3 (CC3, red) and counterstaining with DAPI (blue) in paraffin sections of P0 lungs of the indicated genotypes. Scale bar: 50 µm. For quantitation of apoptotic (CC3+) cells, the bar plots represent the percentage (%) of microscopy fields containing the specified number of apoptotic cells (0, 1, 2, or 3-4 per individual field) as indicated. Ten separate microscopy fields were quantified for each individual analyzed. *n* = 3 animals for all genotypes. For analysis of statistical significance, the ^*^ and ^&^ characters in the bar plot correspond, respectively, to comparisons between frequencies of samples of each genotype containing 0 apoptotic cells per microscopy field (clear bars) and frequencies of samples of each genotype containing 3–4 apoptotic cells per microscopic field (solid bars). *p < 0.05, **p < 0.01, ^&&^*p* < 0.01

Proliferation and cellular death are also well-balanced processes during normal embryonal and postnatal lung development^29,38–40^. We observed that the number of Bromodeoxyuridine (BrdU+), proliferating cells in lungs of E18.5 embryos was significantly higher in DKO and HRAS-KO mice than in Controls and NRAS-KO littermates. Our immunoassays showed also that a vast majority of these BrdU+ cells at E18.5 corresponded to SftpC+ AT2 cells (Fig. 5a).

Cell death quantitation in lung sections of newborn P0 mice by means of Cleaved-caspase-3 (CC3) immunoassays yielded overall low rates in absolute terms, but we statistically verified significantly higher levels of apoptosis in the lungs of DKO mice than in all other genotypes (Fig. 5b).

Immunoassays for different immune cell types detected significantly higher levels of infiltrating neutrophils in DKO lungs as compared to the other genotypes (Supplementary Fig. S2).

### Specific transcriptomic alterations in the lungs of newborn HRAS/NRAS-DKO mice

**Fig. 6:**
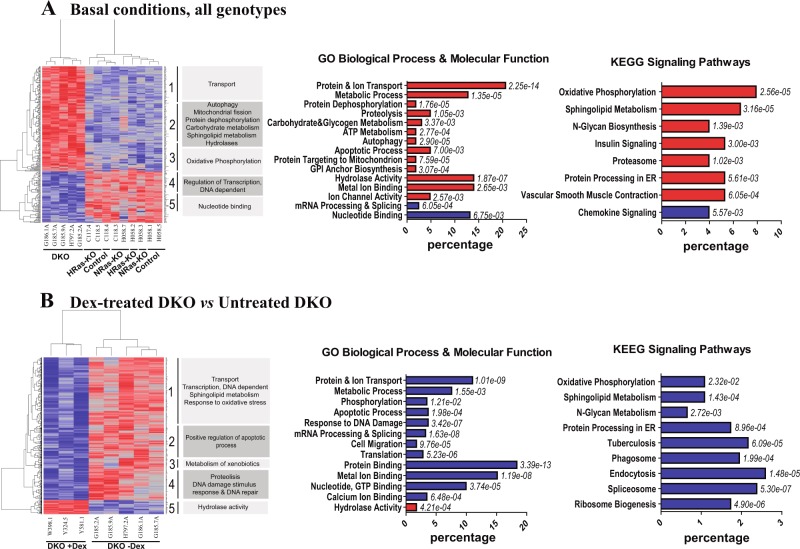
Differential gene expression in the lungs of (A) Untreated and (B) Dexamethasone-treated, HRAS/NRAS-DKO newborn P0 mice. **a** *Lungs under basal conditions*. A set of 14 independent chip microarray hybridizations were performed using RNA extracted from the lungs of at least three independent, newborn P0 mice belonging to each of the four indicated genotypes and analyzed jointly as described in Materials & Methods. The heatmap depicts the results of hierarchical clustering and multiclass comparisons of 265 gene probesets (listed in Table S1) that showed differential expression (FDR = 0.1) in the lungs of DKO mice as compared to the rest of genotypes. Labels on the right side of the dendrogram identify specific functional categories that are enriched at high statistical significance within the indicated, individual horizontal clusters (blocks 1–5). Each individual bar in the horizontal bar plots represents the percentage of the total number of differentially expressed, overexpressed (red bars) or repressed (blue bars), gene probesets corresponding to specific groups of genes of the dendrogram that were identified by GeneCodis as significantly enriched (hypergeometric p-values in italics) for the indicated GO and KEGG functional categories. **b**. *Lungs after antenatal dexamethasone treatment of pregnant mothers*. RNAs extracted from the lungs of three independent, newborn P0 DKO mice that had been previously treated in utero with dexamethasone as described (Materials & Methods) were submitted to microarray hybridizations and their transcriptional profiles were compared to those of five independent, untreated DKO P0 neonates. The heatmap depicts the results of hierarchical clustering and multiclass comparison of 509 gene probesets (listed in Table S3) that showed differential expression (FDR = 0.15) between the untreated and the dexamethasone-treated lung DKO samples. Labels on the right side of the dendrograms identify specific functional categories that are enriched at high statistical significance within the indicated individual horizontal clusters (blocks 1–5) of the heatmap. The horizontal bar plots depict color-coded functional annotations (hypergeometric *p*-values indicated in italics) corresponding to specific groups of genes that are overexpressed (red) or repressed (blue) in dexamethasone-treated DKO lung samples as compared to untreated DKO counterparts and were identified by GeneCodis as significantly enriched for the indicated functional categories. Values in the *X*-axis represent the percentage of the total number of differentially expressed gene probesets corresponding to each individual functional category identified in the graphs

To search for mechanistic clues to the phenotypic defects of lung maturation exhibited by DKO mice, we compared the transcriptional profiles of lung tissues from P0 neonate littermates of the relevant RAS genotypes (Fig. 6a). Multiclass comparisons of microarray expression data profiles generated with high stringency (FDR = 0.1) produced a dendrogram that clearly discriminated all our independent DKO samples from a separate group encompassing the rest of genotypes (Fig. 6a heatmap), suggesting the existence of a distinct pattern of transcriptional alterations specifically linked to HRAS and NRAS disappearance in the lungs of DKO mice. Most differentially expressed probesets in neonate DKO lungs were overexpressed (~75%) whereas only 25% were repressed, suggesting that transcriptional repression is the predominant consequence of HRAS- and NRAS-driven signals in mouse lung tissues at this early developmental stage (Fig. 6a heatmap; Supplementary Table S1).

Functional annotation of the probesets in the dendrogram identified significant enrichment in components of various biological processes, molecular functions and signaling pathways that may be mechanistically significant for generation of the phenotypic alterations observed in newborn DKO lungs (Fig. 6a GO&KEGG; Supplementary Table S2). The group of genes upregulated in DKO samples showed highly statistically significant enrichment in distinct GO (Gene Ontology) functional categories including **“**Transport and Metabolic Processes” or “Hydrolase activity”. 34 overexpressed genes in DKO lungs were related with various functional subcategories of Transport, including “Protein transport”, “Vesicle-mediated transport”, “Intracellular protein transport”, “Ion transport”, “Proton transport”, or “Transmembrane transport”. A separate group of 21 upregulated genes was specifically concerned with various “Protein Metabolic Processes” (Proteolysis, Ubiquitination and Dephosphorylation), as well as “Carbohydrate and Glycogen Metabolism” (Fig. 6a GO; Supplementary Table S2). Consistent with the GO annotations, the list of genes upregulated in DKO lungs was significantly enriched with components of different KEGG signaling pathways including “Oxidative phosphorylation”, “N-glycan biosynthesis” and, particularly, “Sphingolipid metabolism”, that are functionally significant for the developmental processes of lungs in mice (Fig. 6a KEGG; Supplementary Table S2**)**.

### Metabolic/physiological alterations and increased ceramide levels in the lungs of HRAS/NRAS-DKO mice

A mechanistic link was readily apparent between some transcriptional alterations (Supplementary Table S2) and the phenotypic defects observed in DKO lungs. The severe respiratory distress exhibited by P0 mice (Fig. 1c) was paralleled by transcriptional upregulation of significant numbers of loci involved in “Oxidative phosphorylation” and “ATP metabolism” or “Protein targeting to mitochondria” **(**Supplementary Table S2). The increased apoptotic rate detected in P0 lungs (Fig. 5b) was accompanied by overexpression of various loci coding for regulatory components of “Apoptotic and Autophagic processes” (Supplementary Table S2). The significant increase of PAS staining detected in P0 lungs (Fig. 2c, d) or the morphological flattening of secretory Clara cells (Fig. 4a) correlated with transcriptional upregulation of various loci involved in regulation of “Carbohydrate and glycogen metabolism” as well as “Intracellular vesicle-mediated transport” (Supplementary Table S2).

**Fig. 7:**
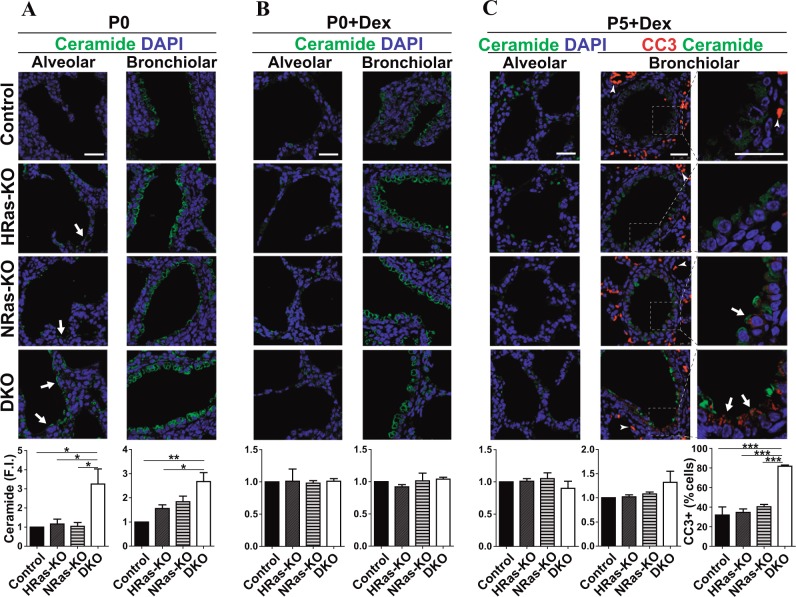
Ceramide immunoassays in alveolar and bronchiolar regions of the lungs of untreated and dexamethasone-treated HRAS and/or NRAS KO mice. **a** Representative images of immunostaining for Ceramide (green) and DAPI counterstaining (blue) in lung paraffin sections of newborn P0 mice of the indicated genotypes under basal, untreated conditions. Alveolar and bronchiolar areas are shown, respectively, in each column of this panel. Arrows point to zones with significantly increased ceramide levels observed in alveolar regions of the indicated genotypes. The bottom bar plots represent the average values of ceramide fluorescence intensity (F.I.) in alveoli and bronchioli (relative to Controls). Scale bar: 25 µm. Data expressed as the mean ± s.e.m. Ten separate microscopy fields were quantified for each individual analyzed. *n* = 3 individuals for Controls and *n* = 4 individuals for the rest of genotypes. *p < 0.05, **p < 0.01. **b** Representative images of immunostaining for Ceramide (green) and DAPI counterstaining (blue) in lung paraffin sections of newborn, dexamethasone-treated mice (P0 + Dex) of the indicated genotypes. Alveolar and bronchiolar areas are shown, respectively, in each column of this panel. The bottom bar plots represent the average values of ceramide fluorescence intensity (F.I.) in alveoli and bronchioles (relative to Controls). Scale bar: 25 µm. Data expressed as the mean ± s.e.m. Ten separate microscopy fields were quantified for each individual analyzed. *n* = 3 individuals for all genotypes. **c** Representative images of immunostaining for Ceramide (green) and DAPI counterstaining (blue) in lung paraffin sections of P5, dexamethasone-treated mice (P5Dex) of the indicated genotypes. First column: alveolar regions immunostained for Ceramide and DAPI. Second and third columns: Bronchiolar regions immunostained for Cleaved Caspase-3 (CC3, red) and Ceramide and counterstained with DAPI. Pictures in the right column contain magnifications of the areas marked by squares in the left column. Tailed arrows point to CC3+ bronchiolar cells. Arrowheads point to non-specific CC3 staining of erythrocytes. The bottom bar plots represent the average values of ceramide fluorescence intensity (F.I.) in alveoli and bronchioles (relative to Controls), and the percentage of CC3+ cells (relative to total nuclei). Scale bar: 25 µm. Data expressed as the mean ± s.e.m. Ten separate microscopy fields were quantified for each individual analyzed. *n* = 3 individuals for Controls and DKO, *n* = 5 individuals for HRAS-KO and *n* = 4 individuals for NRAS-KO. ***p < 0.001

The significant upregulation of genes related to “Sphingolipid metabolism” or “GPI anchor biosynthesis” (Supplementary Table S2) is also likely to be relevant for the defective DKO lung phenotypes in view of the critical roles that ceramides and surfactants play in multiple physiological and pathological lung processes^23,24,41–45^. Of note, our transcriptomic analyses uncovered significant overexpression in DKO lungs of an important number of loci involved in sphingolipid metabolic pathways controlling the levels of cellular ceramide (*Sptlc1*, Serine palmitoyltransferase, long chain base subunit 1; *Cers5*, Ceramide synthase 5; *Degs1*, Delta(4)-desaturase, sphingolipid 1; *Sgpp1*, Sphingosine-1-phosphate phosphatase 1; *Acer3*, alkaline ceramidase 3) (Supplementary Table S2, Supplementary Fig S3). Consistent with the transcriptomic alterations, our parallel immunohistochemical studies of the lungs of P0 mice detected significantly elevated levels of ceramide in lung alveoli of newborn DKO mice as compared to all three other genotypes (Fig. 7a).

### Partial rescue of defective DKO lung phenotypes by antenatal treatment with glucocorticoids

**Fig. 8:**
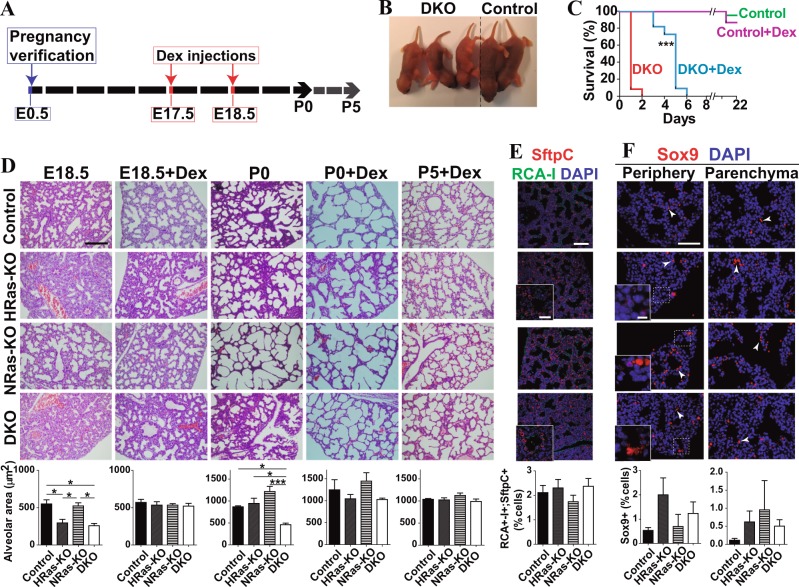
Effect on antenatal dexamethasone treatment on the lungs of HRAS-KO and/or NRAS-KO mice. **a** Schedule of dexamethasone injections of pregnant mothers and timeline for analysis of mouse embryonic development. See Materials and Methods for details of the experimental procedure. **b** Representative pictures of living P4 littermates that were treated antenatally with dexamethasone and subsequently genotyped as double-heterozygous Controls (two animals on the right) or DKO (three animals on the left) individuals. **c** Kaplan–Meier plots comparing the survival rates of untreated and dexamethasone-treated Control and DKO littermates. *n* = 20 for untreated Controls and *n* = 15 for dexamethasone-treated Controls. *n* = 12 for untreated DKO and *n* = 11 for dexamethasone-treated DKO individuals. ***p < 0.001 for comparison between untreated (red) and treated (blue) DKO mice. Dexamethasone treatment extended survival of DKO mice from 1.1 ± 0.09 days up to 4.56 ± 0.34 days. **d** H&E staining of untreated and dexamethasone-treated (+Dex) lungs of mouse embryos (E18.5) and neonates (P0, P5) of the indicate genotypes. Scale bar: 100 µm. Bar graphs in the lower row quantify the average area of the open alveoli observed in each experimental group as indicated. Data expressed as the mean ± s.e.m. for each genotype. E18.5: *n* = 3 for all genotypes. E18.5+Dex: *n* = 4 for the DKO and *n* = 3 for the rest of genotypes. P0: *n* = 3 for all genotypes. P0+Dex: *n* = 2 for HRAS-KO and *n* = 3 for the rest of genotypes. P5+Dex: *n* = 1 for NRAS-KO and *n* = 2 for the rest of genotypes. *p < 0.05, ***p < 0.001. **e** Representative images of immunostaining for RCA-I (green) and SftpC (red), counterstained with DAPI (blue) in lung paraffin sections of E18.5 embryos of the indicated genotypes that had been treated at E17.5 with a single dose of glucocorticoids (E18.5+Dex). Scale bar: 75 µm, and 25 µm in magnified boxed areas. The bottom bar graph quantitates the percentage of alveolar bi-potent cells (RCA-I+/SftpC+) relative to total number of SftpC+ cells. Data expressed as the mean ± s.e.m. for each genotype. *n* = 4 individuals for the DKO and *n* = 3 for the rest of genotypes. **f** Representative images of immunostaining for Sox9 (red) and DAPI (blue) in paraffin sections of peripheral alveolar areas (left column) or inner parenchymal regions (right column) of the lungs of E18.5 embryos that had been treated at E17.5 with a single antenatal dose of dexamethasone (E18.5+Dex). Scale bar: 50 µm, and 10 µm in the magnified boxed areas. The bottom bar plot quantitates the percentage of Sox9+ cells relative to total number of cells in the samples. Non-specific staining of erythrocytes is indicated by arrow heads. Data expressed as the mean ± s.e.m. Ten separate microscopic fields were counted for each individual analyzed. *n* = 3 individuals for all genotypes

To test the possibility of counteracting the defective developmental phenotypes observed in the lungs of newborn DKO mice by means of antenatal administration of glucocorticoids^44–46^, we gave subcutaneous dexamethasone injections to pregnant female mice on days E17.5 and E18.5 of gestation (see Materials and Methods for details), and the lungs of the pups in the resulting litters were subsequently examined at later developmental stages (E18.5, P0, P5) (Fig. 8a).

Antenatal dexamethasone treatment extended the lifespan of newborn DKO mice in comparison to untreated animals of the same genotype. Whereas most untreated DKO pups were routinely dead within the first two postnatal days, dexamethasone treatment caused a delay of 4–5 days in the timing of death for pups of the DKO genotype (Fig. 8c). As with untreated animals, the size of dexamethasone-treated DKO pups was significantly smaller than their similarly treated Control littermates (Fig. 8b).

Histological analysis showed that the glucocorticoid treatment was capable of resolving the alveolar differentiation defects observed in DKO mice. Comparison of H&E-stained, dexamethasone-treated and untreated E18.5 lung samples showed that a single antenatal dexamethasone injection (at E17.5) was enough to correct the altered saccular architecture and reduced alveolar area observed in the lungs of DKO mice. Indeed, the dexamethasone treatment produced a complete reversion to normal values of the reduced alveolar space typically seen in untreated newborn DKO animals (Fig. 8d). Consistently, the practical absence of RCA-I+/SftpC+ cells (bi-potent progenitors) (Fig. 8e), and the significant reduction of Sox9+ cells (alveolar distal-tip progenitors) found in the parenchymal and peripheral lung regions (Fig. 8f) of the dexamethasone-treated, E18.5 embryos of all four relevant genotypes confirmed that a single dexamethasone pretreatment was able to rescue the defective differentiation of pneumocytic lineages observed in the lungs of untreated DKO mice. Dexamethasone treatment also reverted the increase of infiltrating neutrophils specifically observed in P0 DKO lungs (Supplementary Fig. S2).

In contrast, the administration of glucocorticoids could not fully recover other developmental defects in the lungs of DKO mice. We observed that dexamethasone treatment reduced ceramide immunolabeling in the alveoli and bronchioles of P0 and P5 DKO mice to levels that were almost similar to those measured in the rest of genotypes (Fig. 7b, c). However, at P5 (nearing their death time), the lungs of dexamethasone-treated DKO mice showed substantially higher levels of bronchiolar cell apoptosis in comparison to all other genotypes (Fig. 7c).

### Transcriptomic changes induced by antenatal dexamethasone treatment in the lungs of newborn P0 DKO mice

Figure 6b shows a comparison between the transcriptional profiles of lungs from dexamethasone-treated (antenatal injections at E17.5 and E18.5) and untreated, newborn P0 DKO mice. Unsupervised hierarchical clustering of the normalized expression data profiles generated under high stringency produced a dendrogram that clearly discriminated the group of vertical branches corresponding to dexamethasone-treated newborn DKO lungs from the untreated DKO lung samples (Fig. 6b heatmap). More than 90% of differentially expressed genes in this heatmap corresponded to genes downregulated after treatment of the DKO animals with dexamethasone, whereas less than 10% were upregulated as a result of this treatment (Supplementary Table S3, Fig. 6b heatmap).

Functional annotation of the genes differentially expressed in dexamethasone-treated DKO samples identified a series of GO functional categories and KEGG signaling pathways (Fig. 6b **GO**&**KEGG**; Supplementary Table S4) that, for the most part, mirrored, in exactly opposite direction (downregulation), the transcriptional behavior of similar functional categories that were previously found upregulated in the untreated DKO lungs (Fig. 6a **GO**&**KEGG**). Notice for example the significant downregulation observed in functional categories such as “Protein Transport” and “Metabolic”, “Phosphorylation” or “Apoptotic” cellular processes (Fig. 6b **GO**, Supplementary Table S4). It was also striking the significant downregulation of components of *various* signaling pathways that were otherwise upregulated in DKO lungs under basal conditions (Fig. 6a) and are known to be significant for lung functionality, such as “Oxidative phosphorylation”, “N-glycan metabolism” and particularly, “Sphingolipid metabolism” (Fig. 6b **KEGG**; Supplementary Table S4). Indeed, the dexamethasone treatment of pregnant mothers caused in the lungs of the resulting DKO offspring a clear downregulation of several components of sphingolipid metabolic pathways (Supplementary Fig S3) that were previously found specifically upregulated in DKO lungs under basal conditions (Fig. 6a, Supplementary Table S2). Specifically, compared to untreated DKO lungs, the glucocorticoid treatment caused downregulation of loci such Alkaline ceramidase 2 (*Acer2*), Alkaline ceramidase 3 (*Acer3*), Delta(4)-desaturase, sphingolipid 1 (*Degs1*), Neuraminidase 3 (*Neu3*) and Sphingosine kinase 1 (*Sphk1*) (Fig. 6b, Supplementary Table S4).

## Discussion

This report confirms and extend our initial observations on the viability of HRAS/NRAS-DKO mice kept on mixed genetic background^12^ by demonstrating that a majority of DKO mice kept on pure C57Bl/6 background are unable to reach adulthood and die during their first postnatal days due to respiratory failure. Thus, despite the recognized functional dominance of KRAS regarding adult viability and lung tumorigenesis^1,2,47,48^, the HRAS and NRAS family members (undergoing markedly different intracellular processing than KRAS^10^) also exert critical functions regarding fetal lung development and survival of adult mice.

The hard-breathing, cyanotic newborn DKO mice showed unaltered lung morphology and internal branching but also exhibited significant defects of internal maturation/differentiation. We observed significant defects of alveolar development including markedly reduced saccular space and thicker septa, as well as abnormal accumulation of PAS+ material in the alveolar cells. Consistent with defective/delayed differentiation of the alveolar (AT1, AT2) cell lineages, we also detected much-elevated levels of alveolar bi-potent progenitors^29^ and distal-tip progenitors^36,37^ in the lungs of DKO newborns as compared to Control littermates. In addition, the abnormally flattened morphology of Clara and ciliated cells, together with their altered PAS-staining patterns, suggest a secretory deficiency at the bronchiolar level which may also contribute to their respiratory stress of newborn DKO mice since correct mucin production is critical for normal lung function^49^. The small, but reproducible, increase of apoptotic rates in the lungs of DKO mice may have also contributed to the respiratory distress and postnatal death observed in these animals. Consistent with our observations in mice, human lung pathologies such as bronchopulmonary dysplasia (BPD) involve impaired alveolarization, dysregulated vascularization and high apoptosis in the alveolar epithelium^40^. It is unclear whether respiratory disorders seen in Costello-syndrome patients carrying HRAS germline mutations^50^ might be mechanistically related to phenotypes of the DKO mice. Anyhow, no major changes of RAS-downstream-effector-activity were detected (not shown) in our DKO lung samples. Our detection of increased levels of proliferating BrdU+ cells in the lungs of DKO and HRAS-KO mice, and of apoptotic CC3+ cells in the lungs of DKO and NRAS-KO mice, is also consistent with our prior transcriptomic analyses of RAS-KO MEFs indicating a preferential link of HRAS with control of cellular proliferation and of NRAS with control of cell death^19,20,22^. The elevated levels of infiltrating neutrophils detected in the lungs of newborn DKO mice may also contribute to their defective respiratory phenotype since neutrophil presence/activation is a well-established hallmark in acute-respiratory-distress-syndromes (ARDS)^51^.

Transcriptional analysis yielded additional mechanistic clues regarding the defective lung phenotypes of DKO mice. In DKO lungs under basal conditions, we uncovered the overexpression of several distinct groups of genes coding for components of various cellular processes required for correct lung function including “Transport”, “Oxidative phosphorylation”, “Carbohydrate/glycogen metabolism” or “Sphingolipid metabolism”. The overexpression of components of signaling pathways regulating sphingolipid metabolism and ceramide production may be particularly relevant since our immunoassays also confirmed the significant accumulation of ceramides in the lungs of the DKO mice. In the context of mouse RAS genotypes studied here, it is also worth mentioning that sphingomyelin metabolism has been reported as a critical regulator of KRAS function and plasma membrane localization^52,53^. Given the recognized relation of ceramides with stress signals, tissue injury and apoptosis^23,24,41,42,54^, as well as with decreased surfactant production and various lung pathologies including ARDS (acute-respiratory-distress syndrome) and BPD^23,24,41–45^, we postulate that ceramide accumulation is a major factor for the respiratory stress and neonatal death of the DKO mice.

Treatment with glucocorticoids alleviates lung pathologies and improves pneumocytic differentiation in immature fetal lungs *via* upregulation of TTF-1, an essential transcription factor for correct lung morphogenesis and differentiation whose activity is also inhibited by ceramides^44–46^. Antenatal dexamethasone treatment significantly reverted the defects of differentiation of alveolar cell lineages and extended for 5–6 more days the lifespan of newborn DKO mice. Our transcriptional analyses showed also that this treatment reversed many transcriptional alterations observed in lung of untreated newborn DKOs, including several related to ceramide/sphingosine metabolism. However, it was apparent that the antenatal glucocorticoid injections produced only partial rescue of the defective DKO lung phenotypes since the dexamethasone-treated DKO mice still died around P5/P6 due to likely respiratory failure while showing abnormally high apoptotic levels in their bronchiolar cells.

Our observations in HRAS/NRAS-DKO mice indicate that, despite the predominant role commonly attributed to KRAS regarding cell cycle progression, adult viability and lung tumorigenesis^21,22,47,48,55,56^, the HRAS and NRAS isoforms play crucial, specific functions during early lung maturation that are critical for neonatal survival and cannot be substituted by the action of the remaining KRAS isoform in this organ.

## materials and methods

### Animal care, genotyping, and handling

Laboratory mice were managed and handled according to EU and Spanish guidelines for the use and care of animals in research. All NRAS and HRAS knockout strains^12,57^ to be used here were maintained on pure C57Bl/6 background and kept on a 12 h light/dark cycle. Single heterozygous *HRAS*^*+/−*^ or *NRAS*^*+/−*^ mice, as well as double heterozygous (*HRAS*^*+/−*^*;NRAS*^+/−^) mice are phenotypically indistinguishable from wild-type animals. Thus, we routinely set out parental crosses between mouse strains that were homozygous null-mutant for one of the *HRAS* or *NRAS* genes and heterozygous for the other one (*♀/♂ HRAS*^*+/−*^*;NRAS*^*−/−*^ ***×*** *♂/♀ HRAS*^*−/−*^*;NRAS*^*+/−*^) in order to more quickly and efficiently generate comparable sets of littermates of 4 relevant genotypes of interest for our studies (*HRAS*^*+/−*^*;NRAS*^*+/−*^, designated hereafter as Control; *HRAS*^*−/−*^*;NRAS*^*+/−*^ designated as HRAS-KO; *HRAS*^*+/−*^*;NRAS*^*−/−*^ designated NRAS-KO; and *HRAS*^*−/−*^*;NRAS*^*−/−*^ designated as DKO).

Genotyping was done by PCR analysis of genomic DNA isolated from mouse tails using specific primers for the wild-type (WT) or the null-mutant alleles of *HRAS* or *NRAS*, as appropriate. Primers used were as follows. *HRAS* WT allele: (Forward 5′-AGCTCCCTGGCCCCTTGTGG-3′ and reverse 5′-ACCTGCCAATGAGAAGCACACTTAGCC-3′) generating a specific 434 bp fragment. *HRAS* null-mutant allele: (Forward 5′-AGCTCCCTGGCCCCTTGTGG-3′ and reverse 5′-CTACCGGTGGATGTGGAATGTGTGCGA-3′) generating a specific 336 bp fragment. *NRAS* WT allele: (Forward 5′-CCAGGATTCTTACCGAAAGCAAGTGGTG-3′ and reverse 5′-GATGGCAAATACACAGAGGAACCCTTCG-3′) generating a specific 185 bp fragment. *NRAS* null-mutant allele: (Forward 5′-CCAGGATTCTTACCGAAAGCAAGTGGTG-3′ and reverse 5′-CATATGCGGTGTGAAATACCGCACAGATGC-3′) generating a specific 315 bp fragment.

For dexamethasone treatment of pregnant females, the beginning of gestation (E0.5) was timed *via* the detection of vaginal plugs, and pregnancy was later confirmed by weighting the females from day 10 *post coitum* (pc). Dexamethasone (SIGMA, D2915) or saline control (NaCl 0.9%) was injected subcutaneously (0.4 mg/kg) to pregnant females on days E17.5 and E18.5 of embryonic development, and survival of the newborn pups was monitored daily. Thus, embryos collected at E18.5 received only one dose of dexamethasone at E17.5^58,59^.

### Histology and immunohistochemistry

Mouse lung tissues were fixed in 4% paraformaldehyde overnight at 4 °C for 3 days before dehydration and paraffin embedding. Three-micrometer sections were used for tissue staining with Hematoxylin-Eosin (H&E) and five-micrometer sections for periodic acid-Schiff (PAS) according to standard procedures.

Immunohistochemical procedures were performed as previously described using deparaffinized and rehydrated, three-micrometer-thick sections^60^. Antigen retrieval was routinely performed to facilitate antibody binding to antigen using citrate buffer 0.01 M pH 6.0 and heating in a microwave oven (3 × 3 min each, 250 W).

Detection of neutrophils in lung sections was carried out using an avidin-biotin-peroxidase procedure^61^. Sections were rinsed in PBS (3 × 10 min) and endogenous peroxidase activity was blocked with 0.03% hydrogen peroxide for 15 min. Sections were sequentially incubated in (1) primary antibody Neutrophil elastase (NE) (1:400, Abcam, ab68672) in PBS, 0.1% Tween20, 2% BSA and 2% goat serum, overnight at 4 °C; (2) 1:250 biotinylated goat anti rabbit IgG (Vector); and (3) 1:250 Vectastain Elite ABC reagent (Vector) in PBS for 1 h at room temperature. The sections were rinsed in PBS (3 × 10 min) between each step. The reaction product was visualized by incubating the sections in 0.05% 3,3′-diaminobenzidine and 0.0033% hydrogen peroxide in PBS until the desired staining intensity was reached.

For immunofluorescence, sections were washed with PBS and blocked in PBS containing 0.2% Tween20 (Sigma Aldrich, 9005-64-5), 5% Bovine serum albumin (BSA) (Sigma Aldrich, 9048-46-8) and 2% goat serum (Sigma, G9023). Primary antibodies were diluted in PBS, 0.1% Tween20, 2% BSA and 2% goat serum, and incubated with the sections overnight at 4 °C. Primary antibodies (dilution and origin) used in this study included: β-Tubulin (β-Tub) (1:500, Sigma, T5293), Ceramide (1:100, Enzo, ALX-804-196), Cleaved-caspase-3 (CC3) (1:400, Cell Signaling, 9661), *Ricinus communis* agglutinin-I (RCA-I) (1:1000, Atom, FL-1081), Uteroglobin (Scgb) (1:1000, Abcam, ab40873), prosurfactant protein-C (SftpC) (1:500, Merck Millipore, AB3786), Sex-determining region Y-box 9 (Sox9) (1:500, Cell Signaling, 82630S). After 3 PBS washes, sections were incubated with secondary antibodies (from Jackson ImmunoResearch; diluted 1:500) including, as appropriate, goat anti-mouse Alexa 488 or Cy3, goat anti-rabbit Alexa 488 or Cy3, and counterstained with nuclear DAPI (Sigma) for 1 h at room temperature (RT), washed with PBS and mounted with ProLong Diamond anti-fading reagent (P36970, Life Technologies).

Images were acquired using a Leica TCS SP5 confocal microscope with the pinhole set to 1 Airy units and 40 × 1.25NA or 63 × 1.40NA immersion oil objectives. The proper laser lines, 405 nm, 488 nm and 561 were employed to excite Hoechst 33342, Alexa 488/FITC and Cy3, respectively. Images were acquired sequentially, starting first with Hoechst 33342 and following with the Cy3 and Alexa 488 staining. Images were imported to ImageJ software (NIH, Bethesda, MD, USA) using the LOCI Bio-formats plug-in and Adobe Photoshop CS6 version 13.0 for minor adjustments of brightness and contrast.

### BrdU incorporation

Cellular proliferation was measured by nuclear incorporation of BrdU in the lungs of E18.5 embryos. BrdU (0.1 mg/g body mass) (B5002, Sigma Aldrich) was injected intraperitoneally into pregnant female mice at E18.5 and 2 h later the animals were anesthetized with isoflurane prior to euthanasia by cervical dislocation and removal of the embryos. Each embryo was weighted and processed for paraffin sections. Three-micrometer sections were deparaffinized and rehydrated, submitted to antigen retrieval treatment as described above, treated with 2N HCl for 45 min at 37 °C, neutralized with borate buffer (0.1 M pH 8.5 three times for 10 min each), washed with PBS and blocked with PBS, 0.2% Tween20, 5% BSA and 2% goat serum. After PBS washing, sections were immunostained overnight at 4 °C with a primary antibody for BrdU (1:2000, Accurate Chemical, OBT0030CX, diluted in PBS, 0.1% Tween20, 2% BSA and 2% goat serum). Sections were washed with PBS and incubated with the secondary antibody (1:500 diluted, goat anti-rat Alexa 488 from Jackson ImmunoResearch) and counterstained with nuclear marker DAPI (Sigma) for 1 h at RT. Preparations were then washed with PBS and mounted with ProLong Diamond anti-fading reagent (P36970, Life Technologies).

### Image analysis and quantifications

For BrdU+, SftpC+, SfptC+/RCA-I+ and NE+ cell density analyses, images from E18.5 or P0 lung sections of the four genotypes were obtained as described above. Equivalent lung sections from the four genotypes (Control, HRAS-KO, NRAS-KO and HRAS/NRAS-DKO) were selected for the analysis. BrdU-positive nuclei, SftpC+, SftpC+/RCA-I+or NE+ cells, and total nuclei, were counted using the command “Cell Counter” of the ImageJ software and were relativized to the total number of nuclei depending on the region. Cell density data are represented as percentage of BrdU+, SftpC+, SftpC+/RCA-I+or NE+cells.

For Ceramide and RCA-I quantification, digital images taken from equivalent alveolar or bronchiolar lung sections of control and KO animals were treated to balance the signal-to-noise ratio in such a way that the positive element (Ceramide or RCA-I) was clearly distinguishable from the background. The surface analyzed was then delimited using the original image as reference, and both the average fluorescent intensity and total number of nuclei were measured in the chosen area using the ImageJ software (NIH).

For PAS+ quantification, digital images were taken from equivalent lung sections of Control and KO animals. They were manually transformed into binary images in which only PAS positive staining elements appeared as black pixels. Then, the surface analyzed was delimited, using the original image as reference, and average PAS staining was measured as the black/white pixel ratio in the chosen area.

For alveolar area quantification, images taken from equivalent lung sections of Control and KO animals were manually transformed into binary images with the ImageJ software (NIH) where the alveolar spaces were recognized as positive element (Black). The alveolar surface analyzed was then delimited using the original image as reference, and the area (µm^2^) of each alveolus was calculated using the ImageJ software (NIH).

For measurements of Ciliate and Clara cell length, equivalent images of bronchiolar areas of Control and KO animals were obtained and the length of each cell type was manually measured using the ImageJ software (NIH).

### Microarray hybridizations

Lungs were dissected from P0 neonate mice and RNA was extracted using Trizol following the manufacturer’s instructions. After the extraction, the RNA was further purified using RNAse Mini Kit columns (QIAGEN, 74104). RNA quantification and quality was checked by RNA capillary electrophoresis columns (Agilent Technologies, RNA 6000 Nanochips).

Chip microarray hybridizations and data generated with Affymetrix GeneChip Mouse Gene 2.0 ST Array (26,515 genes) were used in this study. All microarray hybridization data were deposited and are available at the NCBI Gene Expression Omnibus (GEO) database (https://www.ncbi.nlm.nih.gov/geo/query/acc.cgi?acc=GSE130415). The RNAs were pre-amplified prior to microarray hybridization using the Gene Chip Expression 3’-Amplification Two-Cycle cDNA Synthesis kit (Affymetrix, Santa Clara, CA, USA; #900432), the Gene Chip Sample Cleanup Module (Affymetrix #900371) and the MEGAscript T7 High Yield Transcription Kit (Ambion, Austin, TX, USA; #1334), according to Affymetrix instruction manual #701025 rev. 5. The pre-amplified RNAs were then submitted to the Gene Chip microarray hybridization protocol (Affymetrix Expression Analysis Technical Manual, (http://www.affymetrix.com/.%20And%20www.%20affymetrix.com/support/technical/manual/expression_manual.affx) as previously described^19^. Using Bioconductor^62^ and R^63^ as computational tools, the robust microarray analysis (RMA) algorithm^64^ was applied for background correction and normalization of fluorescent hybridization signals. The significance analysis of microarrays (SAM) algorithm^65^ was used to identify probe sets displaying significant differential expression when comparing the KO samples to their respective controls. This method uses permutations to provide robust statistical inference of the most significant genes and provides P values adjusted to multiple testing using false discovery rate (FDR)^66^. The GeneCodis (Gene Annotation Co-occurrence Discovery) software package (http://genecodis.cnb.csic.es/) was used for functional annotation analysis of differentially expressed gene sets in order to identify specific gene subsets sharing co-occurrent functional annotations linking them, with high statistical significance, to particular Gene Ontology (GO) Biological Process or Molecular Function categories and KEGG Signaling Pathways^67^.

### Statistical analysis

Experiments were performed using at least three independent biological replicates in all cases, with actual experimental *n* = values being specified in each figure legend. Animals were selected randomly. Data are expressed as mean ± standard error of the mean (s.e.m.). Normal distribution of the data was tested using the IBM SPSS Statistics 23 package (SPSS, Chicago, IL, USA) and Kolmogorov-Smirnov test. One-way ANOVA followed by the Bonferroni post-test was used for the comparison of parametric values. Survival analysis was performed by the Kaplan–Meier method and between-group differences in survival were tested using the Log-rank (Mantel-Cox) test (GraphPad Prism 5.03 Software, Inc.). Differences between groups were considered statistically significant when *p* < 0.05.

## Supplementary information

Supplementary video

Supplementary text

Supplementary Table 1

Supplementary Table 2

Supplementary Table 3

Supplementary Table 4

Supplementary Figure 1

Supplementary Figure 2

Supplementary Figure 3

Author contribution
