# Supplementary material for: Concomitant deletion of HRAS and NRAS leads to pulmonary immaturity, respiratory failure and neonatal death in mice
Source: Cell Death Dis. 2019 Nov 4;10(11):838. doi: 10.1038/s41419-019-2075-2 (PMC6828777; doi:10.1038/s41419-019-2075-2)
Supplement: Supplementary file 2 — Supplementary text [file 41419_2019_2075_MOESM2_ESM.docx]

**SUPPLEMENTARY MATERIALS**

**Concomitant deletion of HRAS and NRAS leads to pulmonary immaturity, respiratory failure and neonatal death in mice**

Rocío Fuentes-Mateos, David Jimeno, Carmela Gómez, Nuria Calzada, Alberto Fernández-Medarde and Eugenio Santos

**Supplementary Figure S1. Lungs of surviving, adult HRAS/NRAS-DKO mice show partial atelectasis.**

**A.** Representative images of H&E staining of lung sections from one year-old mice of the indicated genotypes (Control and DKO). Scale bar: 500µm for the pictures in the first column and 50µm for the magnifications in the second and third columns. n=4 individuals for DKO and n=3 for Control.

**B.** Representative images of immunostaining for SftpC (red) and RCA-I (green), counterstained with DAPI, in alveolar areas of lung paraffin sections from adult mice of the indicated genotype. Scale bars: 75µm, and 25µm on the higher magnification of boxed areas. n=4 individuals for DKO and n=3 for Control.

**C.** Representative images of immunostaining for Scgb (red) and β-Tubulin (green) counterstained with DAPI, in bronchiolar areas of lung paraffin sections from adult mice of the indicated genotypes. Scale bar: 25µm. n=4 individuals for DKO and n=3 for Control.

**Supplementary Figure S2. Increased neutrophil infiltration in the lungs of HRAS/NRAS-DKO mice.**

Representative images of immunostaining for neutrophil elastase (NE), counterstained with hematoxilin (blue) in lung paraffin sections of untreated (P0) and dexamethasone-treated (P0+Dex) neonates. Arrows point to NE+ cells. Scale bar: 25µm. The bottom bar graph quantitates percentage of NE+ cells relative to total cells. Data expressed as the mean ± s.e.m. for each genotype. n=4 individuals for Controls and NRAS-KO and n=3 for HRAS-KO and DKO. ***p<0.001.

**Supplementary Figure S3. Components of sphingolipid metabolic pathways that are differentially expressed in the lungs of HRAS/NRAS-DKO mice.**

The enzymatic components of sphingolipid metabolism pathways that showed differential expression in our transcriptomic analyses of P0 lungs extracts are marked in red (genes overexpressed in untreated DKO lungs) or blue (repressed in DKO upon dexamethasone treatment. Bi-colored genes showed opposite pattern of differential expression under these conditions (Untreated vs Dex-treated). Ceramide is indicated in green. Enzymatic reactions driving either *de novo* synthesis (yellow arrow) or sphingolipid transformation events (green arrow) lead to ceramide accumulation in the DKO lungs, a trend that was partially corrected after antenatal dexamethasone treatment. SGPL1- Sphingosine-1-Phosphate Lyase 1, SGPP1- Sphinganine phosphate phosphatase 1, SPHK- Sphinganine kinase, SPTLC1- Serine Palmitoyltransferase Long Chain Base Subunit 1, KDSR- 3-Ketodihydrosphingosine Reductase, CERS- Ceramide Synthase, ACER1,2,3- Alkaline Ceramidase 1 to 3, DEGS- Delta 4-Desaturase, Sphingolipid 1, SCPT- Sphingosine Choline Phosphotransferase, SGMS1-2- Sphingomyelin Synthase 1 and 2, SMPD1-5- Sphingomyelin Phosphodiesterase 1 to 5, CERK- Ceramide kinase, UGT8A- UDP Glycosyltransferase 8a, GALC- Galactosylceramidase, NEU3- Neuraminidase 3.

**Supplementary Video 1. Respiratory distress of HRAS-KO and NRAS-KO animals.**

Breathing difficulties and cyanotic appearance of a newborn DKO mouse next to two normal-breathing NRAS-KO littermates. (.mov).

**SUPPLEMENTARY TABLES.**

**Supplementary Table S1.** Differential gene expression in the lungs of Controls, HRAS-KO, NRAS-KO and HRAS/NRAS-DKO mice.

**Supplementary Table S2.** Functional annotation of differentially expressed genes (overexpressed and downregulated) in the lungs of HRAS/NRAS-DKO mice.

**Supplementary Table S3.** Differential gene expression in the lungs of HRAS/NRAS-DKO mice treated antenatally with dexamethasone.

**Supplementary Table S4.** Functional annotation of differentially expressed genes (downregulated and overexpressed) in the lungs of HRAS/NRAS-DKO mice treated antenatally with dexamethasone.
