# Supplementary material for: Concomitant deletion of HRAS and NRAS leads to pulmonary immaturity, respiratory failure and neonatal death in mice
Source: Cell Death Dis. 2019 Nov 4;10(11):838. doi: 10.1038/s41419-019-2075-2 (PMC6828777; doi:10.1038/s41419-019-2075-2)
Supplement: Supplementary file 3 — Supplementary Table 1 [file 41419_2019_2075_MOESM3_ESM.docx]

**Table S1. Differential gene expression in the lungs of Control, HRAS-KO, NRAS-KO and HRAS/NRAS-DKO mice.**

List of 265 differentially expressed gene probesets (FDR=0.10) identified by means of SAM contrasts in multiclass comparisons (**Fig. 6A** heatmap) between the transcriptional profiles of lungs isolated from newborn (P0) mouse littermates of the four relevant genotypes (Control, single HRAS-KO, NRAS-KO and DKO) that were generated by RNA microarray hybridization assays using GeneChip(R) Mouse Gene 2.0 ST Arrays. The differentially expressed loci are identified by *Affymetrix Probeset ID, Genename symbol* or *Description* and listed according to their degree of overexpression or repression in the lung tissue analyzed. *d-value* is a parameter measuring the statistical distance separating the calculated expression value of each gene probeset from the null hypothesis (no-change). *q-value* is the estimated FDR at the largest p-value for which the probe set would be statistically significant. *R-fold* is a measure of the fold change of a probeset in the collection of microarrays provided by the SAM algorithm. Entries in red denote overexpression. Entries in green indicate transcriptional repression. The data list is organized here from maximal to minimal R-fold values.

| **probeset ID** | **d.value** | **p.value** | **q.value** | **R.fold** | **Genename** | **Description** |
| --- | --- | --- | --- | --- | --- | --- |
| 17379873 | 5,03201259 | 0,001545359 | 0,097524576 | 3,516549163 | 1500012F01Rik | RIKEN cDNA 1500012F01 gene |
| 17250744 | 4,962865131 | 0,001655063 | 0,098013534 | 2,744922707 | Snord65 | small nucleolar RNA, C/D box 65 |
| 17257591 | 6,556047075 | 0,000371871 | 0,073866639 | 2,599529728 | Snord104 | small nucleolar RNA, C/D box 104 |
| 17215370 | 4,778245344 | 0,001986766 | 0,100695956 | 2,393749726 | Atg16l1 | autophagy related 16-like 1 (S. cerevisiae) |
| 17231844 | 4,919809738 | 0,001731942 | 0,099252055 | 2,280357661 | Perp | PERP, TP53 apoptosis effector |
| 17222564 | 8,037727227 | 0,000124821 | 0,054095556 | 2,239693089 | Snord89 | small nucleolar RNA, C/D box 89 |
| 17483385 | 11,74553218 | 2,2891E-05 | 0,027251476 | 2,158247887 | Phkg2 | phosphorylase kinase, gamma 2 (testis) |
| 17487796 | 11,30283159 | 2,67782E-05 | 0,027251476 | 2,123408157 | Rabac1 | Rab acceptor 1 (prenylated) |
| 17527977 | 7,635218467 | 0,000161101 | 0,058717847 | 2,0855348 | Glce | glucuronyl C5-epimerase |
| 17467359 | 6,75995963 | 0,000310108 | 0,070811709 | 2,01808281 | Tacstd2 | tumor-associated calcium signal transducer 2 |
| 17480880 | 5,563628316 | 0,000907002 | 0,084873601 | 2,015857269 | Pde2a | phosphodiesterase 2A, cGMP-stimulated |
| 17269911 | 5,280733333 | 0,001205449 | 0,094186645 | 1,89151228 | Vat1 | vesicle amine transport protein 1 homolog (T californica) |
| 17384619 | 4,828541946 | 0,001886132 | 0,100511427 | 1,882488174 | Snord90 | small nucleolar RNA, C/D box 90 |
| 17359008 | 5,506129216 | 0,000958831 | 0,086244922 | 1,849381554 | March5 | membrane-associated ring finger (C3HC4) 5 |
| 17280552 | 6,068434304 | 0,000585664 | 0,080976625 | 1,849277813 | Bcap29 | B cell receptor associated protein 29 |
| 17497769 | 6,2041935 | 0,00052131 | 0,080717083 | 1,848026495 | Rnh1 | ribonuclease/angiogenin inhibitor 1 |
| 17442588 | 4,864905186 | 0,0018153 | 0,10045378 | 1,842105439 | Atp6v0a2 | ATPase, H+ transporting, lysosomal V0 subunit A2 |
| 17214825 | 6,646675023 | 0,000341637 | 0,070811709 | 1,810437366 | Mff | mitochondrial fission factor |
| 17435816 | 5,771473144 | 0,000751516 | 0,081210084 | 1,79977293 | Slc35f6 | solute carrier family 35, member F6 |
| 17364932 | 4,83150626 | 0,001877926 | 0,100511427 | 1,791864034 | Got1 | glutamic-oxaloacetic transaminase 1, soluble |
| 17286962 | 4,933873263 | 0,0017043 | 0,099007076 | 1,790972248 | Mylip | myosin regulatory light chain interacting protein |
| 17224587 | 6,983135268 | 0,000263031 | 0,070811709 | 1,78686799 | Dnpep | aspartyl aminopeptidase |
| 17305856 | 5,937134599 | 0,000654337 | 0,080976625 | 1,731804056 | Exoc5 | exocyst complex component 5 |
| 17222332 | 8,21300871 | 0,000111 | 0,05291631 | 1,7286873 | Mgat4a | mannoside acetylglucosaminyltransferase 4, isoenzyme A |
| 17240357 | 4,830965134 | 0,001879222 | 0,100511427 | 1,727541565 | Gtf3c6 | general transcription factor IIIC, polypeptide 6, alpha |
| 17241032 | 5,077689276 | 0,001470639 | 0,096536895 | 1,70044515 | Ddit4 | DNA-damage-inducible transcript 4 |
| 17247176 | 7,708465148 | 0,000155486 | 0,058518959 | 1,68375335 | Ramp3 | receptor (calcitonin) activity modifying protein 3 |
| 17334948 | 6,805317546 | 0,000301038 | 0,070811709 | 1,673843547 | Atp6v0e | ATPase, H+ transporting, lysosomal V0 subunit E |
| 17497421 | 4,84072141 | 0,001862809 | 0,100511427 | 1,672648335 | Bnip3 | BCL2/adenovirus E1B interacting protein 3 |
| 17365960 | 6,001079009 | 0,000618057 | 0,080976625 | 1,670981714 | Gfra1 | glial cell line derived neurotrophic factor family receptor alpha 1 |
| 17478864 | 6,650632643 | 0,000339046 | 0,070811709 | 1,652730338 | Mtmr10 | myotubularin related protein 10 |
| 17501260 | 5,06138785 | 0,001494826 | 0,096786837 | 1,649433545 | Fbxo8 | F-box protein 8 |
| 17376274 | 6,131592116 | 0,00055068 | 0,080776169 | 1,647494514 | Nop56 | NOP56 ribonucleoprotein |
| **17493556** | **4,963863341** | **0,001652039** | **0,098013534** | **1,646643515** | **Acer3** | **alkaline ceramidase 3** |
| 17353241 | 5,61757118 | 0,000865971 | 0,084494765 | 1,642984978 | Slc25a46 | solute carrier family 25, member 46 |
| 17318587 | 5,039766411 | 0,001530242 | 0,097524576 | 1,64248183 | Slc39a4 | solute carrier family 39 (zinc transporter), member 4 |
| 17430609 | 6,362010068 | 0,000442703 | 0,07674456 | 1,629496894 | Serinc2 | serine incorporator 2 |
| 17458734 | 5,495270584 | 0,000970492 | 0,087015147 | 1,620642067 | Plekha8 | pleckstrin homology domain containing, family A (phosphoinositide binding specific) member 8 |
| 17448960 | 6,019633868 | 0,000607691 | 0,080976625 | 1,613181097 | Clock | circadian locomotor output cycles kaput |
| 17502954 | 6,704605592 | 0,000323929 | 0,070811709 | 1,61077619 | Dnajb1 | DnaJ (Hsp40) homolog, subfamily B, member 1 |
| 17312905 | 7,282825613 | 0,000201268 | 0,062295714 | 1,601363183 | Eif3l | eukaryotic translation initiation factor 3, subunit L |
| 17278110 | 7,941885432 | 0,000134323 | 0,055000696 | 1,591745889 | Ubr7 | ubiquitin protein ligase E3 component n-recognin 7 (putative) |
| 17289824 | 7,396792412 | 0,000187879 | 0,06106808 | 1,591270284 | Mier3 | mesoderm induction early response 1, family member 3 |
| 17333854 | 6,148157149 | 0,000543769 | 0,080717083 | 1,580789994 | Ppp2r1a | protein phosphatase 2, regulatory subunit A, alpha |
| 17231690 | 7,901798417 | 0,000135618 | 0,055000696 | 1,572277731 | Sf3b5 | splicing factor 3b, subunit 5 |
| 17372119 | 6,385378996 | 0,000431474 | 0,07674456 | 1,570025464 | Mtx2 | metaxin 2 |
| 17506137 | 5,27303679 | 0,001212359 | 0,094232839 | 1,559865339 | Wfdc1 | WAP four-disulfide core domain 1 |
| 17413649 | 7,526646028 | 0,000171898 | 0,058717847 | 1,553338702 | Dcaf10 | DDB1 and CUL4 associated factor 10 |
| 17302834 | 4,984539309 | 0,001616191 | 0,097826161 | 1,548654612 | Ubac2 | ubiquitin associated domain containing 2 |
| 17252170 | 4,779530604 | 0,001982879 | 0,100695956 | 1,548560195 | Rnf167 | ring finger protein 167 |
| 17435963 | 5,215850772 | 0,001279305 | 0,095993501 | 1,54745357 | Atraid | all-trans retinoic acid induced differentiation factor |
| 17518585 | 5,577867724 | 0,000896636 | 0,084873601 | 1,546362321 | Spg21 | spastic paraplegia 21 homolog (human) |
| 17402595 | 5,821946496 | 0,000718691 | 0,080976625 | 1,54547311 | Casp6 | caspase 6 |
| 17336190 | 5,864503061 | 0,000696664 | 0,080976625 | 1,544772387 | Ndufa7 | NADH dehydrogenase (ubiquinone) 1 alpha subcomplex, 7 (B14.5a) |
| 17395165 | 5,162998009 | 0,001360935 | 0,096476139 | 1,532801862 | Atp5e | ATP synthase, H+ transporting, mitochondrial F1 complex, epsilon subunit |
| 17267430 | 7,853455205 | 0,000140369 | 0,055000696 | 1,521456865 | Gdpd1 | glycerophosphodiester phosphodiesterase domain containing 1 |
| 17354282 | 6,936819632 | 0,000272533 | 0,070811709 | 1,518011338 | Cdo1 | cysteine dioxygenase 1, cytosolic |
| 17233799 | 5,019197211 | 0,001562203 | 0,097524576 | 1,502612955 | Slc25a16 | solute carrier family 25 (mitochondrial carrier, Graves disease autoantigen), member 16 |
| **17321630** | **5,32109075** | **0,001157939** | **0,093087581** | **1,501758444** | **Cers5** | **ceramide synthase 5** |
| 17440086 | 9,696637687 | 5,09649E-05 | 0,031690702 | 1,50145908 | Rpap2 | RNA polymerase II associated protein 2 |
| 17365384 | 7,587789429 | 0,000167148 | 0,058717847 | 1,499137335 | Actr1a | ARP1 actin-related protein 1A, centractin alpha |
| 17239077 | 5,351524074 | 0,001130297 | 0,092109471 | 1,496818803 | Ginm1 | glycoprotein integral membrane 1 |
| 17249700 | 5,525046255 | 0,000944146 | 0,085732855 | 1,495218821 | Oser1 | oxidative stress responsive serine rich 1 |
| 17420996 | 7,895125885 | 0,000137778 | 0,055000696 | 1,488515612 | Rsg1 | REM2 and RAB-like small GTPase 1 |
| 17447868 | 5,432223498 | 0,001034846 | 0,089000756 | 1,488208034 | Tapt1 | transmembrane anterior posterior transformation 1 |
| 17394036 | 5,194815608 | 0,001305651 | 0,096069042 | 1,484415414 | Fitm2 | fat storage-inducing transmembrane protein 2 |
| 17408135 | 4,791260711 | 0,001959556 | 0,100695956 | 1,483132038 | Gpr89 | G protein-coupled receptor 89 |
| 17215968 | 6,795409484 | 0,00030363 | 0,070811709 | 1,481727438 | Ppp1r7 | protein phosphatase 1, regulatory (inhibitor) subunit 7 |
| 17289037 | 6,395152226 | 0,000425427 | 0,07674456 | 1,47960716 | Ssbp2 | single-stranded DNA binding protein 2 |
| 17222440 | 5,313464363 | 0,001167441 | 0,093276169 | 1,476617017 | Rev1 | REV1 homolog (S. cerevisiae) |
| 17283923 | 6,600197218 | 0,00035805 | 0,072634525 | 1,472674146 | Slc25a29 | solute carrier family 25 (mitochondrial carrier, palmitoylcarnitine transporter), member 29 |
| 17482719 | 5,577253933 | 0,0008975 | 0,084873601 | 1,472282515 | Ubfd1 | ubiquitin family domain containing 1 |
| 17216070 | 5,186843841 | 0,001317313 | 0,096121702 | 1,47221896 | Atg4b | autophagy related 4B, cysteine peptidase |
| 17283445 | 5,921825897 | 0,000664271 | 0,080976625 | 1,468357734 | Lgmn | legumain |
| 17350901 | 5,619933533 | 0,000862948 | 0,084494765 | 1,444393469 | A730017C20Rik | RIKEN cDNA A730017C20 gene |
| 17348957 | 9,093759575 | 6,69454E-05 | 0,03682443 | 1,436272436 | Zfp35 | zinc finger protein 35 |
| 17274184 | 10,55197265 | 3,45525E-05 | 0,027453339 | 1,435293551 | Socs2 | suppressor of cytokine signaling 2 |
| 17315669 | 6,245747322 | 0,000494964 | 0,080648038 | 1,435171048 | AW549877 | expressed sequence AW549877 |
| 17350301 | 5,300771057 | 0,001182126 | 0,093815338 | 1,434075816 | Ythdc2 | YTH domain containing 2 |
| 17317203 | 8,038419602 | 0,000124389 | 0,054095556 | 1,432409572 | Derl1 | Der1-like domain family, member 1 |
| 17503596 | 4,965590085 | 0,001646425 | 0,097907395 | 1,430954978 | Papd5 | PAP associated domain containing 5 |
| 17396315 | 5,76550591 | 0,000756699 | 0,081369219 | 1,428922762 | Tbl1xr1 | transducin (beta)-like 1X-linked receptor 1 |
| 17453617 | 6,302930844 | 0,000469482 | 0,078218073 | 1,427572676 | Tmem120a | transmembrane protein 120A |
| 17538356 | 4,901737045 | 0,001759584 | 0,09985745 | 1,426280856 | Alg13 | asparagine-linked glycosylation 13 |
| 17253972 | 6,087929082 | 0,000574867 | 0,080976625 | 1,422131135 | Psmd11 | proteasome (prosome, macropain) 26S subunit, non-ATPase, 11 |
| 17411732 | 6,860398405 | 0,0002924 | 0,070811709 | 1,416251912 | 2610301B20Rik | RIKEN cDNA 2610301B20 gene |
| 17401768 | 6,304657478 | 0,000468618 | 0,078218073 | 1,415017373 | Clcc1 | chloride channel CLIC-like 1 |
| 17407631 | 5,225371945 | 0,001270235 | 0,095993501 | 1,414158221 | Psmb4 | proteasome (prosome, macropain) subunit, beta type 4 |
| 17339549 | 5,050749029 | 0,001511238 | 0,096786837 | 1,412732315 | Ypel5 | yippee-like 5 (Drosophila) |
| 17341412 | 5,013261207 | 0,001574296 | 0,097524576 | 1,412591472 | Zfp944 | zinc finger protein 944 |
| 17407886 | 5,602844028 | 0,000878928 | 0,084494765 | 1,411260989 | Rprd2 | regulation of nuclear pre-mRNA domain containing 2 |
| 17510836 | 5,097737665 | 0,001445589 | 0,096476139 | 1,410134977 | Usp38 | ubiquitin specific peptidase 38 |
| 17222106 | 5,617646147 | 0,000865539 | 0,084494765 | 1,40863918 | Uggt1 | UDP-glucose glycoprotein glucosyltransferase 1 |
| 17304012 | 5,213521253 | 0,00128276 | 0,095993501 | 1,407416302 | Kcnma1 | potassium large conductance calcium-activated channel, subfamily M, alpha member 1 |
| 17309065 | 5,727172289 | 0,000785205 | 0,082571972 | 1,396818292 | Mzt1 | mitotic spindle organizing protein 1 |
| 17357700 | 5,090808131 | 0,001454227 | 0,096510269 | 1,391853147 | Ms4a4d | membrane-spanning 4-domains, subfamily A, member 4D |
| 17501652 | 6,251705812 | 0,000490213 | 0,080585015 | 1,389690189 | Atp6v1b2 | ATPase, H+ transporting, lysosomal V1 subunit B2 |
| 17527421 | 6,383866807 | 0,000432338 | 0,07674456 | 1,388934252 | Tspan3 | tetraspanin 3 |
| 17281354 | 4,874727219 | 0,001805366 | 0,10045378 | 1,385382471 | Sec23a | SEC23A (S. cerevisiae) |
| 17539797 | 4,844711598 | 0,001855899 | 0,100511427 | 1,382420703 | Clcn5 | chloride channel 5 |
| 17456381 | 5,680159009 | 0,000816734 | 0,083980931 | 1,379463376 | Ptprz1 | protein tyrosine phosphatase, receptor type Z, polypeptide 1 |
| 17474136 | 4,77276715 | 0,001995836 | 0,100695956 | 1,372224394 | Ap2s1 | adaptor-related protein complex 2, sigma 1 subunit |
| 17303765 | 6,412026439 | 0,000420676 | 0,076642027 | 1,371971557 | Mrps16 | mitochondrial ribosomal protein S16 |
| 17450098 | 5,126089454 | 0,001406285 | 0,096476139 | 1,370257017 | Lin54 | lin-54 homolog (C. elegans) |
| 17211498 | 5,973677627 | 0,000635765 | 0,080976625 | 1,369483826 | Lmbrd1 | LMBR1 domain containing 1 |
| 17526929 | 5,514431591 | 0,00095192 | 0,085893442 | 1,366224111 | 1110032A03Rik | RIKEN cDNA 1110032A03 gene |
| 17230153 | 6,165339661 | 0,000535995 | 0,080717083 | 1,363376887 | Fh1 | fumarate hydratase 1 |
| 17277307 | 7,325651301 | 0,000196085 | 0,062295714 | 1,356044836 | Fcf1 | FCF1 small subunit (SSU) processome component homolog (S. cerevisiae) |
| 17327069 | 6,186021727 | 0,000527789 | 0,080717083 | 1,355891044 | Ifnar1 | interferon (alpha and beta) receptor 1 |
| 17254537 | 5,238643359 | 0,001247344 | 0,095652437 | 1,354748371 | Ppm1d | protein phosphatase 1D magnesium-dependent, delta isoform |
| 17275718 | 6,052613541 | 0,000593438 | 0,080976625 | 1,347394723 | Mia2 | melanoma inhibitory activity 2 |
| 17275785 | 5,855418479 | 0,000700551 | 0,080976625 | 1,347178164 | Fam179b | family with sequence similarity 179, member B |
| 17356182 | 5,775078829 | 0,000749356 | 0,081210084 | 1,346406898 | Ppp1ca | protein phosphatase 1, catalytic subunit, alpha isoform |
| 17391598 | 8,43427456 | 9,80426E-05 | 0,048351009 | 1,344438136 | Snrpb | small nuclear ribonucleoprotein B |
| 17436315 | 5,302860093 | 0,001180398 | 0,093815338 | 1,341241645 | Ppp1cb | protein phosphatase 1, catalytic subunit, beta isoform |
| 17491454 | 9,999205914 | 4,31906E-05 | 0,031165779 | 1,33472512 | Nipa1 | non imprinted in Prader-Willi/Angelman syndrome 1 homolog (human) |
| 17514247 | 6,661548942 | 0,000336023 | 0,070811709 | 1,329776715 | Tomm20 | translocase of outer mitochondrial membrane 20 homolog (yeast) |
| 17403706 | 4,845278794 | 0,001854603 | 0,100511427 | 1,327749357 | Pigk | phosphatidylinositol glycan anchor biosynthesis, class K |
| 17381184 | 4,798271406 | 0,001947463 | 0,100695956 | 1,325643318 | Polr3k | polymerase (RNA) III (DNA directed) polypeptide K |
| 17411174 | 5,996663554 | 0,000618921 | 0,080976625 | 1,324076434 | Gipc2 | GIPC PDZ domain containing family, member 2 |
| 17357425 | 9,753677147 | 4,79415E-05 | 0,031165779 | 1,323221633 | Rab3il1 | RAB3A interacting protein (rabin3)-like 1 |
| 17266581 | 4,857703157 | 0,001829121 | 0,100511427 | 1,32102589 | AU040972 | expressed sequence AU040972 |
| 17419222 | 5,196554583 | 0,001301764 | 0,096069042 | 1,320756051 | Snrnp40 | small nuclear ribonucleoprotein 40 (U5) |
| 17469775 | 6,364796035 | 0,000441408 | 0,07674456 | 1,31207617 | Emc3 | ER membrane protein complex subunit 3 |
| 17462905 | 6,715465438 | 0,000322202 | 0,070811709 | 1,310459171 | Lpcat3 | lysophosphatidylcholine acyltransferase 3 |
| **17281971** | **6,088035985** | **0,000574435** | **0,080976625** | **1,310311018** | **Sgpp1** | **sphingosine-1-phosphate phosphatase 1** |
| 17424298 | 7,621291807 | 0,000162828 | 0,058717847 | 1,309983872 | Dctn3 | dynactin 3 |
| 17523531 | 5,619674613 | 0,00086338 | 0,084494765 | 1,307198157 | Tmem42 | transmembrane protein 42 |
| 17538109 | 5,403415416 | 0,001072422 | 0,090018052 | 1,306728387 | Tbc1d8b | TBC1 domain family, member 8B |
| 17448863 | 5,064861306 | 0,001488779 | 0,096786837 | 1,303627173 | Scfd2 | Sec1 family domain containing 2 |
| 17306417 | 5,989552484 | 0,000626263 | 0,080976625 | 1,303250769 | Psmb5 | proteasome (prosome, macropain) subunit, beta type 5 |
| **17230595** | **8,127783839** | **0,000118774** | **0,053926201** | **1,302775315** | **Degs1** | **degenerative spermatocyte homolog 1 (Drosophila)** |
| 17363779 | 5,233653758 | 0,001256846 | 0,095867058 | 1,302734136 | Ak3 | adenylate kinase 3 |
| 17358617 | 4,954762629 | 0,001670611 | 0,098323624 | 1,302392046 | Uhrf2 | ubiquitin-like, containing PHD and RING finger domains 2 |
| 17263877 | 4,786433494 | 0,00197165 | 0,100695956 | 1,301542327 | Prpsap2 | phosphoribosyl pyrophosphate synthetase-associated protein 2 |
| 17464901 | 5,000026846 | 0,001593732 | 0,097826161 | 1,299857411 | Tmem168 | transmembrane protein 168 |
| 17331828 | 7,525310298 | 0,00017233 | 0,058717847 | 1,299808115 | Cldn8 | claudin 8 |
| 17440342 | 5,254826846 | 0,001233523 | 0,095102509 | 1,296142958 | Gm15446 | predicted gene 15446 |
| 17428545 | 5,011168431 | 0,001576888 | 0,097524576 | 1,294827033 | Uqcrh | ubiquinol-cytochrome c reductase hinge protein |
| 17386018 | 5,960355411 | 0,00063922 | 0,080976625 | 1,29076151 | Scn1a | sodium channel, voltage-gated, type I, alpha |
| 17223138 | 4,93193203 | 0,001708619 | 0,099007076 | 1,289370561 | Pgap1 | post-GPI attachment to proteins 1 |
| 17473219 | 9,505388999 | 5,4852E-05 | 0,032686631 | 1,285812037 | Tsen34 | tRNA splicing endonuclease 34 homolog (S. cerevisiae) |
| 17479628 | 4,791233924 | 0,001959988 | 0,100695956 | 1,272475718 | Alpk3 | alpha-kinase 3 |
| 17444236 | 5,202617847 | 0,001295717 | 0,096069042 | 1,265939947 | Wipi2 | WD repeat domain, phosphoinositide interacting 2 |
| 17404570 | 4,847148857 | 0,00184942 | 0,100511427 | 1,264786627 | Slc7a14 | solute carrier family 7 (cationic amino acid transporter, y+ system), member 14 |
| 17364111 | 5,932228566 | 0,000657361 | 0,080976625 | 1,259197164 | Ch25h | cholesterol 25-hydroxylase |
| 17229917 | 4,784711642 | 0,001975105 | 0,100695956 | 1,257935743 | Kcnj9 | potassium inwardly-rectifying channel, subfamily J, member 9 |
| 17249888 | 5,652174648 | 0,00084308 | 0,084494765 | 1,256469624 | Mfap3 | microfibrillar-associated protein 3 |
| 17300802 | 4,972048018 | 0,001637787 | 0,097826161 | 1,255135033 | Parp4 | poly (ADP-ribose) polymerase family, member 4 |
| 17324932 | 5,571447501 | 0,000901387 | 0,084873601 | 1,251749353 | Lrch3 | leucine-rich repeats and calponin homology (CH) domain containing 3 |
| 17526265 | 5,835844021 | 0,000713508 | 0,080976625 | 1,251214202 | Trappc4 | trafficking protein particle complex 4 |
| 17506512 | 5,659977771 | 0,000833578 | 0,084251677 | 1,251059546 | Cpne7 | copine VII |
| 17460809 | 5,050113525 | 0,001512534 | 0,096786837 | 1,246052481 | Zxdc | ZXD family zinc finger C |
| 17343755 | 5,412560456 | 0,001061192 | 0,090018052 | 1,245935857 | H2-Pb | histocompatibility 2, P region beta locus |
| 17236102 | 4,812581737 | 0,001912047 | 0,100695956 | 1,240289903 | Btbd11 | BTB (POZ) domain containing 11 |
| 17431516 | 6,689116524 | 0,000327385 | 0,070811709 | 1,228516901 | Pithd1 | PITH (C-terminal proteasome-interacting domain of thioredoxin-like) domain containing 1 |
| 17505260 | 5,61553232 | 0,000869426 | 0,084494765 | 1,224853356 | Nfat5 | nuclear factor of activated T cells 5 |
| 17399396 | 6,715152031 | 0,000322634 | 0,070811709 | 1,217994932 | Dpm3 | dolichyl-phosphate mannosyltransferase polypeptide 3 |
| 17546082 | 6,236092211 | 0,000501443 | 0,080648038 | 1,213307197 | Gm8817 | predicted gene 8817 |
| 17500523 | 4,720073144 | 0,00211893 | 0,103782082 | 1,209649043 | Saraf | store-operated calcium entry-associated regulatory factor |
| 17501234 | 5,129748451 | 0,00140283 | 0,096476139 | 1,208439667 | Glra3 | glycine receptor, alpha 3 subunit |
| **17292654** | **4,985275004** | **0,001614464** | **0,097826161** | **1,208372101** | **Sptlc1** | **serine palmitoyltransferase, long chain base subunit 1** |
| 17288501 | 5,319069835 | 0,001159235 | 0,093087581 | 1,206700182 | Clptm1l | CLPTM1-like |
| 17425836 | 5,263353037 | 0,001224885 | 0,094948376 | 1,204688654 | Ptbp3 | polypyrimidine tract binding protein 3 |
| 17311512 | 5,124465665 | 0,001408877 | 0,096476139 | 1,204573672 | Mal2 | mal, T cell differentiation protein 2 |
| 17363851 | 5,137227157 | 0,001395056 | 0,096476139 | 1,196000691 | 9930021J03Rik | RIKEN cDNA 9930021J03 gene |
| 17494041 | 5,082145173 | 0,001465456 | 0,096536895 | 1,195518248 | Nup98 | nucleoporin 98 |
| 17345029 | 5,013781884 | 0,001572569 | 0,097524576 | 1,190905972 | Gpr111 | G protein-coupled receptor 111 |
| 17385303 | 4,849992153 | 0,001839918 | 0,100511427 | 1,190084555 | Stam2 | signal transducing adaptor molecule (SH3 domain and ITAM motif) 2 |
| 17451823 | 5,902037852 | 0,000676364 | 0,080976625 | 1,182245636 | Srrm4 | serine/arginine repetitive matrix 4 |
| 17438134 | 6,700459327 | 0,000325657 | 0,070811709 | 1,18106764 | Dcun1d4 | DCN1, defective in cullin neddylation 1, domain containing 4 (S. cerevisiae) |
| 17373120 | 4,933794237 | 0,001704732 | 0,099007076 | 1,180753741 | Celf1 | CUGBP, Elav-like family member 1 |
| 17248975 | 6,183987662 | 0,000528653 | 0,080717083 | 1,1718909 | Olfr1386 | olfactory receptor 1386 |
| 17240235 | 4,966994613 | 0,001643833 | 0,097907395 | 1,165643118 | Rfpl4b | ret finger protein-like 4B |
| 17480127 | 4,985361603 | 0,001613168 | 0,097826161 | 1,155466251 | Ccdc89 | coiled-coil domain containing 89 |
| 17326938 | 5,36541344 | 0,001119068 | 0,091980518 | 1,153007742 | LOC102637192 | keratin-associated protein 20-2-like |
| 17325770 | 4,789429718 | 0,001964307 | 0,100695956 | 1,141566245 | Gm608 | predicted gene 608 |
| 17370350 | 4,900200845 | 0,001761744 | 0,09985745 | 1,100156666 | Olfr350 | olfactory receptor 350 |
| 17357591 | -6,244605593 | 0,000497124 | 0,080648038 | 0,915780274 | A430093F15Rik | RIKEN cDNA A430093F15 gene |
| 17350996 | -7,065093548 | 0,000240571 | 0,068811793 | 0,883285713 | Arsi | arylsulfatase i |
| 17228057 | -6,085778253 | 0,000577026 | 0,080976625 | 0,865764147 | 1700025G04Rik | RIKEN cDNA 1700025G04 gene |
| 17341712 | -5,010288042 | 0,001578615 | 0,097524576 | 0,847553417 | Amdhd2 | amidohydrolase domain containing 2 |
| 17476643 | -7,159416819 | 0,000223727 | 0,06597292 | 0,843392385 | LOC102637163 | major allergen I polypeptide chain 1-like |
| 17319134 | -5,216820319 | 0,001278009 | 0,095993501 | 0,838908108 | Sox10 | SRY (sex determining region Y)-box 10 |
| 17368633 | -5,405514407 | 0,001070694 | 0,090018052 | 0,833814357 | F730016J06Rik | RIKEN cDNA F730016J06 gene |
| 17322923 | -6,514866398 | 0,00038526 | 0,074618174 | 0,833335258 | Gm4262 | predicted gene 4262 |
| 17213645 | -6,647909491 | 0,000340774 | 0,070811709 | 0,826618402 | Fastkd2 | FAST kinase domains 2 |
| 17477774 | -4,977900356 | 0,001628285 | 0,097826161 | 0,826412828 | Hrc | histidine rich calcium binding protein |
| 17396498 | -5,11780908 | 0,001419242 | 0,096476139 | 0,819481846 | Rpl22l1 | ribosomal protein L22 like 1 |
| 17407905 | -6,61293257 | 0,000353731 | 0,072270914 | 0,819389164 | Prpf3 | PRP3 pre-mRNA processing factor 3 homolog (yeast) |
| 17548894 | -5,098484111 | 0,001444725 | 0,096476139 | 0,810831415 | Tmlhe | trimethyllysine hydroxylase, epsilon |
| 17273910 | -5,750294189 | 0,000768792 | 0,081600237 | 0,80768276 | Hs1bp3 | HCLS1 binding protein 3 |
| 17481632 | -5,227060346 | 0,001265484 | 0,095993501 | 0,80657294 | Olfr510 | olfactory receptor 510 |
| 17334419 | -7,49192625 | 0,000176649 | 0,058753337 | 0,80627617 | Msrb1 | methionine sulfoxide reductase B1 |
| 17495207 | -7,256807715 | 0,000204723 | 0,062295714 | 0,800682844 | 2310014F06Rik | RIKEN cDNA 2310014F06 gene |
| 17259475 | -5,294892089 | 0,001187309 | 0,093815338 | 0,792338987 | Lrrc45 | leucine rich repeat containing 45 |
| 17473477 | -5,772061854 | 0,000751084 | 0,081210084 | 0,783784091 | U2af2 | U2 small nuclear ribonucleoprotein auxiliary factor (U2AF) 2 |
| 17217887 | -4,974343153 | 0,001633899 | 0,097826161 | 0,775300932 | 4930596I21Rik | RIKEN cDNA 4930596I21 gene |
| 17296422 | -6,549200782 | 0,000375326 | 0,074038814 | 0,773952395 | Pelo | pelota homolog (Drosophila) |
| 17506369 | -6,738000916 | 0,000316155 | 0,070811709 | 0,768006566 | Zc3h18 | zinc finger CCCH-type containing 18 |
| 17262178 | -5,034364167 | 0,001540176 | 0,097524576 | 0,765810185 | Trim41 | tripartite motif-containing 41 |
| 17234711 | -5,669316077 | 0,000825804 | 0,083980931 | 0,765137462 | Gm10142 | predicted gene 10142 |
| 17244506 | -5,644908828 | 0,000846103 | 0,084494765 | 0,762041229 | Mrpl42 | mitochondrial ribosomal protein L42 |
| 17452194 | -6,387698454 | 0,000429314 | 0,07674456 | 0,753836045 | Erp29 | endoplasmic reticulum protein 29 |
| 17296128 | -5,707717265 | 0,000796866 | 0,082884125 | 0,747343206 | Gapt | Grb2-binding adaptor, transmembrane |
| 17383848 | -10,74790876 | 3,15291E-05 | 0,027251476 | 0,741433813 | Swi5 | SWI5 recombination repair homolog (yeast) |
| 17498059 | -5,431919004 | 0,001036142 | 0,089000756 | 0,740031916 | Krtap5-2 | keratin associated protein 5-2 |
| 17236077 | -6,221477007 | 0,000510513 | 0,080717083 | 0,739548197 | Ric8b | resistance to inhibitors of cholinesterase 8 homolog B (C. elegans) |
| 17470175 | -6,131860148 | 0,000550248 | 0,080776169 | 0,73954527 | Zfp248 | zinc finger protein 248 |
| 17287247 | -5,15516952 | 0,001370005 | 0,096476139 | 0,737989697 | 2310081J21Rik | RIKEN cDNA 2310081J21 gene |
| 17528430 | -5,112088093 | 0,001424857 | 0,096476139 | 0,730768773 | Tpm1 | tropomyosin 1, alpha |
| 17380268 | -6,235980138 | 0,000501874 | 0,080648038 | 0,729705006 | Vapb | vesicle-associated membrane protein, associated protein B and C |
| 17440745 | -5,565698702 | 0,000905706 | 0,084873601 | 0,729066335 | Gm15736 | predicted gene 15736 |
| 17329842 | -5,088061448 | 0,001458546 | 0,096536895 | 0,727438123 | Mir1946a | microRNA 1946a |
| 17365243 | -5,835863343 | 0,000713076 | 0,080976625 | 0,725561679 | Npm3 | nucleoplasmin 3 |
| 17467466 | -5,099053614 | 0,001442997 | 0,096476139 | 0,721848494 | Igkv4-55 | immunoglobulin kappa variable 4-55 |
| 17408813 | -5,366790579 | 0,001118204 | 0,091980518 | 0,716906834 | Cttnbp2nl | CTTNBP2 N-terminal like |
| 17219086 | -6,190495876 | 0,000526061 | 0,080717083 | 0,715544776 | Tmco1 | transmembrane and coiled-coil domains 1 |
| 17244341 | -5,770092046 | 0,00075238 | 0,081210084 | 0,715519882 | Elk3 | ELK3, member of ETS oncogene family |
| 17322254 | -5,167897919 | 0,001352297 | 0,096476139 | 0,714678626 | Npff | neuropeptide FF-amide peptide precursor |
| 17258615 | -4,983174887 | 0,00162051 | 0,097826161 | 0,70839807 | Gm11744 | predicted gene 11744 |
| 17237084 | -5,445528947 | 0,001019298 | 0,088888554 | 0,702515826 | Ccdc59 | coiled-coil domain containing 59 |
| 17517105 | -5,751221169 | 0,000767928 | 0,081600237 | 0,701555281 | Il18 | interleukin 18 |
| 17486874 | -5,381847693 | 0,001098336 | 0,091326244 | 0,697668412 | Ccdc9 | coiled-coil domain containing 9 |
| 17243659 | -5,317624063 | 0,001161826 | 0,093087581 | 0,69711834 | Gm6713 | predicted gene 6713 |
| 17254591 | -5,111272772 | 0,001425721 | 0,096476139 | 0,692868074 | Tbx2 | T-box 2 |
| 17536742 | -6,697325279 | 0,000326089 | 0,070811709 | 0,683861625 | Nono | non-POU-domain-containing, octamer binding protein |
| 17520856 | -6,67633391 | 0,000332999 | 0,070811709 | 0,678196667 | Amotl2 | angiomotin-like 2 |
| 17408074 | -5,134275193 | 0,001398079 | 0,096476139 | 0,677787116 | Polr3gl | polymerase (RNA) III (DNA directed) polypeptide G like |
| 17250236 | -5,743764089 | 0,000771816 | 0,081600237 | 0,677784883 | 4933439C10Rik | RIKEN cDNA 4933439C10 gene |
| 17214910 | -6,063503921 | 0,000587392 | 0,080976625 | 0,67622792 | Gm2427 | predicted gene 2427 |
| 17527495 | -5,358153832 | 0,001125978 | 0,092090263 | 0,667924383 | Commd4 | COMM domain containing 4 |
| 17520624 | -5,146475762 | 0,001383394 | 0,096476139 | 0,66694127 | Rbp1 | retinol binding protein 1, cellular |
| 17453454 | -5,276100621 | 0,001208472 | 0,094186645 | 0,66646612 | Eln | elastin |
| 17277521 | -5,995106041 | 0,000620649 | 0,080976625 | 0,664029815 | Vash1 | vasohibin 1 |
| 17349304 | -5,337752682 | 0,001143254 | 0,092637519 | 0,66378557 | Tslp | thymic stromal lymphopoietin |
| 17550042 | -6,198037932 | 0,00052347 | 0,080717083 | 0,65963552 | Mir1943 | microRNA 1943 |
| 17319205 | -6,198037932 | 0,00052347 | 0,080717083 | 0,65963552 | Mir1943 | microRNA 1943 |
| 17217619 | -5,423112655 | 0,001046508 | 0,089354471 | 0,656651815 | Phlda3 | pleckstrin homology-like domain, family A, member 3 |
| 17357597 | -5,672506897 | 0,000822349 | 0,083980931 | 0,649934897 | Slc15a3 | solute carrier family 15, member 3 |
| 17213189 | -5,400101218 | 0,001076309 | 0,090018052 | 0,643829403 | Gm20257 | caspase 8 pseudogene |
| 17401117 | -6,584363992 | 0,000361073 | 0,072732014 | 0,639396195 | Nras | neuroblastoma ras oncogene |
| 17270715 | -5,194373362 | 0,001306515 | 0,096069042 | 0,628000474 | Gm11651 | predicted gene 11651 |
| 17366918 | -5,180441052 | 0,001331133 | 0,096476139 | 0,622552549 | Mir466d | microRNA 466d |
| 17252013 | -7,018203775 | 0,000252665 | 0,070165936 | 0,612573124 | Arrb2 | arrestin, beta 2 |
| 17254171 | -5,225798416 | 0,001269371 | 0,095993501 | 0,60580987 | Slfn1 | schlafen 1 |
| 17333709 | -5,132370803 | 0,001399375 | 0,096476139 | 0,59901349 | Spaca6 | sperm acrosome associated 6 |
| 17431174 | -5,614726916 | 0,000869858 | 0,084494765 | 0,593866736 | Cd52 | CD52 antigen |
| 17548432 | -5,448719809 | 0,001014547 | 0,088744806 | 0,59141636 | Gm10091 | predicted gene 10091 |
| 17403237 | -5,057195389 | 0,001501304 | 0,096786837 | 0,586959168 | Gbp3 | guanylate binding protein 3 |
| 17357947 | -7,042141299 | 0,000248346 | 0,06964266 | 0,575545809 | Olfr1502 | olfactory receptor 1502 |
| 17466768 | -6,042861491 | 0,000599053 | 0,080976625 | 0,549191127 | Hnrnpa2b1 | heterogeneous nuclear ribonucleoprotein A2/B1 |
| 17448924 | -5,210166839 | 0,001285351 | 0,095993501 | 0,540414412 | Kdr | kinase insert domain protein receptor |
| 17491323 | -5,526778781 | 0,000941123 | 0,085732855 | 0,51999887 | Mrgpra2a | MAS-related GPR, member A2A |
| 17458362 | -5,457848769 | 0,001007636 | 0,088448594 | 0,500004863 | Gimap4 | GTPase, IMAP family member 4 |
| 17238367 | -6,458444801 | 0,000404696 | 0,074681937 | 0,49335415 | Stat2 | signal transducer and activator of transcription 2 |
| 17325324 | -6,872414721 | 0,000289809 | 0,070811709 | 0,484851567 | Stfa2l1 | stefin A2 like 1 |
| 17329298 | -6,225493105 | 0,000507921 | 0,080717083 | 0,479676207 | Etv5 | ets variant 5 |
| 17327557 | -7,775252466 | 0,000149871 | 0,057157851 | 0,46721545 | Mx2 | MX dynamin-like GTPase 2 |
| 17451930 | -5,984203798 | 0,000630582 | 0,080976625 | 0,460512146 | AW549542 | expressed sequence AW549542 |
| 17547909 | -6,147338094 | 0,000544633 | 0,080717083 | 0,446588513 | Gm19551 | predicted gene, 19551 |
| 17366932 | -4,992296959 | 0,001604098 | 0,097826161 | 0,419490919 | Mir466h | microRNA 466h |
| 17350925 | -5,171913899 | 0,001345818 | 0,096476139 | 0,374099363 | Iigp1 | interferon inducible GTPase 1 |
| 17254176 | -5,35773351 | 0,001126842 | 0,092090263 | 0,357441501 | Slfn4 | schlafen 4 |
| 17510345 | -5,345714464 | 0,00113548 | 0,092268955 | 0,274123389 | Bst2 | bone marrow stromal cell antigen 2 |
