# Supplementary material for: Concomitant deletion of HRAS and NRAS leads to pulmonary immaturity, respiratory failure and neonatal death in mice
Source: Cell Death Dis. 2019 Nov 4;10(11):838. doi: 10.1038/s41419-019-2075-2 (PMC6828777; doi:10.1038/s41419-019-2075-2)
Supplement: Supplementary file 4 — Supplementary Table 2 [file 41419_2019_2075_MOESM4_ESM.docx]

**Table S2. Functional annotation of differentially expressed genes (overexpressed and downregulated) in the lungs of HRAS/NRAS-DKO mice**

The GeneCodis (Gene Annotation Co-occurrence Discovery) functional annotation tool (<http://genecodis.cnb.csic.es/> ) was used to identify statistically significant functional associations linking particular gene subsets contained within the list of differentially expressed gene probesets identified in HRAS/NRAS-DKO lungs [FDR = 0.1; heatmap Fig 6A; Table S1 including 165 overexpressed (red) and 76 repressed (blue) genes] to specific cellular functionalities, including particular GO Biological Processes (BP) or Molecular Functions (MF), KEGG Signaling Pathways, and Transcriptional Factors that may account for regulation of expression of the corresponding groups of loci listed in each case, as indicated. Red: overexpressed genes. Blue: repressed genes.

The columns labelled “*Functional Category*”, “*KEGG Pathway*” and “*Transcription Factor*” identify the specific functional GO (BP or MF), KEGG or TF terms recognized in each case for the corresponding groups of loci listed under the column labelled “*Genes*”. The column labelled “*Number of Genes*” indicates the specific number of genes annotated to the indicated functionality, out of the total number (in parenthesis) of genes recognized by GeneCodis in the lists of differentially expressed, overexpressed (red) or repressed (blue) genes identified in HRas/NRas-DKO lungs. The column labelled “*Hypergeometric pValue*” refers to the statistical significance assignated by Genecodis to each of the functional associations identified.

**GO Biological Process (BP) Enrichment Analysis of 165 Genes Overexpressed in HRAS/NRAS-DKO Lungs**

| ***Items*** | ***Functional categorys*** | ***Number of genes*** | ***Hypergeom.***  ***pValue*** | ***Genes*** |
| --- | --- | --- | --- | --- |
| GO:0006810  GO:0015031  GO:0016192  GO:0006886  GO:0006811  GO:0015991  GO:0015992  GO 0055085  GO 0034220 | transport (BP)  protein transport (BP)  vesicle-mediated transport (BP)  intracellular protein transport (BP)  ion transport (BP)  ATP hydrolysis coupled proton transport (BP)  proton transport (BP)  transmembrane transport (BP)  ion transmembrane transport (BP) | 34 | 2.24556e-14 | Slc25a16, Pgap1, Atp5e, Scfd2, Derl1, Atp6v1b2, Clcn5, Ap2s1, Exoc5, Kcnma1, Gpr89, Kcnj9, Atp6v0a2, Stam2, Plekha8, Atg4b, Slc25a29, lc39a4, Mtx2, Uqcrh, Ndufa7, Slc25a46, Trappc4, Tomm20, Scn1a, Atp6v0e, Nipa1, Bcap29, Lmbrd1, Sec23a, Slc7a14, Glra3, Atg16l1, Ramp3 |
| GO:0008152  ~~GO 000658~~  ~~GO 0016567~~ | metabolic process (BP)  ~~proteolysis~~  ~~protein ubiquitination~~ | 21 | 1.35098e-05 | Mylip, March5, Rpap2, Pde2a, Lgmn, Ubr7, Atg4b, Pigk, Casp6, Glce, Ppp1ca, Fh1, Sptlc1, Ifnar1, Atp6v0e, Rnf167, Uhrf2, Sgpp1, Ptprz1, Uggt1, Ythdc2 |
| GO:0046034 | ATP metabolic process (BP) | 3 | 2.77057e-04 | Atp5e,Atp6v1b2,Ak3 |
| GO:0006508 | proteolysis (BP) | 8 | 3.36783e-03 | Psmb4,Dnpep,Psmb5,Lgmn,Usp38,Atg4b, Pigk,Casp6 |
| GO:0006511 | ubiquitin-dependent protein catabolic process (BP) | 3 | 1.59383e-02 | Usp38, Fbxo8, Uhrf2 |
| GO:0005975  GO 005977 | carbohydrate metabolic process (BP)  glycogen metabolic process (BP) | 5 | 1.04802e-03 | Mgat4a, Ppp1ca, Phkg2, Alg13, Ppp1cb |
| GO:0006470  GO:0007049 | protein dephosphorylation (BP),cell cycle (BP) | 3 | 1.76194e-05 | Ppm1d, Ppp1ca, Ppp1cb |
| GO:0006626 | protein targeting to mitochondrion (BP) | 3 | 7.58745e-05 | Pde2a, Mtx2, Tomm20 |
| GO:0042981 | regulation of apoptotic process (BP) | 3 | 3.19694e-02 | Ppp2r1a, Bnip3, Casp6 |
| GO:0006915 ~~GO:0006917~~ | apoptotic process (BP) | 7 | 6.9995e-03 | Bnip3, Clptm1l, Casp6, Perp, Bcap29, Sgpp1, Ddit4 |
| GO:0006914  GO:0000045 | autophagy (BP),autophagic vacuole assembly (BP) | 3 | 2.89647e-05 | Wipi2, Atg4b, Atg16l1 |
| GO:0006506 | GPI anchor biosynthetic process (BP) | 3 | 3.06854e-04 | Pgap1, Dpm3, Pigk |

**GO Molecular Function (MF) Enrichment Analysis of 165 Genes Overexpressed in HRAS/NRAS-DKO Lungs**

| ***Items*** | ***Functional category*** | ***Number of genes*** | ***Hypergeom.***  ***pValue*** | ***Genes*** |
| --- | --- | --- | --- | --- |
| GO:0016787 | hydrolase activity (MF) | 23 / 165 | 1.86758e-07 | Pgap1, Atp5e, Psmb4, Dnpep, Atp6v1b2, Psmb5, Rpap2, Tsen34, Pde2a, Lgmn, Usp38, Atg4b, Pigk, Acer3, Ppm1d, Casp6, Ppp1ca, Atp6v0e, Sgpp1, Gdpd1, Ptprz1, Ythdc2, Ppp1cb |
| GO:0046872 | metal ion binding (MF) | 23 /165 | 2.65291e-03 | Dnpep, Mylip, Zxdc, March5, Mgat4a, Rpap2, Kcnma1, Pde2a, Ubr7, Ch25h, Zfp35, Cdo1, Zfp944, Rev1, Ppm1d, Ppp1ca, Polr3k, Rnf167, Uhrf2, Gdpd1, Papd5, Ppp1cb, Gm15446 |
| GO:0005216 | ion channel activity (MF) | 6 / 165 | 2.57536e-03 | Clcn5, Kcnma1, Gpr89, Kcnj9, Scn1a, Glra3 |

**KEGG Pathways Enrichment analysis of 165 Genes Overexpressed in HRAS/NRAS-DKO Lungs**

| ***Items*** | ***KEGG pathways*** | ***Number of genes*** | ***Hypergeom. pValue*** | ***Genes*** |
| --- | --- | --- | --- | --- |
| Kegg:00190 | Oxidative phosphorylation | 7 | 2.55981e-05 | Atp6v0a2, Uqcrh, Atp6v0e, Ndufa7, Atp6v1b2, Atp5e, Ak3 |
| Kegg:00600 | Sphingolipid metabolism | 5 | 3.16363e-05 | Degs1, Sgpp1, Acer3, Sptlc1, Cers5 |
| Kegg:00510 | N-Glycan biosynthesis | 3 | 1.38934e-03 | Alg13, Mgat4a, Dpm3 |
| Kegg:04910 | Insulin signaling pathway | 4 | 3.00635e-03 | Ppp1cb, Ppp1ca, Phkg2, Socs2 |
| Kegg:04270 | Vascular smooth muscle contraction | 4 | 1.68019e-03 | Ppp1cb, Kcnma1, Ppp1ca, Ramp3 |
| Kegg:03050 | Proteasome | 3 | 1.02209e-03 | Psmb5, Psmd11, Psmb4 |
| Kegg:04141 | Protein processing in endoplasmic reticulum | 4 | 5.61344e-03 | Sec23a, Derl1, Dnajb1, Uggt1 |

**Functional annotation to “TRANSCRIPTION FACTORS” of 165 genes overexpressed in the lungs of HRAS/NRAS-DKO mice**

| ***Transcription Factor*** | ***Number of genes*** | ***Hypergeom pValue*** | ***Genes*** |
| --- | --- | --- | --- |
| V$ELK1_02 | 10 / 165 | 1.88805e-03 | Ap2s1, Scfd2, Dnajb1, Uqcrh, Trappc4, Mtx2, Spg21, Tomm20, Snrpb, Fbxo8 |
| V$ERR1_Q2 | 9 / 165 | 8.11141e-03 | Uhrf2, Ppp1cb, Scfd2, Bnip3, Uqcrh, Actr1a, Mtx2, Got1, Socs2 |
| V$AP1_C | 9 / 165 | 1.06567e-02 | Clcn5, Bnip3, Vat1, Btbd11, Atp6v1b2, Gfra1, Perp, Psmd11, Gpr111 |
| V$LEF1_Q2 | 18 (165) | 2.25861e-03 | Cdo1, Uhrf2, Clcn5, Papd5, Ppp1cb, Tacstd2, Nup98, Bnip3, Dctn3, Pgap1, Vat1, Actr1a, Gfra1, Psmb5, Psmd11, Ddit4, Socs2, Psmb4 |
| V$NFY_Q6_01 | 9 (165) | 1.07351e-02 | Slc39a4, Nup98, Dnajb1, Actr1a, Ppp2r1a, Ppp1r7, Glra3, Got1, Ppm1d |
| V$MYB_Q6 | 4 | 6.37742e-03 | Slc39a4, Ssbp2, Cldn8, Socs2 |
| V$CEBPA_01 | 4 | 7.79868e-03 | Clock, Cldn8, Ppp1cb, Nfat5 |
| V$HSF1_01 | 5 | 1.61305e-02 | Dnajb1, Gipc2, Glra3, Socs2, Ppm1d |
| V$MYOGENIN_Q6 | 4 | 7.49923e-03 | Bnip3, Btbd11, Gfra1, Ddit4 |
| V$YY1_Q6 | 5 | 1.14333e-02 | Sec23a, Kcnma1, Uqcrh, Prpsap2, Psmb5 |
| V$FOXJ2_01 | 5 | 3.79737e-04 | Cldn8, Ppp1cb, Gfra1, Ch25h, Ppm1d |
| V$HFH1_01 | 4 | 8.26242e-03 | Cldn8, Ppp1cb, Tacstd2, Ppm1d |

**Go Biological Process (BP) Enrichment Analysis of 76 Genes Downregulated in HRAS/NRAS-DKO Lungs**

| ***Items*** | ***Functional category*** | ***Number of genes*** | ***Hypergeom. pValue*** | ***Genes*** |
| --- | --- | --- | --- | --- |
| GO:0007204 | elevation of cytosolic calcium ion concentration (BP) | 3 | 1.24038e-03 | Kdr, Cd52, Npff |
| GO:0006397,  GO:0008380 | mRNA processing (BP),RNA splicing (BP) | 4 | 6.05231e-04 | Hnrnpa2b1, Nono, U2af2, Prpf3 |

**Go Molecular Function (MF) Enrichment Analysis of 76 Genes Downregulated in HRAS/NRAS-DKO Lungs**

| ***Items*** | ***Functional category*** | ***Number of genes*** | ***Hypergeom. pValue*** | ***Genes*** |
| --- | --- | --- | --- | --- |
| GO:0000166 | nucleotide binding (MF) | 10 / 76 | 0.00675264 | Iigp1, Hnrnpa2b1, Kdr,Gbp3, Nono, Gimap4, Gm6713, U2af2, Mx2, Nras |

**KEGG Pathways Enrichment analysis of 76 Genes Downregulated in HRAS/NRAS-DKO Lungs**

| ***Items*** | ***KEGG pathways*** | ***Number of genes*** | ***Hypergeom. pValue*** | ***Genes*** |
| --- | --- | --- | --- | --- |
| **Kegg:04062** | **Chemokine signaling pathway** | **3** | **5.5719e-03** | **Arrb2, Stat2, Nras** |
| **Kegg:04060** | **Cytokine-cytokine receptor interaction** | **3** | **1.33254e-02** | **Tslp, Il18, Kdr** |

**Functional annotation to “TRANSCRIPTION FACTORS” of 76 genes repressed in the lungs of HRAS/NRAS-DKO mice**

| ***Transcription Factor*** | ***Number of genes /*** | ***Hypergeom. pValue*** | ***Genes*** |
| --- | --- | --- | --- |
| V$SP1_Q6 | 12 / 76 | 0.000779745 | Elk3, Arrb2, Commd4, Eln, Npm3, Vapb, Etv5, Tbx2, Prpf3, Tmlhe, Trim41, Nras |
| V$GABP_B | 7 (76) | 0.000117672 | Elk3, Arrb2, Etv5, Trim41, U2af2, Sox10, Nras |
| V$AML1_01 | 5 | 2.88692e-05 | Slc15a3, Stat2, Tpm1, Mrpl42, Nras |
| V$AML1_Q6 | 5 | 2.88692e-05 | Slc15a3, Stat2, Tpm1, Mrpl42, Nras |
| V$ETS_Q4 | 4 | 0.000425533 | Elk3, Arrb2, Etv5, Trim41 |
