# Supplementary material for: Concomitant deletion of HRAS and NRAS leads to pulmonary immaturity, respiratory failure and neonatal death in mice
Source: Cell Death Dis. 2019 Nov 4;10(11):838. doi: 10.1038/s41419-019-2075-2 (PMC6828777; doi:10.1038/s41419-019-2075-2)
Supplement: Supplementary file 5 — Supplementary Table 3 [file 41419_2019_2075_MOESM5_ESM.docx]

**Table S3. Differential gene expression in the lungs of HRAS/NRAS-DKO mice treated antenatally with dexamethasone.**

List of 509 differentially expressed gene probesets identified by means of SAM contrasts (FDR=0.15) in multiclass comparison (**Fig. 6B** heatmap) between the transcriptional profiles of lungs from newborn (P0) DKO mice that had been treated *in utero* with dexamethasone (injections at E17.5 and E18.5) and the transcriptional profiles of untreated (P0) DKO littermates that were generated by RNA microarray hybridization assays using GeneChip(R) Mouse Gene 2.0 ST Arrays. The differentially expressed loci are identified by *Affymetrix Probeset ID, Genename Symbol* or *Description* and listed according to their degree of overexpression or repression in dexamethasone-treated lung tissue of DKO mice. *d-value* is a parameter measuring the statistical distance separating the calculated expression value of each gene probeset from the null hypothesis (no-change). *q-value* is the estimated FDR at the largest p-value for which the probe set would be statistically significant. *R-fold* is a measure of the fold change of a probeset in the collection of microarrays provided by the SAM algorithm. Entries in red denote overexpression. Entries in green denote transcriptional repression. The data list organized here from maximal to minimal d-values. Components of ceramide/sphingosine metabolism are printed in bold.

| **probeset ID** | **d.value** | **p.value** | **q.value** | **R.fold** | **Genename** | **Description** |
| --- | --- | --- | --- | --- | --- | --- |
| 17357648 | 5,800980166 | 3,88715E-05 | 0,096074093 | 1,920712178 | Ms4a4c | membrane-spanning 4-domains, subfamily A, member 4C |
| 17259311 | 5,450425722 | 5,83073E-05 | 0,096074093 | 1,568655314 | 0610009L18Rik | RIKEN cDNA 0610009L18 gene |
| 17400622 | 5,443224101 | 5,91711E-05 | 0,096074093 | 1,694496031 | Lix1l | Lix1-like |
| 17509721 | 5,308217228 | 7,12644E-05 | 0,096074093 | 1,495688964 | Tufm | Tu translation elongation factor, mitochondrial |
| 17443683 | 5,126100134 | 8,59492E-05 | 0,096074093 | 1,620692856 | Ppp1r35 | protein phosphatase 1, regulatory subunit 35 |
| 17240303 | 4,936497607 | 0,000108408 | 0,096074093 | 1,887549863 | G630090E17Rik | RIKEN cDNA G630090E17 gene |
| 17458362 | 4,765692033 | 0,000141233 | 0,104761644 | 2,246207919 | Gimap4 | GTPase, IMAP family member 4 |
| 17276182 | 4,755402964 | 0,000144688 | 0,104761644 | 1,552627184 | Jkamp | JNK1/MAPK8-associated membrane protein |
| 17357910 | 4,526359256 | 0,000206451 | 0,116707116 | 2,052924326 | Olfr1463 | olfactory receptor 1463 |
| 17411492 | 4,518059895 | 0,000209474 | 0,116707116 | 1,873515792 | Gm11783 | predicted gene 11783 |
| 17402165 | 4,478487392 | 0,000228478 | 0,117965976 | 2,111983968 | A530020G20Rik | RIKEN cDNA A530020G20 gene |
| 17376538 | 4,376356888 | 0,000262599 | 0,124070176 | 2,076853064 | 4930425F17Rik | RIKEN cDNA 4930425F17 gene |
| 17244341 | 4,286378482 | 0,000302766 | 0,12603958 | 1,575044061 | Elk3 | ELK3, member of ETS oncogene family |
| 17394829 | 4,152856254 | 0,000367984 | 0,127996194 | 1,513665594 | Atp9a | ATPase, class II, type 9A |
| 17433310 | 4,031688191 | 0,000440976 | 0,132184757 | 1,30850635 | Park7 | Parkinson disease (autosomal recessive, early onset) 7 |
| 17466294 | 3,975815699 | 0,000480711 | 0,132184757 | 1,397874208 | Try5 | trypsin 5 |
| 17255215 | 3,922649481 | 0,000522174 | 0,132737203 | 2,013953925 | Ankrd40 | ankyrin repeat domain 40 |
| 17284702 | 3,862915192 | 0,000574003 | 0,132737203 | 1,533029579 | Gm10421 | predicted gene 10421 |
| 17530915 | 3,852809951 | 0,000580913 | 0,132737203 | 2,122660087 | Gm9917 | predicted gene 9917 |
| 17393225 | 3,833965601 | 0,000595166 | 0,132737203 | 1,71662334 | Pigu | phosphatidylinositol glycan anchor biosynthesis, class U |
| 17234024 | 3,796165761 | 0,000629719 | 0,132737203 | 3,556466551 | Rtkn2 | rhotekin 2 |
| 17234711 | 3,781923723 | 0,000647859 | 0,132737203 | 1,309959767 | Gm10142 | predicted gene 10142 |
| 17511677 | 3,764405546 | 0,000661248 | 0,133061743 | 2,112141857 | Ces1c | carboxylesterase 1C |
| 17386177 | 3,736929088 | 0,000693641 | 0,134289173 | 1,488797143 | Abcb11 | ATP-binding cassette, sub-family B (MDR/TAP), member 11 |
| 17548940 | 3,728646209 | 0,000700551 | 0,134587111 | 1,414644871 | Soat1 | sterol O-acyltransferase 1 |
| 17484682 | 3,72186879 | 0,000709621 | 0,1347626 | 1,680331399 | Athl1 | ATH1, acid trehalase-like 1 (yeast) |
| 17442332 | 3,708490555 | 0,000725602 | 0,136174199 | 1,367416124 | Kntc1 | kinetochore associated 1 |
| 17222825 | 3,688469985 | 0,000751948 | 0,136401701 | 2,799199546 | Nabp1 | nucleic acid binding protein 1 |
| 17384205 | 3,676864141 | 0,000765769 | 0,136733781 | 1,52783397 | Mvb12b | multivesicular body subunit 12B |
| 17380567 | 3,655483338 | 0,000792547 | 0,137836277 | 2,303066348 | Gm14322 | predicted gene 14322 |
| 17331848 | 3,587883431 | 0,000886702 | 0,139806133 | 1,297769639 | 2310061N02Rik | RIKEN cDNA 2310061N02 gene |
| 17457609 | 3,576813513 | 0,00090873 | 0,139806133 | 1,452905967 | Mgam | maltase-glucoamylase |
| 17516960 | 3,564595157 | 0,000929893 | 0,139806133 | 1,483860375 | Cadm1 | cell adhesion molecule 1 |
| 17217946 | 3,527653586 | 0,000981722 | 0,139806133 | 1,32796367 | F13b | coagulation factor XIII, beta subunit |
| 17404923 | 3,511912934 | 0,001011523 | 0,139806133 | 1,338392838 | Gm5148 | predicted gene 5148 |
| 17380555 | 3,511749727 | 0,001011955 | 0,139806133 | 2,070276047 | Gm14403 | predicted gene 14403 |
| 17211375 | 3,483268058 | 0,00106292 | 0,139806133 | 1,342887651 | Paqr8 | progestin and adipoQ receptor family member VIII |
| 17399314 | 3,456364806 | 0,001115613 | 0,139806133 | 1,565545328 | Fam189b | family with sequence similarity 189, member B |
| 17474906 | 3,452573562 | 0,001125546 | 0,139806133 | 1,307430512 | Zfp114 | zinc finger protein 114 |
| 17265030 | 3,441950465 | 0,001144982 | 0,139806133 | 1,451421797 | Acap1 | ArfGAP with coiled-coil, ankyrin repeat and PH domains 1 |
| 17217590 | 3,433246226 | 0,001163986 | 0,139806133 | 1,685692046 | Arl8a | ADP-ribosylation factor-like 8A |
| 17344251 | 3,396121838 | 0,001243889 | 0,139806133 | 1,608600529 | Prrc2a | proline-rich coiled-coil 2A |
| 17354653 | 3,383791231 | 0,00127585 | 0,139806133 | 1,667081798 | Tcof1 | Treacher Collins Franceschetti syndrome 1, homolog |
| 17400375 | 3,369432449 | 0,001309538 | 0,139806133 | 1,422660284 | Ctss | cathepsin S |
| 17400072 | 3,3679807 | 0,001313857 | 0,139806133 | 1,284600442 | Tdrkh | tudor and KH domain containing protein |
| 17333731 | 3,361028777 | 0,001329838 | 0,139806133 | 1,711764232 | Fpr2 | formyl peptide receptor 2 |
| 17267184 | 3,359590182 | 0,001335453 | 0,139806133 | 1,485165506 | Mir5110 | microRNA 5110 |
| 17430521 | 3,350265516 | 0,001360503 | 0,139806133 | 1,329751953 | Tmem39b | transmembrane protein 39b |
| 17350921 | 3,335498453 | 0,001404989 | 0,139806133 | 1,995201274 | F830016B08Rik | RIKEN cDNA F830016B08 gene |
| 17486299 | 3,319700979 | 0,001440406 | 0,139806133 | 1,429051565 | Vmn2r45 | vomeronasal 2, receptor 45 |
| 17501096 | 3,316466258 | 0,001449476 | 0,139806133 | 1,369257681 | Cldn24 | claudin 24 |
| 17416460 | -2,497229696 | 0,0072206 | 0,18966393 | 0,581152779 | Yipf1 | Yip1 domain family, member 1 |
| 17349926 | -2,497634313 | 0,007209803 | 0,189599092 | 0,626549827 | Pcdhb17 | protocadherin beta 17 |
| 17361805 | -2,498518842 | 0,007197709 | 0,189514594 | 0,552596624 | Neat1 | nuclear paraspeckle assembly transcript 1 (non-protein coding) |
| 17245248 | -2,499813117 | 0,00717741 | 0,189281753 | 0,53043319 | Mdm2 | transformed mouse 3T3 cell double minute 2 |
| 17419025 | -2,501624413 | 0,007154951 | 0,189281753 | 0,74309541 | Tmem234 | transmembrane protein 234 |
| 17484140 | -2,501726179 | 0,007153655 | 0,189281753 | 0,757259058 | Bccip | BRCA2 and CDKN1A interacting protein |
| 17290603 | -2,508650142 | 0,007056476 | 0,188347263 | 0,559173144 | Actn2 | actinin alpha 2 |
| 17514257 | -2,509510044 | 0,007042655 | 0,188130325 | 0,668267065 | Rbm34 | RNA binding motif protein 34 |
| 17374612 | -2,509875138 | 0,00703704 | 0,188130325 | 0,66473691 | Phgr1 | proline/histidine/glycine-rich 1 |
| 17514247 | -2,510042233 | 0,007034881 | 0,188130325 | 0,724393976 | Tomm20 | translocase of outer mitochondrial membrane 20 homolog (yeast) |
| 17305243 | -2,512314764 | 0,007007239 | 0,187720276 | 0,632938723 | Sftpd | surfactant associated protein D |
| 17444664 | -2,512425227 | 0,007006375 | 0,187720276 | 0,529707651 | Zfp655 | zinc finger protein 655 |
| 17407643 | -2,51351983 | 0,006988667 | 0,187598072 | 0,783699822 | B230398E01Rik | RIKEN cDNA B230398E01 gene |
| 17239113 | -2,5141921 | 0,006980029 | 0,187518528 | 0,592007731 | Sash1 | SAM and SH3 domain containing 1 |
| 17305856 | -2,516407566 | 0,00694634 | 0,186765324 | 0,63893514 | Exoc5 | exocyst complex component 5 |
| 17454345 | -2,519971846 | 0,00689883 | 0,185801914 | 0,797963632 | Pdgfa | platelet derived growth factor, alpha |
| 17533553 | -2,520733979 | 0,006886737 | 0,185767399 | 0,640840867 | Jade3 | jade family PHD finger 3 |
| 17369821 | -2,52119706 | 0,00688069 | 0,185767399 | 0,671838754 | Ptges2 | prostaglandin E synthase 2 |
| 17438189 | -2,52214049 | 0,006866437 | 0,185767399 | 0,579690564 | Rasl11b | RAS-like, family 11, member B |
| 17333946 | -2,524315277 | 0,006842251 | 0,18547585 | 0,748224926 | Zfp948 | zinc finger protein 948 |
| 17329692 | -2,524740888 | 0,006831453 | 0,18547585 | 0,557148174 | Lsg1 | large subunit GTPase 1 homolog (S. cerevisiae) |
| 17301926 | -2,52905349 | 0,006778329 | 0,184499854 | 0,345126777 | Htr2a | 5-hydroxytryptamine (serotonin) receptor 2A |
| 17248592 | -2,529282868 | 0,006775737 | 0,184499854 | 0,599455873 | Slu7 | SLU7 splicing factor homolog (S. cerevisiae) |
| 17502378 | -2,530378383 | 0,006755438 | 0,184499854 | 0,646333559 | Tpm4 | tropomyosin 4 |
| 17539898 | -2,531075478 | 0,006745936 | 0,184499854 | 0,628054031 | Ccdc120 | coiled-coil domain containing 120 |
| 17547602 | -2,531672848 | 0,00673773 | 0,184499854 | 0,463432067 | Gm10099 | predicted gene 10099 |
| 17548865 | -2,532814626 | 0,006719158 | 0,184499854 | 0,730586649 | Gm8692 | predicted gene 8692 |
| 17217666 | -2,532845692 | 0,006718726 | 0,184499854 | 0,721297613 | Tmem9 | transmembrane protein 9 |
| 17252183 | -2,533220175 | 0,006711815 | 0,184499854 | 0,563148488 | Eno3 | enolase 3, beta muscle |
| 17488975 | -2,539511227 | 0,006619387 | 0,183666593 | 0,571852116 | Tbcb | tubulin folding cofactor B |
| 17211700 | -2,540550192 | 0,006605998 | 0,183666593 | 0,745274987 | Imp4 | IMP4, U3 small nucleolar ribonucleoprotein, homolog (yeast) |
| 17354764 | -2,541071114 | 0,006598656 | 0,183666593 | 0,673204769 | Arhgef37 | Rho guanine nucleotide exchange factor (GEF) 37 |
| **17415219** | **-2,541925716** | **0,006586563** | **0,183666593** | **0,764944787** | **Acer2** | **alkaline ceramidase 2** |
| 17301576 | -2,54257559 | 0,006575333 | 0,183664448 | 0,51894453 | Gnrh1 | gonadotropin releasing hormone 1 |
| 17263837 | -2,546844978 | 0,006529119 | 0,182803277 | 0,764126348 | Epn2 | epsin 2 |
| 17388551 | -2,546893617 | 0,006527823 | 0,182803277 | 0,729518007 | Alkbh3 | alkB, alkylation repair homolog 3 (E. coli) |
| 17401041 | -2,54718384 | 0,006524368 | 0,182803277 | 0,64039461 | Casq2 | calsequestrin 2 |
| 17274415 | -2,547631489 | 0,006518753 | 0,182803277 | 0,782585177 | Asap2 | ArfGAP with SH3 domain, ankyrin repeat and PH domain 2 |
| 17323192 | -2,548586565 | 0,006509683 | 0,182803277 | 0,665701162 | Snai2 | snail family zinc finger 2 |
| 17497044 | -2,548985289 | 0,006503637 | 0,182803277 | 0,620950933 | Ikzf5 | IKAROS family zinc finger 5 |
| 17310432 | -2,553274449 | 0,006436259 | 0,181777348 | 0,64087704 | Mtmr12 | myotubularin related protein 12 |
| 17473219 | -2,553384254 | 0,006434964 | 0,181777348 | 0,807714072 | Tsen34 | tRNA splicing endonuclease 34 homolog (S. cerevisiae) |
| 17497421 | -2,554435716 | 0,006423302 | 0,181722039 | 0,65820401 | Bnip3 | BCL2/adenovirus E1B interacting protein 3 |
| 17497687 | -2,555936938 | 0,006401707 | 0,181266279 | 0,642662774 | Bet1l | blocked early in transport 1 homolog (S. cerevisiae)-like |
| 17360216 | -2,556471106 | 0,00639566 | 0,181250378 | 0,580484835 | Gsto1 | glutathione S-transferase omega 1 |
| 17399914 | -2,556684235 | 0,006391773 | 0,181250378 | 0,728164336 | Lce3d | late cornified envelope 3D |
| 17280247 | -2,557542789 | 0,00637968 | 0,181250378 | 0,808351755 | 6030426L16Rik | RIKEN cDNA 6030426L16 gene |
| 17338562 | -2,559290579 | 0,006358516 | 0,181250378 | 0,591810537 | Rab5a | RAB5A, member RAS oncogene family |
| 17455231 | -2,564868215 | 0,00628682 | 0,181250378 | 0,539023618 | B230303O12Rik | RIKEN cDNA B230303O12 gene |
| 17223793 | -2,566114705 | 0,006272135 | 0,181250378 | 0,538160081 | Fzd5 | frizzled homolog 5 (Drosophila) |
| 17511259 | -2,56785717 | 0,006251404 | 0,181250378 | 0,616698437 | Junb | jun B proto-oncogene |
| 17498032 | -2,568871151 | 0,00623931 | 0,181250378 | 0,59953482 | Mob2 | MOB kinase activator 2 |
| 17256691 | -2,56991057 | 0,006227649 | 0,181250378 | 0,704756948 | Aoc2 | amine oxidase, copper containing 2 (retina-specific) |
| 17245539 | -2,570650658 | 0,006219011 | 0,181250378 | 0,683037052 | Srgap1 | SLIT-ROBO Rho GTPase activating protein 1 |
| 17327450 | -2,570690228 | 0,006217715 | 0,181250378 | 0,658200203 | Kcnj15 | potassium inwardly-rectifying channel, subfamily J, member 15 |
| 17334749 | -2,570763036 | 0,006217283 | 0,181250378 | 0,7656193 | Narfl | nuclear prelamin A recognition factor-like |
| 17319554 | -2,571781758 | 0,006201303 | 0,181250378 | 0,651679162 | Pmm1 | phosphomannomutase 1 |
| 17344990 | -2,571962197 | 0,006199575 | 0,181250378 | 0,53083904 | Cenpq | centromere protein Q |
| 17339574 | -2,574694349 | 0,006167614 | 0,181250378 | 0,541281906 | Ehd3 | EH-domain containing 3 |
| 17326816 | -2,578933327 | 0,006107579 | 0,181229766 | 0,716719895 | Gabpa | GA repeat binding protein, alpha |
| 17292800 | -2,578941992 | 0,006106715 | 0,181229766 | 0,672990221 | Uimc1 | ubiquitin interaction motif containing 1 |
| 17359449 | -2,580315302 | 0,006080369 | 0,181171751 | 0,652432117 | Gm340 | predicted gene 340 |
| 17519568 | -2,584695794 | 0,006023789 | 0,180135616 | 0,78242384 | Gm19569 | predicted gene, 19569 |
| 17523281 | -2,58517594 | 0,006017311 | 0,180104871 | 0,751641644 | Trak1 | trafficking protein, kinesin binding 1 |
| 17450727 | -2,589900355 | 0,005959435 | 0,179280713 | 0,555872881 | Tmed5 | transmembrane emp24 protein transport domain containing 5 |
| 17496183 | -2,591950462 | 0,005929634 | 0,178776083 | 0,735562411 | Nfatc2ip | nuclear factor of activated T cells, cytoplasmic, calcineurin dependent 2 interacting protein |
| 17328451 | -2,592978075 | 0,005920996 | 0,178776083 | 0,635783393 | Mzt2 | mitotic spindle organizing protein 2 |
| 17211405 | -2,595194891 | 0,005894218 | 0,178776083 | 0,729772749 | Gsta3 | glutathione S-transferase, alpha 3 |
| 17376252 | -2,60021396 | 0,005841093 | 0,178220547 | 0,6842111 | Nop56 | NOP56 ribonucleoprotein |
| 17526614 | -2,604116768 | 0,005794879 | 0,177630577 | 0,669812652 | Pafah1b2 | platelet-activating factor acetylhydrolase, isoform 1b, subunit 2 |
| 17222001 | -2,604622014 | 0,005791424 | 0,177630577 | 0,668269592 | Prim2 | DNA primase, p58 subunit |
| 17510563 | -2,606851859 | 0,005768965 | 0,177630577 | 0,737027415 | Slc35e1 | solute carrier family 35, member E1 |
| 17379417 | -2,61233766 | 0,005703747 | 0,17675478 | 0,633331235 | Sys1 | SYS1 Golgi-localized integral membrane protein homolog (S. cerevisiae) |
| 17366039 | -2,613876477 | 0,005682584 | 0,176641293 | 0,683774407 | Pdzd8 | PDZ domain containing 8 |
| 17299924 | -2,615495996 | 0,00566574 | 0,176454732 | 0,632618372 | Rps19 | ribosomal protein S19 |
| 17299847 | -2,615495996 | 0,00566574 | 0,176454732 | 0,632618372 | Rps19 | ribosomal protein S19 |
| 17234042 | -2,616348527 | 0,005656238 | 0,176454732 | 0,692929988 | Rhobtb1 | Rho-related BTB domain containing 1 |
| 17323759 | -2,616492732 | 0,005655374 | 0,176454732 | 0,716603763 | Ufd1l | ubiquitin fusion degradation 1 like |
| 17302802 | -2,618405952 | 0,005631619 | 0,176389566 | 0,787319767 | Farp1 | FERM, RhoGEF (Arhgef) and pleckstrin domain protein 1 (chondrocyte-derived) |
| 17277084 | -2,619507906 | 0,005622117 | 0,176259024 | 0,728845203 | Psen1 | presenilin 1 |
| 17478864 | -2,620770381 | 0,005610456 | 0,176060466 | 0,631549514 | Mtmr10 | myotubularin related protein 10 |
| 17454416 | -2,622212535 | 0,00559793 | 0,175834397 | 0,366636307 | Zfand2a | zinc finger, AN1-type domain 2A |
| 17244175 | -2,624796715 | 0,005565969 | 0,175330474 | 0,70873077 | Slc25a3 | solute carrier family 25 (mitochondrial carrier, phosphate carrier), member 3 |
| 17463883 | -2,625266183 | 0,00556165 | 0,175330474 | 0,662442292 | Strap | serine/threonine kinase receptor associated protein |
| 17522971 | -2,625563305 | 0,005555604 | 0,175330474 | 0,662778337 | Vill | villin-like |
| 17337513 | -2,625791271 | 0,005553444 | 0,175330474 | 0,766430093 | Gabbr1 | gamma-aminobutyric acid (GABA) B receptor, 1 |
| 17237851 | -2,626493184 | 0,005543942 | 0,175305073 | 0,785328727 | Avil | advillin |
| 17442909 | -2,627890648 | 0,005526234 | 0,175281605 | 0,678336469 | Gbas | glioblastoma amplified sequence |
| 17371661 | -2,628673989 | 0,005515868 | 0,175255894 | 0,79092066 | Gorasp2 | golgi reassembly stacking protein 2 |
| 17448607 | -2,630949662 | 0,005492113 | 0,175255894 | 0,650875127 | Commd8 | COMM domain containing 8 |
| 17514745 | -2,631887548 | 0,005483907 | 0,175255894 | 0,617876931 | Cwc15 | CWC15 homolog (S. cerevisiae) |
| 17515045 | -2,633336813 | 0,005465335 | 0,175255894 | 0,581788798 | Ppan | peter pan homolog (Drosophila) |
| 17368947 | -2,63336743 | 0,005464903 | 0,175255894 | 0,743592136 | Odf2 | outer dense fiber of sperm tails 2 |
| 17271931 | -2,63411414 | 0,005456697 | 0,175255894 | 0,703313195 | Sumo2 | SMT3 suppressor of mif two 3 homolog 2 (yeast) |
| 17326801 | -2,636456437 | 0,005432079 | 0,175255894 | 0,617708271 | Jam2 | junction adhesion molecule 2 |
| 17443133 | -2,63719045 | 0,0054256 | 0,175255894 | 0,731736973 | Wbscr27 | Williams Beuren syndrome chromosome region 27 (human) |
| 17249140 | -2,638850463 | 0,005407028 | 0,175255894 | 0,748149099 | 3010026O09Rik | RIKEN cDNA 3010026O09 gene |
| 17492239 | -2,638872953 | 0,005406164 | 0,175255894 | 0,696284176 | AU020206 | expressed sequence AU020206 |
| 17412552 | -2,641472762 | 0,005374203 | 0,175255894 | 0,646276466 | Ube2j1 | ubiquitin-conjugating enzyme E2J 1 |
| 17262768 | -2,641813714 | 0,005370316 | 0,175255894 | 0,648609204 | Slc22a21 | solute carrier family 22 (organic cation transporter), member 21 |
| 17533234 | -2,642946783 | 0,005358223 | 0,175255894 | 0,661347211 | Sytl5 | synaptotagmin-like 5 |
| 17320100 | -2,643270686 | 0,005354767 | 0,175255894 | 0,773630324 | Pkdrej | polycystic kidney disease (polycystin) and REJ (sperm receptor for egg jelly homolog, sea urchin) |
| 17318587 | -2,644250407 | 0,005342674 | 0,175255894 | 0,576918048 | Slc39a4 | solute carrier family 39 (zinc transporter), member 4 |
| 17452396 | -2,644408803 | 0,005340083 | 0,175255894 | 0,784052782 | Fam216a | family with sequence similarity 216, member A |
| 17370309 | -2,647746974 | 0,005308553 | 0,174901557 | 0,556445032 | Ptgs1 | prostaglandin-endoperoxide synthase 1 |
| 17469556 | -2,647751481 | 0,005308122 | 0,174901557 | 0,684449735 | Crbn | cereblon |
| 17245850 | -2,648349665 | 0,005300779 | 0,174901557 | 0,65999114 | Mars | methionine-tRNA synthetase |
| 17288387 | -2,648493938 | 0,00529862 | 0,174901557 | 0,761178211 | Nsun2 | NOL1/NOP2/Sun domain family member 2 |
| 17304860 | -2,649685851 | 0,005286526 | 0,174901557 | 0,663727425 | Timm23 | translocase of inner mitochondrial membrane 23 |
| 17349745 | -2,650716874 | 0,005277024 | 0,174901557 | 0,75270613 | Ik | IK cytokine |
| 17333854 | -2,651528861 | 0,005264931 | 0,174901557 | 0,615362746 | Ppp2r1a | protein phosphatase 2, regulatory subunit A, alpha |
| 17253972 | -2,6520956 | 0,005258452 | 0,174901557 | 0,623804796 | Psmd11 | proteasome (prosome, macropain) 26S subunit, non-ATPase, 11 |
| 17269368 | -2,653108381 | 0,0052442 | 0,174901557 | 0,566773446 | Krt19 | keratin 19 |
| 17352120 | -2,654372022 | 0,005231674 | 0,174901557 | 0,66554991 | Pard6g | par-6 family cell polarity regulator gamma |
| 17331438 | -2,654766189 | 0,005226491 | 0,174901557 | 0,769791633 | Chmp2b | charged multivesicular body protein 2B |
| 17353796 | -2,655642458 | 0,005218285 | 0,174901557 | 0,594846218 | Taf7 | TAF7 RNA polymerase II, TATA box binding protein (TBP)-associated factor |
| 17533746 | -2,65573428 | 0,005216126 | 0,174901557 | 0,723096003 | Agtr2 | angiotensin II receptor, type 2 |
| 17512833 | -2,664127643 | 0,005127585 | 0,174901557 | 0,73046813 | Ist1 | increased sodium tolerance 1 homolog (yeast) |
| 17262783 | -2,66644136 | 0,005102534 | 0,174901557 | 0,726446306 | Slc22a4 | solute carrier family 22 (organic cation transporter), member 4 |
| 17398043 | -2,667184195 | 0,005092169 | 0,174901557 | 0,692908997 | Vmn2r1 | vomeronasal 2, receptor 1 |
| 17531154 | -2,667203229 | 0,005091737 | 0,174901557 | 0,733496752 | Bsn | bassoon |
| 17479303 | -2,670901419 | 0,005051138 | 0,174834218 | 0,65753289 | Aen | apoptosis enhancing nuclease |
| 17338233 | -2,671984142 | 0,005038612 | 0,174834218 | 0,638633305 | Mrps10 | mitochondrial ribosomal protein S10 |
| 17514806 | -2,672162946 | 0,005036021 | 0,174834218 | 0,71156109 | Taf1d | TATA box binding protein (Tbp)-associated factor, RNA polymerase I, D |
| 17361032 | -2,672922179 | 0,005026087 | 0,174822971 | 0,792850817 | Ndufs8 | NADH dehydrogenase (ubiquinone) Fe-S protein 8 |
| 17250374 | -2,673237391 | 0,005023064 | 0,174822971 | 0,73845289 | Drg2 | developmentally regulated GTP binding protein 2 |
| 17507094 | -2,674917121 | 0,005005356 | 0,174653402 | 0,742652221 | Xab2 | XPA binding protein 2 |
| 17383284 | -2,675053376 | 0,005003196 | 0,174653402 | 0,828941042 | Vav2 | vav 2 oncogene |
| 17459614 | -2,675948716 | 0,004994126 | 0,174630374 | 0,757689235 | 0610030E20Rik | RIKEN cDNA 0610030E20 gene |
| 17517914 | -2,677056042 | 0,004982465 | 0,174407164 | 0,765272604 | Nptn | neuroplastin |
| 17529647 | -2,679319512 | 0,004958278 | 0,173744578 | 0,499188659 | Morf4l1 | mortality factor 4 like 1 |
| 17509682 | -2,682520629 | 0,004924589 | 0,17311482 | 0,52785366 | BC030870 | cDNA sequence BC030870 |
| 17373825 | -2,683266564 | 0,004916383 | 0,1730104 | 0,353978165 | Cd59a | CD59a antigen |
| 17276386 | -2,684230462 | 0,004906017 | 0,1730104 | 0,618383974 | Rhoj | ras homolog gene family, member J |
| 17281908 | -2,685225943 | 0,004898675 | 0,172939763 | 0,748004605 | Tmem30b | transmembrane protein 30B |
| 17359803 | -2,687621799 | 0,00487492 | 0,172469663 | 0,651471734 | Sfxn3 | sideroflexin 3 |
| 17335467 | -2,68834531 | 0,004867578 | 0,172394473 | 0,298237151 | Cdkn1a | cyclin-dependent kinase inhibitor 1A (P21) |
| 17227464 | -2,68942266 | 0,004858076 | 0,172242556 | 0,702641089 | 5730559C18Rik | RIKEN cDNA 5730559C18 gene |
| 17324762 | -2,691829135 | 0,004831298 | 0,171477128 | 0,724984675 | Rnf168 | ring finger protein 168 |
| 17294368 | -2,694329859 | 0,004803224 | 0,171216326 | 0,541978321 | Pdcd6 | programmed cell death 6 |
| 17386396 | -2,694769255 | 0,004799768 | 0,171216326 | 0,641300545 | Slc25a12 | solute carrier family 25 (mitochondrial carrier, Aralar), member 12 |
| 17376378 | -2,694987373 | 0,004797609 | 0,171216326 | 0,741134925 | Itpa | inosine triphosphatase (nucleoside triphosphate pyrophosphatase) |
| 17344794 | -2,697204468 | 0,004778605 | 0,171216326 | 0,463904845 | Znrd1 | zinc ribbon domain containing, 1 |
| 17357640 | -2,700784828 | 0,00474578 | 0,171216326 | 0,5234096 | Ms4a4a | membrane-spanning 4-domains, subfamily A, member 4A |
| 17506042 | -2,707981576 | 0,004678835 | 0,170836722 | 0,64113798 | Cdh13 | cadherin 13 |
| 17245383 | -2,713910773 | 0,004623551 | 0,169756037 | 0,637763452 | Helb | helicase (DNA) B |
| 17457343 | -2,714704844 | 0,004617936 | 0,169738486 | 0,560431806 | Atp6v0c | ATPase, H+ transporting, lysosomal V0 subunit C |
| 17291833 | -2,71495106 | 0,004615345 | 0,169738486 | 0,651694114 | Eci2 | enoyl-Coenzyme A delta isomerase 2 |
| 17414802 | -2,716110854 | 0,00460757 | 0,169738486 | 0,442857133 | Pappa | pregnancy-associated plasma protein A |
| 17346175 | -2,716445687 | 0,004603683 | 0,169738486 | 0,701397021 | Mydgf | myeloid derived growth factor |
| 17414416 | -2,718043851 | 0,004585975 | 0,169738486 | 0,760183774 | Dnajc25 | DnaJ (Hsp40) homolog, subfamily C, member 25 |
| 17526917 | -2,719138653 | 0,004576905 | 0,169738486 | 0,765064316 | 2310030G06Rik | RIKEN cDNA 2310030G06 gene |
| 17378628 | -2,72211682 | 0,004554014 | 0,169462457 | 0,663841499 | Aar2 | AAR2 splicing factor homolog (S. cerevisiae) |
| 17527977 | -2,725253598 | 0,004521189 | 0,169193657 | 0,630720734 | Glce | glucuronyl C5-epimerase |
| 17486110 | -2,731947434 | 0,004454676 | 0,168614124 | 0,550626816 | Peg3 | paternally expressed 3 |
| 17434310 | -2,733188407 | 0,004443446 | 0,168545619 | 0,556552899 | Fam133b | family with sequence similarity 133, member B |
| 17470187 | -2,733429711 | 0,004440423 | 0,168545619 | 0,68765518 | Zfp9 | zinc finger protein 9 |
| 17290083 | -2,734867464 | 0,004426602 | 0,168516657 | 0,74520962 | Emb | embigin |
| 17255387 | -2,735038979 | 0,004424874 | 0,168516657 | 0,69471919 | Slc35b1 | solute carrier family 35, member B1 |
| 17240199 | -2,735765254 | 0,004419691 | 0,168516657 | 0,614379542 | Nt5dc1 | 5'-nucleotidase domain containing 1 |
| 17306477 | -2,738227733 | 0,004393345 | 0,167830663 | 0,688707645 | Slc7a8 | solute carrier family 7 (cationic amino acid transporter, y+ system), member 8 |
| 17505774 | -2,740217957 | 0,004376069 | 0,167830663 | 0,725892034 | Gabarapl2 | gamma-aminobutyric acid (GABA) A receptor-associated protein-like 2 |
| 17309065 | -2,745347205 | 0,004332878 | 0,167619692 | 0,695111508 | Mzt1 | mitotic spindle organizing protein 1 |
| 17324274 | -2,745612561 | 0,004330287 | 0,167619692 | 0,642755047 | Senp2 | SUMO/sentrin specific peptidase 2 |
| 17397344 | -2,747310177 | 0,004314306 | 0,167522644 | 0,679516798 | Jade1 | jade family PHD finger 1 |
| 17541404 | -2,747977485 | 0,004309124 | 0,167518244 | 0,695152799 | Elf4 | E74-like factor 4 (ets domain transcription factor) |
| 17514832 | -2,755338322 | 0,004245202 | 0,166668489 | 0,632339155 | Taf1d | TATA box binding protein (Tbp)-associated factor, RNA polymerase I, D |
| 17339529 | -2,755541526 | 0,004243474 | 0,166668489 | 0,54056209 | Clip4 | CAP-GLY domain containing linker protein family, member 4 |
| 17425077 | -2,757718462 | 0,004221879 | 0,166668489 | 0,69635286 | Tbc1d2 | TBC1 domain family, member 2 |
| 17327331 | -2,757834385 | 0,004220151 | 0,166668489 | 0,621553797 | Chaf1b | chromatin assembly factor 1, subunit B (p60) |
| 17259794 | -2,763469001 | 0,00417005 | 0,166668489 | 0,53911254 | Rnf185 | ring finger protein 185 |
| 17376728 | -2,763519677 | 0,004168754 | 0,166668489 | 0,608161941 | Plcb4 | phospholipase C, beta 4 |
| 17468520 | -2,764192819 | 0,004160548 | 0,166668489 | 0,750245536 | Snrnp27 | small nuclear ribonucleoprotein 27 (U4/U6.U5) |
| 17303849 | -2,766514473 | 0,004145863 | 0,166668489 | 0,593515645 | Usp54 | ubiquitin specific peptidase 54 |
| 17337228 | -2,767751845 | 0,004136361 | 0,166668489 | 0,470759535 | Ier3 | immediate early response 3 |
| 17335383 | -2,769413109 | 0,004121245 | 0,166668489 | 0,569199605 | Mapk13 | mitogen-activated protein kinase 13 |
| 17479183 | -2,773217809 | 0,004086692 | 0,166668489 | 0,739552422 | Fam174b | family with sequence similarity 174, member B |
| 17308881 | -2,775050125 | 0,004072871 | 0,166668489 | 0,668594339 | Rgcc | regulator of cell cycle |
| 17246520 | -2,779696262 | 0,004041342 | 0,165684795 | 0,587135566 | Eif4enif1 | eukaryotic translation initiation factor 4E nuclear import factor 1 |
| 17313000 | -2,78126475 | 0,004027521 | 0,165684795 | 0,64154094 | Kdelr3 | KDEL (Lys-Asp-Glu-Leu) endoplasmic reticulum protein retention receptor 3 |
| 17523861 | -2,781305583 | 0,004027089 | 0,165684795 | 0,641725926 | C330006D17Rik | RIKEN cDNA C330006D17 gene |
| 17465942 | -2,781636575 | 0,004024498 | 0,165684795 | 0,411732126 | Atp6v0a4 | ATPase, H+ transporting, lysosomal V0 subunit A4 |
| 17550426 | -2,783900712 | 0,004008085 | 0,165553763 | 0,516733223 | Neat1 | nuclear paraspeckle assembly transcript 1 (non-protein coding) |
| 17390249 | -2,785898521 | 0,0039934 | 0,165225099 | 0,677831841 | Lrrc57 | leucine rich repeat containing 57 |
| 17364545 | -2,787841075 | 0,003976124 | 0,165058909 | 0,444027674 | Tctn3 | tectonic family member 3 |
| 17393357 | -2,795813643 | 0,003916089 | 0,163801393 | 0,616693759 | Eif6 | eukaryotic translation initiation factor 6 |
| 17458112 | -2,80083754 | 0,003871171 | 0,162953918 | 0,639585708 | Zfp398 | zinc finger protein 398 |
| 17315946 | -2,803407163 | 0,003851303 | 0,162739538 | 0,678459253 | Dnajc21 | DnaJ (Hsp40) homolog, subfamily C, member 21 |
| 17453205 | -2,807766855 | 0,003815455 | 0,162262231 | 0,658980052 | Sbds | Shwachman-Bodian-Diamond syndrome homolog (human) |
| 17420347 | -2,810183289 | 0,003797315 | 0,161864409 | 0,722690664 | Ece1 | endothelin converting enzyme 1 |
| 17211305 | -2,811517469 | 0,003789973 | 0,161803246 | 0,369164268 | Pi15 | peptidase inhibitor 15 |
| 17299869 | -2,822636784 | 0,003701864 | 0,159369283 | 0,581031863 | Trav9d-3 | T cell receptor alpha variable 9D-3 |
| 17431332 | -2,822778262 | 0,003699705 | 0,159369283 | 0,522164933 | Man1c1 | mannosidase, alpha, class 1C, member 1 |
| 17333465 | -2,827291114 | 0,003672063 | 0,159029476 | 0,475035242 | Smoc2 | SPARC related modular calcium binding 2 |
| 17363374 | -2,829136878 | 0,003658674 | 0,158657562 | 0,511484521 | Ostf1 | osteoclast stimulating factor 1 |
| 17288898 | -2,833041462 | 0,003633191 | 0,158209654 | 0,664284373 | Lysmd3 | LysM, putative peptidoglycan-binding, domain containing 3 |
| 17349181 | -2,835298323 | 0,003618938 | 0,158209654 | 0,662003169 | Wdr33 | WD repeat domain 33 |
| 17446775 | -2,836700914 | 0,003609868 | 0,158202043 | 0,659102403 | Preb | prolactin regulatory element binding |
| 17222527 | -2,837987894 | 0,003599071 | 0,158202043 | 0,428180789 | Tbc1d8 | TBC1 domain family, member 8 |
| 17312301 | -2,840091064 | 0,003583954 | 0,158114857 | 0,743693985 | Zfp707 | zinc finger protein 707 |
| 17532455 | -2,841218778 | 0,00357618 | 0,157982799 | 0,515684212 | 1110059G10Rik | RIKEN cDNA 1110059G10 gene |
| 17506697 | -2,842981118 | 0,003565814 | 0,157947197 | 0,415862105 | Rhou | ras homolog gene family, member U |
| 17277134 | -2,844512396 | 0,003554152 | 0,157871853 | 0,609387983 | Acot2 | acyl-CoA thioesterase 2 |
| 17364813 | -2,844907076 | 0,003550265 | 0,157871853 | 0,492644027 | Avpi1 | arginine vasopressin-induced 1 |
| 17402181 | -2,845262259 | 0,003548969 | 0,157871853 | 0,43002184 | F3 | coagulation factor III |
| 17453242 | -2,848236859 | 0,003530829 | 0,157871853 | 0,702887869 | Auts2 | autism susceptibility candidate 2 |
| 17459250 | -2,850424486 | 0,003516145 | 0,15780396 | 0,759080524 | Gng12 | guanine nucleotide binding protein (G protein), gamma 12 |
| 17309420 | -2,851785456 | 0,003509666 | 0,15780396 | 0,674755276 | Abcc4 | ATP-binding cassette, sub-family C (CFTR/MRP), member 4 |
| 17306333 | -2,853344362 | 0,003498005 | 0,15780396 | 0,634075011 | Dad1 | defender against cell death 1 |
| 17530406 | -2,85467586 | 0,003489366 | 0,15780396 | 0,673026503 | Acpp | acid phosphatase, prostate |
| 17396614 | -2,855539144 | 0,003485479 | 0,15780396 | 0,766754821 | Prkci | protein kinase C, iota |
| 17340232 | -2,856393878 | 0,00348116 | 0,15780396 | 0,604969375 | Cript | cysteine-rich PDZ-binding protein |
| 17257639 | -2,858179968 | 0,003469067 | 0,15780396 | 0,400220306 | 1810010H24Rik | RIKEN cDNA 1810010H24 gene |
| 17436791 | -2,860802131 | 0,003450063 | 0,15780396 | 0,588290921 | Mir3097 | microRNA 3097 |
| 17498199 | -2,861743951 | 0,003444016 | 0,15780396 | 0,685832791 | R74862 | expressed sequence R74862 |
| 17523994 | -2,863694605 | 0,003434083 | 0,15780396 | 0,686936842 | Cep57 | centrosomal protein 57 |
| 17334241 | -2,865718644 | 0,003414215 | 0,157568688 | 0,670905447 | Rnps1 | ribonucleic acid binding protein S1 |
| 17480312 | -2,86824249 | 0,003395211 | 0,157390409 | 0,570905384 | Gab2 | growth factor receptor bound protein 2-associated protein 2 |
| 17467197 | -2,870464458 | 0,003378799 | 0,157390409 | 0,723038751 | Vmn1r27 | vomeronasal 1 receptor 27 |
| 17306906 | -2,872336583 | 0,003366273 | 0,157333808 | 0,709115308 | Ripk3 | receptor-interacting serine-threonine kinase 3 |
| 17375083 | -2,876896935 | 0,003329129 | 0,156929906 | 0,581598741 | Snap23 | synaptosomal-associated protein 23 |
| 17243171 | -2,878067666 | 0,003319628 | 0,156929906 | 0,735257937 | Sgta | small glutamine-rich tetratricopeptide repeat (TPR)-containing, alpha |
| 17454794 | -2,878523691 | 0,003316604 | 0,156929906 | 0,593664009 | Rbak | RB-associated KRAB repressor |
| 17271968 | -2,879556918 | 0,003310126 | 0,156929906 | 0,72579217 | Mif4gd | MIF4G domain containing |
| 17221215 | -2,881478644 | 0,003295441 | 0,156929906 | 0,67872552 | Cops5 | COP9 (constitutive photomorphogenic) homolog, subunit 5 (Arabidopsis thaliana) |
| 17219643 | -2,884187716 | 0,003280324 | 0,156929906 | 0,786531027 | Aim2 | absent in melanoma 2 |
| 17478998 | -2,885880821 | 0,00327039 | 0,156929906 | 0,606187619 | Vimp | VCP-interacting membrane protein |
| 17286354 | -2,887874145 | 0,003255705 | 0,156929906 | 0,583372733 | Serpinb6b | serine (or cysteine) peptidase inhibitor, clade B, member 6b |
| 17478942 | -2,889643763 | 0,003246635 | 0,156929906 | 0,631472797 | Tm2d3 | TM2 domain containing 3 |
| 17516699 | -2,892183784 | 0,003232814 | 0,156927765 | 0,489560627 | Mpzl2 | myelin protein zero-like 2 |
| 17428054 | -2,89472776 | 0,003218562 | 0,156633315 | 0,649509314 | Gpx7 | glutathione peroxidase 7 |
| 17276139 | -2,89535071 | 0,003212083 | 0,156548588 | 0,573291672 | Dact1 | dapper homolog 1, antagonist of beta-catenin (xenopus) |
| 17399519 | -2,896452995 | 0,003205173 | 0,156548588 | 0,637401685 | Ube2q1 | ubiquitin-conjugating enzyme E2Q (putative) 1 |
| 17325093 | -2,898678324 | 0,00319092 | 0,156548588 | 0,647477629 | Muc13 | mucin 13, epithelial transmembrane |
| 17305221 | -2,901217807 | 0,003173643 | 0,156548588 | 0,556694248 | Fam213a | family with sequence similarity 213, member A |
| 17391137 | -2,902579033 | 0,003165437 | 0,156548588 | 0,627357297 | Usp50 | ubiquitin specific peptidase 50 |
| 17252170 | -2,902980154 | 0,003160686 | 0,156548588 | 0,564436041 | Rnf167 | ring finger protein 167 |
| 17338472 | -2,904412407 | 0,003150752 | 0,156548588 | 0,570003757 | Mocs1 | molybdenum cofactor synthesis 1 |
| 17379337 | -2,906410464 | 0,003136931 | 0,156548588 | 0,679466548 | Pabpc1l | poly(A) binding protein, cytoplasmic 1-like |
| 17278073 | -2,907920194 | 0,003126134 | 0,156548588 | 0,60756407 | Golga5 | golgi autoantigen, golgin subfamily a, 5 |
| 17432790 | -2,910318428 | 0,003115336 | 0,156548588 | 0,740855034 | Miip | migration and invasion inhibitory protein |
| 17476866 | -2,911514985 | 0,003109289 | 0,156548588 | 0,755984695 | Tshz3 | teashirt zinc finger family member 3 |
| 17501748 | -2,9169817 | 0,00307992 | 0,156115062 | 0,731911879 | Lpar2 | lysophosphatidic acid receptor 2 |
| 17511377 | -2,917357949 | 0,003076465 | 0,156115062 | 0,399082015 | Neto2 | neuropilin (NRP) and tolloid (TLL)-like 2 |
| 17212874 | -2,922889375 | 0,003047959 | 0,155667125 | 0,40034037 | Coq10b | coenzyme Q10 homolog B (S. cerevisiae) |
| 17434322 | -2,92346396 | 0,003043208 | 0,155665076 | 0,582549458 | NA | NA |
| 17262575 | -2,923977023 | 0,003039753 | 0,155665076 | 0,680468229 | Ube2b | ubiquitin-conjugating enzyme E2B |
| 17339460 | -2,924478915 | 0,003035434 | 0,155665076 | 0,390934376 | Lpin2 | lipin 2 |
| 17379300 | -2,926741977 | 0,003018589 | 0,155367822 | 0,716735444 | 0610039K10Rik | RIKEN cDNA 0610039K10 gene |
| 17285867 | -2,928953441 | 0,003006496 | 0,154986785 | 0,277871374 | Hist1h2ab | histone cluster 1, H2ab |
| 17278110 | -2,931061221 | 0,002994403 | 0,154986785 | 0,659746808 | Ubr7 | ubiquitin protein ligase E3 component n-recognin 7 (putative) |
| 17548498 | -2,934899053 | 0,002966761 | 0,154986785 | 0,471774487 | Gm10931 | predicted gene 10931 |
| 17548294 | -2,934899053 | 0,002966761 | 0,154986785 | 0,471774487 | Gm10931 | predicted gene 10931 |
| 17520560 | -2,934915756 | 0,002965897 | 0,154986785 | 0,620678896 | Tfdp2 | transcription factor Dp 2 |
| 17418413 | -2,936511481 | 0,002960714 | 0,154986785 | 0,706558314 | Gnl2 | guanine nucleotide binding protein-like 2 (nucleolar) |
| 17340177 | -2,93854291 | 0,002945165 | 0,154986785 | 0,627239193 | Prkce | protein kinase C, epsilon |
| 17300251 | -2,940000283 | 0,002937823 | 0,154986785 | 0,551183972 | Abhd4 | abhydrolase domain containing 4 |
| 17452209 | -2,940104699 | 0,002936527 | 0,154986785 | 0,813882741 | Mapkapk5 | MAP kinase-activated protein kinase 5 |
| 17213580 | -2,944413182 | 0,002916228 | 0,154676898 | 0,764208062 | Eef1b2 | eukaryotic translation elongation factor 1 beta 2 |
| 17468705 | -2,948954722 | 0,002889881 | 0,154554536 | 0,649242507 | Rab7 | RAB7, member RAS oncogene family |
| 17464638 | -2,955205287 | 0,002854465 | 0,154499461 | 0,660399108 | Pon2 | paraoxonase 2 |
| 17465924 | -2,961750978 | 0,002821208 | 0,153604887 | 0,758205304 | Svopl | SV2 related protein homolog (rat)-like |
| 17535344 | -2,962965749 | 0,002810843 | 0,153604887 | 0,74143475 | Hmgb3 | high mobility group box 3 |
| 17316057 | -2,963196376 | 0,002809979 | 0,153604887 | 0,699046058 | Sub1 | SUB1 homolog (S. cerevisiae) |
| 17522430 | -2,963305896 | 0,002808683 | 0,153604887 | 0,698891063 | Ccdc12 | coiled-coil domain containing 12 |
| 17531265 | -2,964906188 | 0,002798317 | 0,153604887 | 0,661295314 | Atrip | ATR interacting protein |
| 17532045 | -2,966402709 | 0,00278536 | 0,153604887 | 0,503012573 | Plcd1 | phospholipase C, delta 1 |
| 17443275 | -2,974483855 | 0,002743033 | 0,153133373 | 0,598569656 | Rhbdd2 | rhomboid domain containing 2 |
| 17331642 | -2,975078079 | 0,002739578 | 0,153133373 | 0,798131229 | Mrpl39 | mitochondrial ribosomal protein L39 |
| 17443972 | -2,976104793 | 0,0027331 | 0,153133373 | 0,690352306 | Get4 | golgi to ER traffic protein 4 homolog (S. cerevisiae) |
| 17234667 | -2,979301423 | 0,002721006 | 0,153133373 | 0,437514462 | Pttg1ip | pituitary tumor-transforming 1 interacting protein |
| 17289324 | -2,982846927 | 0,002705026 | 0,152916443 | 0,703496759 | Gcnt4 | glucosaminyl (N-acetyl) transferase 4, core 2 (beta-1,6-N-acetylglucosaminyltransferase) |
| 17241409 | -2,983090787 | 0,002704162 | 0,152916443 | 0,653022292 | Srgn | serglycin |
| 17302061 | -2,986356803 | 0,002689045 | 0,152916443 | 0,544488517 | Nufip1 | nuclear fragile X mental retardation protein interacting protein 1 |
| 17380185 | -2,989257438 | 0,002678679 | 0,152874224 | 0,668366125 | Rae1 | RAE1 RNA export 1 homolog (S. pombe) |
| 17462395 | -2,991552339 | 0,002662267 | 0,152728896 | 0,429969007 | Slc25a18 | solute carrier family 25 (mitochondrial carrier), member 18 |
| 17292689 | -2,992155273 | 0,00265838 | 0,152728896 | 0,424572015 | Thoc3 | THO complex 3 |
| 17303804 | -2,992202705 | 0,002657516 | 0,152728896 | 0,701389748 | Anxa7 | annexin A7 |
| 17301697 | -2,992249601 | 0,002657084 | 0,152728896 | 0,469105204 | Tnfrsf10b | tumor necrosis factor receptor superfamily, member 10b |
| 17439769 | -3,003098324 | 0,002600073 | 0,152587611 | 0,677476493 | Nudt9 | nudix (nucleoside diphosphate linked moiety X)-type motif 9 |
| 17507623 | -3,009902282 | 0,002563361 | 0,151256401 | 0,724972922 | 1700029H14Rik | RIKEN cDNA 1700029H14 gene |
| 17306532 | -3,010216875 | 0,002560337 | 0,151256401 | 0,693651303 | Myh6 | myosin, heavy polypeptide 6, cardiac muscle, alpha |
| 17321467 | -3,010538183 | 0,002558178 | 0,151256401 | 0,578520813 | Tuba1b | tubulin, alpha 1B |
| 17451661 | -3,010792679 | 0,002556882 | 0,151256401 | 0,756188227 | Unc119b | unc-119 homolog B (C. elegans) |
| 17312295 | -3,012227814 | 0,00254954 | 0,151256401 | 0,742327979 | Zfp623 | zinc finger protein 623 |
| 17502049 | -3,016290912 | 0,002528376 | 0,151256401 | 0,579175341 | 2010320M18Rik | RIKEN cDNA 2010320M18 gene |
| 17238580 | -3,022007492 | 0,002507213 | 0,151256401 | 0,715589074 | Sarnp | SAP domain containing ribonucleoprotein |
| 17430930 | -3,037504717 | 0,002432061 | 0,150014459 | 0,460384221 | Stx12 | syntaxin 12 |
| 17453347 | -3,041525609 | 0,002418672 | 0,149667614 | 0,652307123 | Gtf2ird1 | general transcription factor II I repeat domain-containing 1 |
| 17469572 | -3,044939539 | 0,002406147 | 0,149171898 | 0,615780636 | Sumf1 | sulfatase modifying factor 1 |
| 17279230 | -3,04524379 | 0,002403555 | 0,149171898 | 0,594642292 | Zfyve21 | zinc finger, FYVE domain containing 21 |
| 17508968 | -3,050021389 | 0,002377209 | 0,149171898 | 0,701068037 | Frg1 | FSHD region gene 1 |
| 17241032 | -3,050089073 | 0,002376777 | 0,149171898 | 0,484360198 | Ddit4 | DNA-damage-inducible transcript 4 |
| 17458641 | -3,051693865 | 0,002368139 | 0,149171898 | 0,652911438 | Tax1bp1 | Tax1 (human T cell leukemia virus type I) binding protein 1 |
| 17469775 | -3,052307919 | 0,002364684 | 0,149171898 | 0,672987356 | Emc3 | ER membrane protein complex subunit 3 |
| 17377177 | -3,053117132 | 0,002361661 | 0,149171898 | 0,667126324 | Rin2 | Ras and Rab interactor 2 |
| 17341080 | -3,056224366 | 0,002348272 | 0,149171898 | 0,521115657 | Thbs2 | thrombospondin 2 |
| 17363407 | -3,060562651 | 0,002328404 | 0,148532208 | 0,600757088 | Anxa1 | annexin A1 |
| 17321078 | -3,062682627 | 0,002317606 | 0,148129375 | 0,439200746 | Vdr | vitamin D receptor |
| 17419636 | -3,067492111 | 0,002296011 | 0,147319016 | 0,574142474 | Gpn2 | GPN-loop GTPase 2 |
| 17263482 | -3,068086507 | 0,002293419 | 0,147319016 | 0,583091296 | Cops3 | COP9 (constitutive photomorphogenic) homolog, subunit 3 (Arabidopsis thaliana) |
| 17534142 | -3,068739652 | 0,002290396 | 0,147319016 | 0,607534488 | Nkap | NFKB activating protein |
| 17432927 | -3,07491865 | 0,002264914 | 0,147319016 | 0,741739853 | Fbxo44 | F-box protein 44 |
| 17493875 | -3,077615058 | 0,002255412 | 0,147319016 | 0,411239006 | P2ry2 | purinergic receptor P2Y, G-protein coupled 2 |
| **17493556** | **-3,07894926** | **0,002248933** | **0,147319016** | **0,568002505** | **Acer3** | **alkaline ceramidase 3** |
| 17319124 | -3,080983727 | 0,002241159 | 0,147319016 | 0,580846043 | 1700088E04Rik | RIKEN cDNA 1700088E04 gene |
| 17417791 | -3,084599703 | 0,002228202 | 0,147257217 | 0,684555165 | Ebna1bp2 | EBNA1 binding protein 2 |
| 17222072 | -3,086012436 | 0,002220427 | 0,147151909 | 0,436229852 | Ccdc115 | coiled-coil domain containing 115 |
| 17349634 | -3,091004395 | 0,002192354 | 0,14614286 | 0,724890267 | Cystm1 | cysteine-rich transmembrane module containing 1 |
| 17550492 | -3,091240502 | 0,002190194 | 0,14614286 | 0,678462934 | Gm11974 | predicted gene 11974 |
| 17444494 | -3,093731945 | 0,002177669 | 0,145961053 | 0,692864083 | Gm15708 | predicted gene 15708 |
| 17229166 | -3,095001162 | 0,002169463 | 0,145706572 | 0,678132114 | Blzf1 | basic leucine zipper nuclear factor 1 |
| 17321169 | -3,10532825 | 0,002126704 | 0,145228743 | 0,727657558 | Asb8 | ankyrin repeat and SOCS box-containing 8 |
| 17504281 | -3,108913693 | 0,002112451 | 0,14482105 | 0,712022873 | Usb1 | U6 snRNA biogenesis 1 |
| 17515758 | -3,11068183 | 0,002102949 | 0,14482105 | 0,692146553 | Nfrkb | nuclear factor related to kappa B binding protein |
| 17240819 | -3,111093357 | 0,002099926 | 0,14482105 | 0,448957228 | Cep85l | centrosomal protein 85-like |
| 17434127 | -3,113606737 | 0,002087832 | 0,144753326 | 0,49649057 | Gm10590 | predicted gene 10590 |
| 17424410 | -3,113606737 | 0,002087832 | 0,144753326 | 0,49649057 | Gm10590 | predicted gene 10590 |
| 17412998 | -3,113606737 | 0,002087832 | 0,144753326 | 0,49649057 | Gm10590 | predicted gene 10590 |
| 17266489 | -3,12058551 | 0,002056303 | 0,144230612 | 0,71972203 | Tmem97 | transmembrane protein 97 |
| 17287361 | -3,126396885 | 0,002040323 | 0,144230612 | 0,550516669 | Gadd45g | growth arrest and DNA-damage-inducible 45 gamma |
| 17377199 | -3,133163771 | 0,002018727 | 0,143764545 | 0,438551428 | Naa20 | N(alpha)-acetyltransferase 20, NatB catalytic subunit |
| 17468195 | -3,133350954 | 0,002017 | 0,143764545 | 0,578877605 | Stambp | STAM binding protein |
| 17443310 | -3,133687298 | 0,002013976 | 0,143764545 | 0,690897214 | Mdh2 | malate dehydrogenase 2, NAD (mitochondrial) |
| 17463441 | -3,135628498 | 0,002009657 | 0,143764545 | 0,612454214 | Tom1 | target of myb1 homolog (chicken) |
| 17451431 | -3,136261558 | 0,002005338 | 0,143764545 | 0,663245348 | Tmem119 | transmembrane protein 119 |
| 17407647 | -3,137383353 | 0,001999292 | 0,143764545 | 0,603920306 | A730011C13Rik | RIKEN cDNA A730011C13 gene |
| 17318461 | -3,137775359 | 0,001998428 | 0,143764545 | 0,74149168 | Sharpin | SHANK-associated RH domain interacting protein |
| 17277592 | -3,140056379 | 0,001991949 | 0,143764545 | 0,691264997 | Gstz1 | glutathione transferase zeta 1 (maleylacetoacetate isomerase) |
| 17529076 | -3,140753941 | 0,001989358 | 0,143764545 | 0,54757579 | Slc17a5 | solute carrier family 17 (anion/sugar transporter), member 5 |
| 17403025 | -3,142604954 | 0,001981584 | 0,143764545 | 0,706140824 | Lamtor3 | late endosomal/lysosomal adaptor, MAPK and MTOR activator 3 |
| 17319806 | -3,142624914 | 0,001981152 | 0,143764545 | 0,521405946 | Arfgap3 | ADP-ribosylation factor GTPase activating protein 3 |
| 17440757 | -3,143930082 | 0,001975537 | 0,143764545 | 0,565081764 | Iscu | IscU iron-sulfur cluster scaffold homolog (E. coli) |
| 17399672 | -3,145056898 | 0,001967331 | 0,143764545 | 0,676977068 | Slc39a1 | solute carrier family 39 (zinc transporter), member 1 |
| 17538356 | -3,145381723 | 0,001966467 | 0,143764545 | 0,639182508 | Alg13 | asparagine-linked glycosylation 13 |
| 17535752 | -3,147256115 | 0,001957829 | 0,143764545 | 0,596749711 | Emd | emerin |
| 17429234 | -3,15125999 | 0,00194444 | 0,143764545 | 0,718142259 | Tmem125 | transmembrane protein 125 |
| 17380134 | -3,151436008 | 0,001942712 | 0,143764545 | 0,62916817 | Rtfdc1 | replication termination factor 2 domain containing 1 |
| 17378784 | -3,151646776 | 0,001941416 | 0,143764545 | 0,576910766 | Rprd1b | regulation of nuclear pre-mRNA domain containing 1B |
| 17496887 | -3,152888829 | 0,001938393 | 0,143764545 | 0,535242817 | Rgs10 | regulator of G-protein signalling 10 |
| 17542149 | -3,15459343 | 0,001930619 | 0,143764545 | 0,672379998 | Ids | iduronate 2-sulfatase |
| 17287726 | -3,157364859 | 0,001923276 | 0,143764545 | 0,705374963 | Caml | calcium modulating ligand |
| 17482366 | -3,15840746 | 0,001918525 | 0,143764545 | 0,396400538 | Tmem159 | transmembrane protein 159 |
| 17291570 | -3,162333663 | 0,001905568 | 0,143764545 | 0,673320278 | Uqcrfs1 | ubiquinol-cytochrome c reductase, Rieske iron-sulfur polypeptide 1 |
| 17502860 | -3,164106453 | 0,001898226 | 0,143764545 | 0,697755613 | Rnf150 | ring finger protein 150 |
| 17460918 | -3,164145273 | 0,001897794 | 0,143764545 | 0,507691572 | Tmem43 | transmembrane protein 43 |
| 17470627 | -3,176124458 | 0,001861082 | 0,143764545 | 0,583904837 | Clec4e | C-type lectin domain family 4, member e |
| 17355113 | -3,17935585 | 0,001848988 | 0,143764545 | 0,591547038 | Ccbe1 | collagen and calcium binding EGF domains 1 |
| 17541008 | -3,180767614 | 0,001842942 | 0,143764545 | 0,417776766 | Snora69 | small nucleolar RNA, H/ACA box 69 |
| 17498245 | -3,183987927 | 0,001833872 | 0,143764545 | 0,713236797 | Cdkn1c | cyclin-dependent kinase inhibitor 1C (P57) |
| 17495097 | -3,188406806 | 0,001823506 | 0,143764545 | 0,496090994 | Lyve1 | lymphatic vessel endothelial hyaluronan receptor 1 |
| 17289699 | -3,193602942 | 0,001803206 | 0,143764545 | 0,715513419 | Ercc8 | excision repaiross-complementing rodent repair deficiency, complementation group 8 |
| 17422817 | -3,203150732 | 0,00177686 | 0,143764545 | 0,594126066 | Ube2j2 | ubiquitin-conjugating enzyme E2J 2 |
| 17514826 | -3,209674254 | 0,001759152 | 0,143764545 | 0,495265611 | Taf1d | TATA box binding protein (Tbp)-associated factor, RNA polymerase I, D |
| 17447831 | -3,209859744 | 0,001758288 | 0,143764545 | 0,572845219 | Fgfbp1 | fibroblast growth factor binding protein 1 |
| 17481960 | -3,212437555 | 0,001748786 | 0,143764545 | 0,684533511 | Arntl | aryl hydrocarbon receptor nuclear translocator-like |
| 17545459 | -3,216846927 | 0,001737989 | 0,143764545 | 0,720029811 | Shroom2 | shroom family member 2 |
| 17444674 | -3,219806088 | 0,001725463 | 0,143764545 | 0,71364088 | Zscan25 | zinc finger and SCAN domain containing 25 |
| 17413158 | -3,227961342 | 0,001693934 | 0,143764545 | 0,559648697 | Gm10590 | predicted gene 10590 |
| 17526206 | -3,228581149 | 0,001691775 | 0,143764545 | 0,709094726 | C2cd2l | C2 calcium-dependent domain containing 2-like |
| 17368834 | -3,230843617 | 0,001680113 | 0,143764545 | 0,607217719 | Med27 | mediator complex subunit 27 |
| 17453703 | -3,236061044 | 0,001661109 | 0,143764545 | 0,74253504 | Prkrip1 | Prkr interacting protein 1 (IL11 inducible) |
| 17505967 | -3,238036806 | 0,001654631 | 0,143764545 | 0,750756228 | Cmip | c-Maf inducing protein |
| 17396492 | -3,242207228 | 0,001647288 | 0,143764545 | 0,617044583 | Eif5a2 | eukaryotic translation initiation factor 5A2 |
| 17284037 | -3,248485737 | 0,001630444 | 0,143764545 | 0,434752184 | Mpc1 | mitochondrial pyruvate carrier 1 |
| 17285863 | -3,266749083 | 0,001582071 | 0,141291573 | 0,690778604 | Hist1h2bb | histone cluster 1, H2bb |
| 17368079 | -3,26948524 | 0,001576024 | 0,141291573 | 0,696214241 | Fbxw5 | F-box and WD-40 domain protein 5 |
| 17440086 | -3,272009537 | 0,001566954 | 0,141085467 | 0,65213089 | Rpap2 | RNA polymerase II associated protein 2 |
| 17431302 | -3,273111252 | 0,001563931 | 0,141085467 | 0,622978707 | Mtfr1l | mitochondrial fission regulator 1-like |
| 17411054 | -3,280888731 | 0,001546223 | 0,141085467 | 0,686706455 | Rpf1 | ribosome production factor 1 homolog (S. cerevisiae) |
| 17264153 | -3,284077773 | 0,001534561 | 0,141085467 | 0,482531323 | 1700086D15Rik | RIKEN cDNA 1700086D15 gene |
| 17374686 | -3,285053878 | 0,001531106 | 0,141085467 | 0,59096413 | Rpusd2 | RNA pseudouridylate synthase domain containing 2 |
| **sphk** | **-3,289287203** | **0,001520308** | **0,141085467** | **0,699798841** | **Neu3** | **neuraminidase 3** |
| 17402296 | -3,292596748 | 0,001512534 | 0,14107046 | 0,724573078 | Dnttip2 | deoxynucleotidyltransferase, terminal, interacting protein 2 |
| 17465608 | -3,295216588 | 0,001508647 | 0,14107046 | 0,508548194 | Lincpint | long intergenic non-protein coding RNA, Trp53 induced transcript |
| 17272519 | -3,300420245 | 0,001494826 | 0,14107046 | 0,697920319 | Jmjd6 | jumonji domain containing 6 |
| 17256565 | -3,302589284 | 0,001487483 | 0,14107046 | 0,637032736 | Tubg2 | tubulin, gamma 2 |
| 17231574 | -3,309663144 | 0,001467616 | 0,140567638 | 0,684961856 | Ppil4 | peptidylprolyl isomerase (cyclophilin)-like 4 |
| 17240357 | -3,309953408 | 0,001466752 | 0,140567638 | 0,630537095 | Gtf3c6 | general transcription factor IIIC, polypeptide 6, alpha |
| 17298775 | -3,315862757 | 0,001451203 | 0,139806133 | 0,716051526 | Anxa8 | annexin A8 |
| 17455093 | -3,318774053 | 0,001443429 | 0,139806133 | 0,766056229 | Zkscan14 | zinc finger with KRAB and SCAN domains 14 |
| 17308842 | -3,320097347 | 0,001439974 | 0,139806133 | 0,461844373 | Dgkh | diacylglycerol kinase, eta |
| 17308233 | -3,321573275 | 0,00143695 | 0,139806133 | 0,491003722 | 9930012K11Rik | RIKEN cDNA 9930012K11 gene |
| 17500068 | -3,323521437 | 0,001433927 | 0,139806133 | 0,476706938 | Sfrp1 | secreted frizzled-related protein 1 |
| 17374594 | -3,324472957 | 0,001429608 | 0,139806133 | 0,466066542 | Pak6 | p21 protein (Cdc42/Rac)-activated kinase 6 |
| 17375327 | -3,331346212 | 0,001416219 | 0,139806133 | 0,594416505 | Casc4 | cancer susceptibility candidate 4 |
| 17220974 | -3,331383696 | 0,001415787 | 0,139806133 | 0,559654837 | Plxna2 | plexin A2 |
| 17241637 | -3,335331226 | 0,001406285 | 0,139806133 | 0,620285776 | Nrbf2 | nuclear receptor binding factor 2 |
| 17234192 | -3,338915233 | 0,001395487 | 0,139806133 | 0,538660813 | Zwint | ZW10 interactor |
| 17214825 | -3,341423389 | 0,001387281 | 0,139806133 | 0,660268405 | Mff | mitochondrial fission factor |
| 17473269 | -3,344812939 | 0,001378643 | 0,139806133 | 0,760651781 | Leng8 | leukocyte receptor cluster (LRC) member 8 |
| 17313016 | -3,345156374 | 0,001377779 | 0,139806133 | 0,62691808 | Tomm22 | translocase of outer mitochondrial membrane 22 homolog (yeast) |
| 17258457 | -3,345165902 | 0,001377347 | 0,139806133 | 0,754312856 | Sap30bp | SAP30 binding protein |
| 17332851 | -3,346145344 | 0,001373892 | 0,139806133 | 0,695521805 | Gtf2h5 | general transcription factor IIH, polypeptide 5 |
| 17320947 | -3,347039758 | 0,001370005 | 0,139806133 | 0,362732325 | Slc38a4 | solute carrier family 38, member 4 |
| 17366437 | -3,347143691 | 0,001369141 | 0,139806133 | 0,516745636 | Fam107b | family with sequence similarity 107, member B |
| 17506917 | -3,348136414 | 0,001366982 | 0,139806133 | 0,326006331 | Kcnk1 | potassium channel, subfamily K, member 1 |
| 17292157 | -3,350273602 | 0,001360071 | 0,139806133 | 0,620463742 | Dtnbp1 | dystrobrevin binding protein 1 |
| 17220018 | -3,357718819 | 0,001340635 | 0,139806133 | 0,577888053 | Cnst | consortin, connexin sorting protein |
| 17362320 | -3,359977929 | 0,001333293 | 0,139806133 | 0,667915644 | AI846148 | expressed sequence AI846148 |
| 17512740 | -3,367262041 | 0,001315153 | 0,139806133 | 0,590327769 | Nob1 | NIN1/RPN12 binding protein 1 homolog (S. cerevisiae) |
| 17487952 | -3,374525545 | 0,001294853 | 0,139806133 | 0,481109546 | Ceacam1 | carcinoembryonic antigen-related cell adhesion molecule 1 |
| 17503377 | -3,374898042 | 0,001293558 | 0,139806133 | 0,689560337 | Dhps | deoxyhypusine synthase |
| 17386135 | -3,377816634 | 0,001286215 | 0,139806133 | 0,683866066 | Stk39 | serine/threonine kinase 39 |
| 17242773 | -3,38173854 | 0,001279305 | 0,139806133 | 0,476948811 | Polr2e | polymerase (RNA) II (DNA directed) polypeptide E |
| 17444828 | -3,3927032 | 0,001252095 | 0,139806133 | 0,56651793 | Polr1d | polymerase (RNA) I polypeptide D |
| 17381283 | -3,396356237 | 0,001243025 | 0,139806133 | 0,557166499 | Prpf18 | PRP18 pre-mRNA processing factor 18 homolog (yeast) |
| 17488001 | -3,39924399 | 0,001237842 | 0,139806133 | 0,740683411 | Ccdc97 | coiled-coil domain containing 97 |
| 17512732 | -3,40372337 | 0,001229636 | 0,139806133 | 0,578431755 | Nqo1 | NAD(P)H dehydrogenase, quinone 1 |
| 17270724 | -3,408107144 | 0,001224021 | 0,139806133 | 0,732808744 | Taco1os | translational activator of mitochondrially encoded cytochrome c oxidase I, opposite strand |
| 17467711 | -3,41031971 | 0,001218838 | 0,139806133 | 0,593266468 | Usp39 | ubiquitin specific peptidase 39 |
| 17253885 | -3,432870782 | 0,001164418 | 0,139806133 | 0,545319177 | Adap2 | ArfGAP with dual PH domains 2 |
| 17311821 | -3,449762905 | 0,001132457 | 0,139806133 | 0,671602404 | Nsmce2 | non-SMC element 2 homolog (MMS21, S. cerevisiae) |
| 17301440 | -3,453867795 | 0,001122091 | 0,139806133 | 0,489198594 | Ccdc25 | coiled-coil domain containing 25 |
| 17515062 | -3,47139927 | 0,001084947 | 0,139806133 | 0,665794484 | Mrpl4 | mitochondrial ribosomal protein L4 |
| 17506754 | -3,473541247 | 0,00108106 | 0,139806133 | 0,544532568 | Cog2 | component of oligomeric golgi complex 2 |
| 17442588 | -3,473818467 | 0,001080196 | 0,139806133 | 0,524454143 | Atp6v0a2 | ATPase, H+ transporting, lysosomal V0 subunit A2 |
| 17258341 | -3,476527556 | 0,00107415 | 0,139806133 | 0,712731984 | Llgl2 | lethal giant larvae homolog 2 (Drosophila) |
| 17236800 | -3,479401442 | 0,001068535 | 0,139806133 | 0,496946546 | Dcn | decorin |
| 17289289 | -3,497048136 | 0,001042189 | 0,139806133 | 0,69446935 | Poc5 | POC5 centriolar protein homolog (Chlamydomonas) |
| 17463702 | -3,504309212 | 0,001027072 | 0,139806133 | 0,734493683 | Loh12cr1 | loss of heterozygosity, 12, chromosomal region 1 homolog (human) |
| 17358007 | -3,510314282 | 0,001016706 | 0,139806133 | 0,621860904 | Rfk | riboflavin kinase |
| 17482681 | -3,514125786 | 0,00100634 | 0,139806133 | 0,631720937 | Scnn1g | sodium channel, nonvoltage-gated 1 gamma |
| 17401169 | -3,520312453 | 0,000995975 | 0,139806133 | 0,59957355 | Bcas2 | breast carcinoma amplified sequence 2 |
| 17275718 | -3,556639111 | 0,000943714 | 0,139806133 | 0,627317635 | Mia2 | melanoma inhibitory activity 2 |
| 17327909 | -3,559964149 | 0,000937667 | 0,139806133 | 0,510715901 | Ppl | periplakin |
| 17468511 | -3,565659476 | 0,000928165 | 0,139806133 | 0,660609484 | Mxd1 | MAX dimerization protein 1 |
| 17249461 | -3,56980662 | 0,000919527 | 0,139806133 | 0,627153803 | Fstl4 | follistatin-like 4 |
| 17408099 | -3,569884607 | 0,000919095 | 0,139806133 | 0,73518066 | Polr3c | polymerase (RNA) III (DNA directed) polypeptide C |
| 17411732 | -3,57430591 | 0,000913049 | 0,139806133 | 0,697540976 | 2610301B20Rik | RIKEN cDNA 2610301B20 gene |
| 17340397 | -3,577868578 | 0,000905706 | 0,139806133 | 0,533152124 | Nanp | N-acetylneuraminic acid phosphatase |
| 17354378 | -3,586120674 | 0,000890158 | 0,139806133 | 0,677136421 | Ppic | peptidylprolyl isomerase C |
| 17383104 | -3,58950033 | 0,000884543 | 0,139806133 | 0,719562327 | Agpat2 | 1-acylglycerol-3-phosphate O-acyltransferase 2 (lysophosphatidic acid acyltransferase, beta) |
| 17363605 | -3,594098108 | 0,000876769 | 0,139806133 | 0,519354267 | Pip5k1b | phosphatidylinositol-4-phosphate 5-kinase, type 1 beta |
| 17548850 | -3,594787251 | 0,000875473 | 0,139806133 | 0,578479595 | Arpc1b | actin related protein 2/3 complex, subunit 1B |
| 17389009 | -3,598350423 | 0,000872018 | 0,139806133 | 0,655885569 | Eif3m | eukaryotic translation initiation factor 3, subunit M |
| **17230595** | **-3,600908628** | **0,000866835** | **0,139806133** | **0,672541857** | **Degs1** | **degenerative spermatocyte homolog 1 (Drosophila)** |
| 17518585 | -3,6041549 | 0,00086122 | 0,139806133 | 0,492572435 | Spg21 | spastic paraplegia 21 homolog (human) |
| 17254537 | -3,604548687 | 0,000860356 | 0,139806133 | 0,688280503 | Ppm1d | protein phosphatase 1D magnesium-dependent, delta isoform |
| 17422612 | -3,606694512 | 0,000858629 | 0,139806133 | 0,546327082 | Slc35e2 | solute carrier family 35, member E2 |
| 17334685 | -3,615074037 | 0,000847831 | 0,139806133 | 0,745631637 | Tekt4 | tektin 4 |
| 17461606 | -3,64627147 | 0,000806368 | 0,138060066 | 0,671870433 | Brpf1 | bromodomain and PHD finger containing, 1 |
| 17550428 | -3,647607329 | 0,000803345 | 0,138060066 | 0,32956441 | Neat1 | nuclear paraspeckle assembly transcript 1 (non-protein coding) |
| 17499922 | -3,661889709 | 0,000783909 | 0,137055318 | 0,57111072 | Mrps31 | mitochondrial ribosomal protein S31 |
| 17324305 | -3,69670445 | 0,000738991 | 0,136174199 | 0,71908677 | Dnajb11 | DnaJ (Hsp40) homolog, subfamily B, member 11 |
| 17516921 | -3,726462048 | 0,00070487 | 0,134634114 | 0,712324047 | Zpr1 | ZPR1 zinc finger |
| 17419222 | -3,733573785 | 0,000694936 | 0,134289173 | 0,739502152 | Snrnp40 | small nuclear ribonucleoprotein 40 (U5) |
| 17276732 | -3,740144462 | 0,000689322 | 0,134289173 | 0,573178085 | Eif2s1 | eukaryotic translation initiation factor 2, subunit 1 alpha |
| 17462373 | -3,757901224 | 0,000670318 | 0,133061743 | 0,441843787 | Cecr2 | cat eye syndrome chromosome region, candidate 2 |
| 17509171 | -3,771567859 | 0,000654337 | 0,132737203 | 0,676551639 | Slc25a4 | solute carrier family 25 (mitochondrial carrier, adenine nucleotide translocator), member 4 |
| 17286254 | -3,797324471 | 0,000628855 | 0,132737203 | 0,48272016 | Mboat1 | membrane bound O-acyltransferase domain containing 1 |
| 17223313 | -3,797704456 | 0,000627991 | 0,132737203 | 0,672558507 | Tyw5 | tRNA-yW synthesizing protein 5 |
| 17428885 | -3,80137802 | 0,000624968 | 0,132737203 | 0,706298653 | Dmap1 | DNA methyltransferase 1-associated protein 1 |
| 17497076 | -3,806519272 | 0,000619353 | 0,132737203 | 0,690577859 | Chst15 | carbohydrate (N-acetylgalactosamine 4-sulfate 6-O) sulfotransferase 15 |
| 17231859 | -3,807188167 | 0,000618921 | 0,132737203 | 0,567789061 | Ifngr1 | interferon gamma receptor 1 |
| 17356099 | -3,83422815 | 0,000594734 | 0,132737203 | 0,618854199 | Cdk2ap2 | CDK2-associated protein 2 |
| 17479548 | -3,839799454 | 0,000590847 | 0,132737203 | 0,495682389 | Vps33b | vacuolar protein sorting 33B (yeast) |
| 17342042 | -3,846845987 | 0,000585232 | 0,132737203 | 0,578845895 | Nubp2 | nucleotide binding protein 2 |
| 17266185 | -3,87471105 | 0,000564501 | 0,132737203 | 0,705566022 | Ccdc55 | coiled-coil domain containing 55 |
| 17464503 | -3,877044124 | 0,000563637 | 0,132737203 | 0,727517162 | 2810474O19Rik | RIKEN cDNA 2810474O19 gene |
| 17427155 | -3,881256759 | 0,000560614 | 0,132737203 | 0,515698326 | Cdkn2b | cyclin-dependent kinase inhibitor 2B (p15, inhibits CDK4) |
| 17329454 | -3,943569936 | 0,000508353 | 0,132267702 | 0,671211642 | P3h2 | prolyl 3-hydroxylase 2 |
| 17548440 | -3,947796389 | 0,000503602 | 0,132184757 | 0,685442738 | Imp3 | IMP3, U3 small nucleolar ribonucleoprotein, homolog (yeast) |
| 17524958 | -3,9559556 | 0,000498419 | 0,132184757 | 0,610775772 | Zfp810 | zinc finger protein 810 |
| 17276864 | -3,960284316 | 0,000494964 | 0,132184757 | 0,710433769 | Plekhd1 | pleckstrin homology domain containing, family D (with coiled-coil domains) member 1 |
| 17503431 | -3,962058249 | 0,000493668 | 0,132184757 | 0,559190937 | Orc6 | origin recognition complex, subunit 6 |
| 17211998 | -3,971919205 | 0,000483734 | 0,132184757 | 0,714505212 | Mrpl30 | mitochondrial ribosomal protein L30 |
| 17519967 | -3,973869516 | 0,000482007 | 0,132184757 | 0,711876703 | Tpbg | trophoblast glycoprotein |
| 17384619 | -3,982798977 | 0,000473801 | 0,132184757 | 0,444753712 | Snord90 | small nucleolar RNA, C/D box 90 |
| 17353131 | -4,000032064 | 0,000465162 | 0,132184757 | 0,583929509 | Ino80c | INO80 complex subunit C |
| **17258584** | **-4,012350585** | **0,000454365** | **0,132184757** | **0,560032735** | **Sphk1** | **sphingosine kinase 1** |
| 17481936 | -4,034499719 | 0,000439248 | 0,132184757 | 0,572627747 | Tead1 | TEA domain family member 1 |
| 17353241 | -4,039429942 | 0,000435793 | 0,132184757 | 0,626893294 | Slc25a46 | solute carrier family 25, member 46 |
| 17379128 | -4,051019678 | 0,000428882 | 0,132184757 | 0,619618662 | Srsf6 | serine/arginine-rich splicing factor 6 |
| 17365960 | -4,06333677 | 0,000422836 | 0,132184757 | 0,569277891 | Gfra1 | glial cell line derived neurotrophic factor family receptor alpha 1 |
| 17349774 | -4,067505514 | 0,000420244 | 0,132184757 | 0,746684615 | Hars2 | histidyl-tRNA synthetase 2, mitochondrial (putative) |
| 17239077 | -4,117804708 | 0,000387419 | 0,130631356 | 0,532540427 | Ginm1 | glycoprotein integral membrane 1 |
| 17219516 | -4,132601094 | 0,000380941 | 0,129771079 | 0,428596058 | Gm17224 | predicted gene 17224 |
| 17312905 | -4,142561209 | 0,000375326 | 0,129190212 | 0,583237458 | Eif3l | eukaryotic translation initiation factor 3, subunit L |
| 17533413 | -4,184770505 | 0,000349412 | 0,127526119 | 0,550405163 | Rpl3 | ribosomal protein L3 |
| 17275706 | -4,189147847 | 0,000345956 | 0,127526119 | 0,583912648 | Pnn | pinin |
| 17358020 | -4,205642406 | 0,00033991 | 0,127526119 | 0,576169778 | Nmrk1 | nicotinamide riboside kinase 1 |
| 17233799 | -4,207168462 | 0,000339478 | 0,127526119 | 0,5489078 | Slc25a16 | solute carrier family 25 (mitochondrial carrier, Graves disease autoantigen), member 16 |
| 17475818 | -4,231375384 | 0,000329544 | 0,127526119 | 0,454714086 | AF357399 | snoRNA AF357399 |
| 17469636 | -4,232978892 | 0,000328248 | 0,127526119 | 0,433734787 | Rad18 | RAD18 homolog (S. cerevisiae) |
| 17245399 | -4,28387533 | 0,000304062 | 0,12603958 | 0,562405539 | Irak3 | interleukin-1 receptor-associated kinase 3 |
| 17339549 | -4,337129628 | 0,000274692 | 0,124070176 | 0,583455003 | Ypel5 | yippee-like 5 (Drosophila) |
| 17277370 | -4,340198331 | 0,000273396 | 0,124070176 | 0,560278083 | Eif2b2 | eukaryotic translation initiation factor 2B, subunit 2 beta |
| 17402595 | -4,369250202 | 0,000264326 | 0,124070176 | 0,423236106 | Casp6 | caspase 6 |
| 17451203 | -4,369544715 | 0,000263894 | 0,124070176 | 0,667582272 | Srrd | SRR1 domain containing |
| 17522577 | -4,412134904 | 0,000250937 | 0,124070176 | 0,710931014 | Lrrfip2 | leucine rich repeat (in FLII) interacting protein 2 |
| 17345038 | -4,487677474 | 0,000224591 | 0,117799612 | 0,549881852 | Cd2ap | CD2-associated protein |
| 17515074 | -4,51117016 | 0,000211634 | 0,116707116 | 0,465606464 | Icam1 | intercellular adhesion molecule 1 |
| 17516217 | -4,518129424 | 0,000209042 | 0,116707116 | 0,331736836 | Olfr920 | olfactory receptor 920 |
| 17346749 | -4,537261637 | 0,000202996 | 0,116707116 | 0,601773665 | Rab31 | RAB31, member RAS oncogene family |
| 17265646 | -4,567629358 | 0,000194358 | 0,116707116 | 0,603423401 | Tekt1 | tektin 1 |
| 17286998 | -4,573030215 | 0,000192198 | 0,116707116 | 0,384288188 | Rbm24 | RNA binding motif protein 24 |
| 17283445 | -4,58655048 | 0,000184424 | 0,116707116 | 0,661719589 | Lgmn | legumain |
| 17224587 | -4,592282037 | 0,00018356 | 0,116707116 | 0,529509117 | Dnpep | aspartyl aminopeptidase |
| 17288716 | -4,857157317 | 0,000122661 | 0,096505067 | 0,417057385 | Glrx | glutaredoxin |
| 17263511 | -4,878684126 | 0,00011791 | 0,096074093 | 0,271489219 | Rasd1 | RAS, dexamethasone-induced 1 |
| 17347267 | -4,892003271 | 0,000115751 | 0,096074093 | 0,569578354 | Fez2 | fasciculation and elongation protein zeta 2 (zygin II) |
| 17364932 | -4,893219997 | 0,000114887 | 0,096074093 | 0,459150059 | Got1 | glutamic-oxaloacetic transaminase 1, soluble |
| 17508609 | -4,942716978 | 0,000106249 | 0,096074093 | 0,441015297 | Nrg1 | neuregulin 1 |
| 17483385 | -4,955833031 | 0,000104089 | 0,096074093 | 0,445527753 | Phkg2 | phosphorylase kinase, gamma 2 (testis) |
| 17486099 | -4,959257549 | 0,000103225 | 0,096074093 | 0,490802608 | Zim1 | zinc finger, imprinted 1 |
| 17219139 | -5,100936289 | 8,98364E-05 | 0,096074093 | 0,513086673 | Rgs5 | regulator of G-protein signaling 5 |
| 17497769 | -5,248765774 | 7,51516E-05 | 0,096074093 | 0,550423956 | Rnh1 | ribonuclease/angiogenin inhibitor 1 |
| 17519649 | -5,37953875 | 6,60816E-05 | 0,096074093 | 0,483671338 | Gsta4 | glutathione S-transferase, alpha 4 |
| 17424298 | -5,623221226 | 4,83734E-05 | 0,096074093 | 0,609742159 | Dctn3 | dynactin 3 |
| 17274184 | -6,947647979 | 1,33891E-05 | 0,088009903 | 0,33593012 | Socs2 | suppressor of cytokine signaling 2 |
