# Supplementary material for: Concomitant deletion of HRAS and NRAS leads to pulmonary immaturity, respiratory failure and neonatal death in mice
Source: Cell Death Dis. 2019 Nov 4;10(11):838. doi: 10.1038/s41419-019-2075-2 (PMC6828777; doi:10.1038/s41419-019-2075-2)
Supplement: Supplementary file 6 — Supplementary Table 4 [file 41419_2019_2075_MOESM6_ESM.docx]

**Table S4. Functional annotation of differentially expressed genes (downregulated and overexpressed) in the lungs of HRAS/NRAS-DKO mice treated antenatally with dexamethasone.**

The GeneCodis (Gene Annotation Co-occurrence Discovery) functional annotation tool (<http://genecodis.cnb.csic.es/> ) was used to identify statistically significant functional associations linking particular gene subsets contained within the list of differentially expressed gene probesets identified in the lungs of HRAS/NRAS-DKO mice that had been treated *in utero* with dexamethasone (injections at E17.5 and E18.5) in comparison to untreated DKO lungs [FDR=0.15; heatmap Fig 6B; Table S3 including 463 repressed (blue) and 46 overexpressed (red) genes] to specific cellular functionalities, including particular GO Biological Processes (BP) or Molecular Functions (MF), KEGG Signaling Pathways and Transcriptional Factors that may account for regulation of expression of the corresponding groups of loci listed in each case, as indicated. Red: overexpressed genes. Blue: repressed genes.

The columns labelled “*Functional Category*”, “*KEGG Pathway*” and “*Transcription Factor*” identify the specific functional GO (BP or MF), KEGG or TF terms recognized in each case for the corresponding groups of loci listed under the column labelled “*Genes*”. The column labelled “*Number of Genes*” indicates the specific number of genes annotated to the indicated functionality, out of the total number (in parenthesis) of genes recognized by GeneCodis in the lists of differentially expressed, overexpressed (red) or repressed (blue) genes identified in HRAS/NRAS-DKO lungs. The column labelled “*Hypergeometric pValue*” refers to the statistical significance assignated by Genecodis to each of the functional associations identified.

**GO Biological Process (BP) Enrichment Analysis of 463 Genes Downregulated/Repressed in the Lungs of HRAS/NRAS-DKO mice treated antenatally with dexamethasone**

| ***Item*** | ***Functional category*** | ***Number of Genes*** | ***Hypergeometric pValue*** | ***Genes*** |
| --- | --- | --- | --- | --- |
| GO:0016477 | cell migration (BP) | 8 | 9.76329e-05 | Gab2,Prkci,Snai2,Cd2ap,Shroom2,Nrg1,Srgap1,Vav2 |
| GO:0008152 | metabolic process (BP) | 35 | 1.5507e-03 | Mdm2,Agpat2,Gsta3,Naa20,Rpap2,Lgmn,Dad1,Ubr7,Eno3,Pappa,Pmm1,Pon2,Neu3,Aen,Nt5dc1,Gsta4,Ece1,Helb,Acpp,Ube2q1,Eci2,Casp6,Glce,Rhbdd2,Gstz1,Ids,Gsto1,Nudt9,Rnf167,Mocs1,Ppan,Myh6,Lsg1,Pafah1b2,Chst15 |
| GO:0006351 | transcription, DNA-dependent (BP) | 40 | 2.46154e-05 | Nkap,Nrbf2,Rbak,Sub1,Gtf2h5,Tead1,Polr2e,Taf1d,Gabpa,Ino80c,Tfdp2,Elf4,Lpin2,Nfrkb,Dmap1,Snai2,Sap30bp,Jmjd6,Med27,Arntl,Sarnp,Ikzf5,Xab2,Dnttip2,Zkscan14,Pnn,Chaf1b,Prim2,Tshz3,Preb,Polr3c,Gtf3c6,Junb,Tfb2m,Vdr,Gtf2ird1,Taf7,Uimc1,Znrd1,Senp2 |
| GO:0006351,  GO:0006281,  GO:0006974 | transcription, DNA-dependent (BP),DNA repair (BP),response to DNA damage stimulus (BP) | 6 | 1.80863e-05 | Gtf2h5,Ino80c,Nfrkb,Xab2,Chaf1b,Uimc1 |
| GO:0016310 | phosphorylation (BP) | 16 | 1.20854e-02 | Cdkn2b,Prkce,Cdkn1a,Stk39,Prkci,Irak3,Mapkapk5,Mapk13,Pak6,Rfk,Ripk3,Strap,Sphk1,Phkg2,Cdkn1c,Pip5k1b |
| GO:0016310,  GO:0007049,  GO:0007050,  GO:0042326 | phosphorylation (BP),cell cycle (BP),cell cycle arrest (BP),negative regulation of phosphorylation (BP) | 3 | 7.30561e-06 | Cdkn2b,Cdkn1a,Cdkn1c |
| GO:0006810  GO:0015031  GO:0034220  GO:0034220  GO:0016192  GO:0015992  GO:0015991 | transport (BP)  protein transport (BP)  transmembrane transport (BP)  ion transmembrane transport (BP)  vesicle mediated transport (BP)  proton transport (BP)  ATP hydrolysis coupled proton transport (BP) | 51 | 1.01063e-09 | Slc25a16,Slc25a3,Tmed5,Slc22a21,Kdelr3,Atp6v0a4,Tmem9,Kcnk1,Slc35b1,Slc38a4,Slc39a1,Slc7a8,Eif4enif1,Exoc5,Get4,Slc25a4,Slc25a18,Glrx,Svopl,Kcnj15,Atp6v0a2,Lyve1,Slc39a4,Stx12,Uqcrfs1,Rab7,Slc25a46,Cog2,Tomm22,Scnn1g,Tomm20,Snap23,Eif5a2,Arfgap3,Bet1l,Ndufs8,Slc35e1,Preb,Slc22a4,Vps33b,Slc25a12,Sfxn3,Rab5a,Gabarapl2,Slc17a5,Atp6v0c,Chmp2b,Lsg1,Blzf1,Thoc3,Senp2 |
| GO:0006915 | apoptotic process (BP) | 17 | 1.98053e-04 | Dad1,Sap30bp,Tax1bp1,Jmjd6,Tnfrsf10b,Irak3,Psen1,Aen,Bnip3,Ece1,Tctn3,Pdcd6,Casp6,Cecr2,Peg3,Srgn,Ddit4 |
| GO:0006915,  GO:0016485 | apoptotic process (BP),protein processing (BP) | 3 | 9.8589e-05 | Psen1,Ece1,Srgn |
| GO:0006281,  GO:0006974 | DNA repair (BP),response to DNA damage stimulus (BP) | 14 | 8.57345e-07 | Ube2b,Ercc8,Rnf168,Gtf2h5,Bccip,Ino80c,Atrip,Nfrkb,Rad18,Xab2,Nsmce2,Chaf1b,Alkbh3,Uimc1 |
| GO:0006974 | response to DNA damage stimulus (BP) | 17 | 3.41881e-07 | Ube2b,Ercc8,Rnf168,Gtf2h5,Cdkn1a,Bccip,Ino80c,Atrip,Nfrkb,Rad18,Psen1,Aen,Xab2,Nsmce2,Chaf1b,Alkbh3,Uimc1 |
| GO:0042254 | ribosome biogenesis (BP) | 9 | 4.30988e-08 | Nop56,Imp4,Eif6,Frg1,Imp3,Ebna1bp2,Rpf1,Gnl2,Sbds |
| GO:0006397,  GO:0008380 | mRNA processing (BP),RNA splicing (BP) | 15 | 1.62733e-08 | Cwc15,Usp39,Bcas2,Jmjd6,Snrnp40,Xab2,Pnn,Frg1,Ccdc55,Slu7,Snrnp27,Strap,Prpf18,Rnps1,Thoc3 |
| GO:0006412 | translation (BP) | 13 | 5.23382e-06 | Hars2,Mrpl39,Eef1b2,Mrpl4,Eif6,Mrpl30,Eif2s1,Eif2b2,Eif5a2,Mars,Rpl3,Eif3m,Eif3l |

**GO Molecular Function (MF) Enrichment Analysis of 463 Genes Downregulated/Repressed in the Lungs of HRAS/NRAS-DKO mice treated antenatally with dexamethasone**

| ***Item*** | ***Functional category*** | ***Number of Genes*** | ***Hypergeometric pValue*** | ***Genes*** |
| --- | --- | --- | --- | --- |
| GO:0005515 | protein binding (MF) | 85 | 3.3898e-13 | Thbs2,Ube2b,Dtnbp1,Mdm2,Rasd1,Kcnk1,Clec4e,Sumf1,Ppl,Cdkn1a,Tead1,Eif4enif1,Nfatc2ip,Anxa7,Tsen34,Slc25a4,Stk39,Cops5,Dnajb11,Atrip,Cdh13,Anxa1,Dmap1,Morf4l1,Sytl5,Dact1,Cd2ap,Kcnj15,Tax1bp1,Atp6v0a2,Ppp2r1a,Jmjd6,Rad18,Plcb4,Arntl,Psen1,Sarnp,Bnip3,Stx12,Uqcrfs1,Rab7,Shroom2,Tctn3,Poc5,Caml,Pdcd6,Rgs10,Casp6,Cops3,Cnst,Gabbr1,Tshz3,Avil,Ehd3,Polr1d,Prkrip1,Ppic,Ids,Strap,Fez2,Aim2,Golga5,Vps33b,Fzd5,Rab5a,Ier3,Junb,Gabarapl2,Atp6v0c,Emd,Myh6,Cdkn1c,Nufip1,Actn2,Vdr,Srgap1,Cep57,Srgn,Pip5k1b,Neat1,Vav2,Taf7,Blzf1,Rhoj,Eif3l |
| GO:0046872 | metal ion binding (MF) | 70 | 1.18608e-08 | Brpf1,Tpm4,Rbak,Rnf185,Mdm2,Rnf168,Dnpep,Prkce,Nubp2,Zfand2a,Sumf1,Cdkn1a,Zfp707,Iscu,Rpap2,Ptgs1,Anxa8,Prkci,Cops5,Sytl5,Aoc2,Snai2,Ubr7,Eno3,Asap2,Pappa,Usp39,Plcd1,Pmm1,Jmjd6,Pon2,Rad18,Narfl,Stambp,Itpa,Sharpin,Ikzf5,Uqcrfs1,Nt5dc1,Ece1,Nsmce2,Zkscan14,Ppm1d,Rfk,Zfp948,Bsn,Prim2,Zfp9,Zfyve21,Tshz3,Arfgap3,Zfp398,Slu7,Alkbh3,Ids,Ndufs8,Rnf167,Mocs1,Adap2,Rnf150,Zim1,Peg3,Zfp810,Nufip1,Pdzd8,Vdr,Rhou,Vav2,Znrd1,Nob1 |
| GO:0005525 | GTP binding (MF) | 17 | 1.39389e-06 | Rhobtb1,Rasd1,Rab7,Gpn2,Rasl11b,Tuba1b,Eif2b2,Ehd3,Rab31,Mocs1,Gnl2,Tubg2,Rab5a,Drg2,Lsg1,Rhou,Rhoj |
| GO:0000166 | nucleotide binding (MF) | 46 | 3.73507e-05 | Ube2b,Slc22a21,Rbm34,Rhobtb1,Rasd1,Prkce,Nubp2,Ube2j1,Hars2,Mrpl39,Ube2j2,Stk39,Prkci,Ppil4,Itpa,Irak3,Mapkapk5,Rab7,Mapk13,Abcc4,Helb,Pak6,Ube2q1,Gpn2,Rasl11b,Rfk,Tuba1b,Ripk3,Ehd3,Mars,Sphk1,Slc22a4,Rab31,Mocs1,Gnl2,Phkg2,Tubg2,Rab5a,Rnps1,Drg2,Myh6,Lsg1,Rhou,Pip5k1b,Rhoj,Mdh2 |
| GO:0005509 | calcium ion binding (MF) | 16 | 6.47956e-04 | Smoc2,Thbs2,Casq2,Fstl4,Anxa7,Ccbe1,Anxa8,Cdh13,Anxa1,Plcd1,Tbc1d8,Pdcd6,Ehd3,Man1c1,Slc25a12,Actn2 |
| GO:0016874 | ligase activity (MF) | 13 | 3.59128e-04 | Ube2b,Rnf185,Mdm2,Rnf168,Ube2j1,Hars2,Ube2j2,Ubr7,Rad18,Nsmce2,Ube2q1,Mars,Rnf167 |

**KEGG Pathways Enrichment analysis of 463 Genes Downregulated/Repressed in the Lungs of HRas/NRas-DKO mice treated antenatally with dexamethasone**

| ***Item*** | ***KEGG Pathways*** | ***Number of Genes*** | ***Hypergeometric pValue*** | ***Genes*** |
| --- | --- | --- | --- | --- |
| Kegg:04144 | Endocytosis | 12 | 1.47799e-05 | Rab31,Mdm2,Rab5a,Rab7,Pard6g,Chmp2b,Arfgap3,Pip5k1b,Stambp,Epn2,Ehd3,Prkci |
| Kegg:03013 | RNA transport | 10 | 2.52958e-05 | Senp2,Rnps1,Eif2b2,Sumo2,Eif2s1,Strap,Thoc3,Rae1,Pabpc1l,Pnn |
| Kegg:05152 | Tuberculosis | 10 | 6.09437e-05 | Mapk13,Atp6v0a2,Rab5a,Rab7,Clec4e,Atp6v0c,Sphk1,Atp6v0a4,Vdr,Ifngr1 |
| Kegg:04145 | Phagosome | 9 | 1.99499e-04 | Tuba1b,Atp6v0a2,Stx12,Rab5a,Rab7,Atp6v0c,Sftpd,Thbs2,Atp6v0a4 |
| Kegg:04142 | Lysosome | 7 | 8.04865e-04 | Ids,Sumf1,Atp6v0a2,Slc17a5,Atp6v0c,Atp6v0a4,Lgmn |
| Kegg:03040 | Spliceosome | 11 | 5.30376e-07 | Ccdc12,Snrnp27,Xab2,Slu7,Cwc15,Bcas2,Snrnp40,Usp39,Prpf18,Thoc3,Srsf6 |
| Kegg:00190 | Oxidative phosphorylation | 5 | 2.31671e-02 | Ndufs8,Atp6v0a2,Uqcrfs1,Atp6v0c,Atp6v0a4 |
| Kegg:03008 | Ribosome biogenesis in eukaryotes | 8 | 4.90372e-06 | Gnl2,Lsg1,Sbds,Imp3,Eif6,Nop56,Imp4,Nob1 |
| Kegg:00480 | Glutathione metabolism | 5 | 3.684e-04 | Gsta4,Gpx7,Gstz1,Gsto1,Gsta3 |
| Kegg:00510 | N-Glycan biosynthesis | 3 | 2.35913e-02 | Dad1,Alg13,Man1c1 |
| Kegg:00600 | Sphingolipid metabolism | 5 | 1.42658e-04 | Degs1,Neu3,Acer3,Acer2,Sphk1 |
| Kegg:04120 | Ubiquitin mediated proteolysis | 7 | 1.51542e-03 | Ube2j1,Rhobtb1,Mdm2,Ube2q1,Ube2j2,Ube2b,Ercc8 |
| Kegg:04141 | Protein processing in endoplasmic reticulum | 8 | 8.94547e-04 | Ube2j1,Dad1,Ufd1l,Preb,Dnajb11,Eif2s1,Man1c1,Ube2j2 |

**Functional annotation to “TRANSCRIPTION FACTORS” of 463 genes downregulated in the lungs of HRAS/NRAS-DKO mice treated antenatally with dexamethasone**

| ***Transcription Factor*** | ***Number of Genes*** | ***Hypergeometric pValue*** | ***Genes*** |
| --- | --- | --- | --- |
| V$FOXO4_01 | 39 | 3.47437e-06 | Dact1,Nubp2,Pdgfa,Pak6,Kcnj15,Plxna2,Agtr2,Anxa1,Glrx,Casq2,Nrg1,P2ry2,Usp54,Bsn,Rasd1,Brpf1,Gabarapl2,Tead1,Slc25a12,Gfra1,Cdkn1c,Nrbf2,Slc38a4,Eif2s1,Elf4,Cript,Pappa,Usp50,Tfdp2,Gab2,Dcn,Epn2,Vdr,Prkci,Ddit4,Ube2b,Sash1,Psen1,Ppm1d |
| V$MAZ_Q6 | 39 | 5.5142e-05 | Pak6,Iscu,Mocs1,Senp2,Rab31,Odf2,Znrd1,Nrg1,Usp54,Tfb2m,Gabarapl2,Myh6,Gadd45g,Gabbr1,Auts2,Junb,Polr1d,Slc25a12,Cdkn1a,Pard6g,Prkce,Gfra1,Phkg2,Tpbg,Slc7a8,Elf4,Pappa,Pi15,Fgfbp1,Tfdp2,Epn2,Got1,Prkci,Ube2b,Socs2,Pafah1b2,Fzd5,Cops3,Pnn |
| *V$TATA_01* | *28* | *3.22151e-05* | *Rasl11b,Pak6,Hist1h2bb,Plxna2,Peg3,Mia2,Anxa1,Rhobtb1,Farp1,Bnip3,Myh6,Gadd45g,Tead1,Aoc2,Junb,Crbn,Gfra1,Thbs2,Slc38a4,Man1c1,Pappa,Slc25a4,Icam1,Pi15,Rps19,Tfdp2,Actn2,Ube2b* |
| V$AP4_Q5 | 30 | 2.48305e-05 | Dact1,Pak6,Hist1h2bb,Ebna1bp2,Usp54,Bnip3,Abhd4,Krt19,Gadd45g,Mdm2,Anxa8,Polr1d,Sumo2,Loh12cr1,Srgap1,Nrbf2,Slc7a8,Eif2s1,Elf4,Man1c1,Spg21,Tfdp2,Gab2,Epn2,Cdh13,Ddit4,Ube2b,Eno3,Eif4enif1,Tubg2 |
| V$ELK1_02 | 30 | 2.40778e-08 | Mrps10,Agpat2,Odf2,Rpf1,Xab2,Ebna1bp2,Tfb2m,Ppan,Lrrfip2,Gabarapl2,Stx12,Rab5a,Pdcd6,Itpa,Ufd1l,Rnps1,Eif2b2,Asb8,Tax1bp1,Eif2s1,Blzf1,Timm23,Slc25a4,Spg21,Prpf18,Eef1b2,Tomm20,Tomm22,Pafah1b2,Cops3 |
| V$NFE2_01 | 17 | 1.59782e-08 | Ids,Pak6,Bnip3,Lrrfip2,Abhd4,Gadd45g,Mdm2,Fbxo44,Tead1,Cdkn1a,Slc7a8,Psmd11,Anxa7,Gab2,Vdr,Eno3,Plcd1 |
| V$AP1_Q6 | 13 | 6.37047e-07 | Ids,Pak6,Nfrkb,Lrrfip2,Abhd4,Cdkn1a,Pappa,Psmd11,Anxa7,Gab2,Dcn,Vdr,Plcd1 |
| V$ERR1_Q2 | 24 | 5.1437e-05 | Dact1,Iscu,Casq2,Nrg1,Bnip3,Gbas,Hmgb3,Aoc2,Tpm4,Atp6v0c,Cdkn1a,Gtf2ird1,Kcnk1,Elf4,Timm23,Slc25a4,Gabpa,Cdh13,Got1,Rin2,Eno3,Socs2,Slc25a3,Scnn1g |
| V$AP1_C | 32 | 1.39561e-08 | Ids,Rasl11b,Pak6,Nfrkb,Nrg1,Bnip3,Lrrfip2,Abhd4,Krt19,Gadd45g,Mdm2,Rab5a,Fbxo44,Arntl,Cdkn1a,Gfra1,Sftpd,Tpbg,Slc7a8,Slc35b1,Kcnk1,Pappa,Acpp,Pi15,Psmd11,Anxa7,Gab2,F3,Vdr,Rin2,Eno3,Plcd1 |
| V$SP1_Q6 | 53 | 2.92245e-07 | Nubp2,Pak6,Zfyve21,Senp2,Ier3,Hars2,Rbm24,Rab31,Peg3,Agpat2,Pon2,Odf2,Xab2,Tfb2m,Lrrfip2,Cops5,Brpf1,Gabarapl2,Krt19,Stx12,Rpl3,Tom1,Bet1l,Junb,Atp6v0c,Polr1d,Sumo2,Arntl,Loh12cr1,Fez2,Cdkn1a,Prkce,Gfra1,Tpbg,Slc7a8,Arfgap3,Llgl2,Blzf1,Pmm1,Rps19,Epn2,Prkci,Ube2b,Eno3,Socs2,Slc25a3,Plcd1,Psen1,Cd2ap,Ppm1d,Scnn1g,Fzd5,Ptges2 |
| *V$GABP_B* | *23* | *3.84835e-07* | *Ostf1,Iscu,Hars2,Ebna1bp2,Nufip1,Ppan,Arpc1b,Ufd1l,Rnps1,Eif2b2,Junb,Fez2,Nrbf2,Tax1bp1,Eif2s1,Elf4,Cript,Spg21,Gab2,Tomm22,Pafah1b2,Rae1,Cops3* |

**GO Biological Process (BP) and Molecular Function (MF) Enrichment Analysis of 46 Genes Overexpressed in Lungs of HRAS/NRAS-DKO mice treated antenatally with dexamethasone**

| ***Items*** | ***Functional Category*** | ***Number of genes*** | ***Hypergeometric pValue*** | ***Genes*** |
| --- | --- | --- | --- | --- |
| GO:0008152 | metabolic process (BP) | 6 | 1.52351e-02 | Mgam,Athl1,Atp9a,Ces1c,Ctss,Abcb11 |
| GO:0016787 | hydrolase activity (MF) | 8 | 4.20881e-04 | Try5,Mgam,Acap1,Athl1,Atp9a,Park7,Ces1c,Ctss |
| GO:0000166,  GO:0005525 | nucleotide binding (MF),GTP binding (MF) | 3 | 6.67704e-03 | Tufm,Arl8a,Gimap4 |

**KEGG Pathways Enrichment analysis of 46 Genes Overexpressed in Lungs of HRAS/NRAS-DKO mice treated antenatally with dexamethasone**

There are not annotations significantly enriched under any conditions

**Functional annotation to “TRANSCRIPTION FACTORS” of 46 genes Overexpressed in the lungs of dexamethasone*-*treated HRAS/NRAS-DKO mice**

| ***Transcription Factor*** | ***Number of genes*** | ***Hypergeometric pValue*** | ***Genes*** |
| --- | --- | --- | --- |
| V$ELK1_02, V$GABP_B | 3 | 3.87569e-03 | Elk3,Tufm,Tcof1 |
| V$GABP_B | 4 | 4.36912e-03 | Elk3,Tufm,Ctss,Tcof1 |
