# Supplementary figures and images for: Concomitant deletion of HRAS and NRAS leads to pulmonary immaturity, respiratory failure and neonatal death in mice
Source: Cell Death Dis. 2019 Nov 4;10(11):838. doi: 10.1038/s41419-019-2075-2 (PMC6828777; doi:10.1038/s41419-019-2075-2)

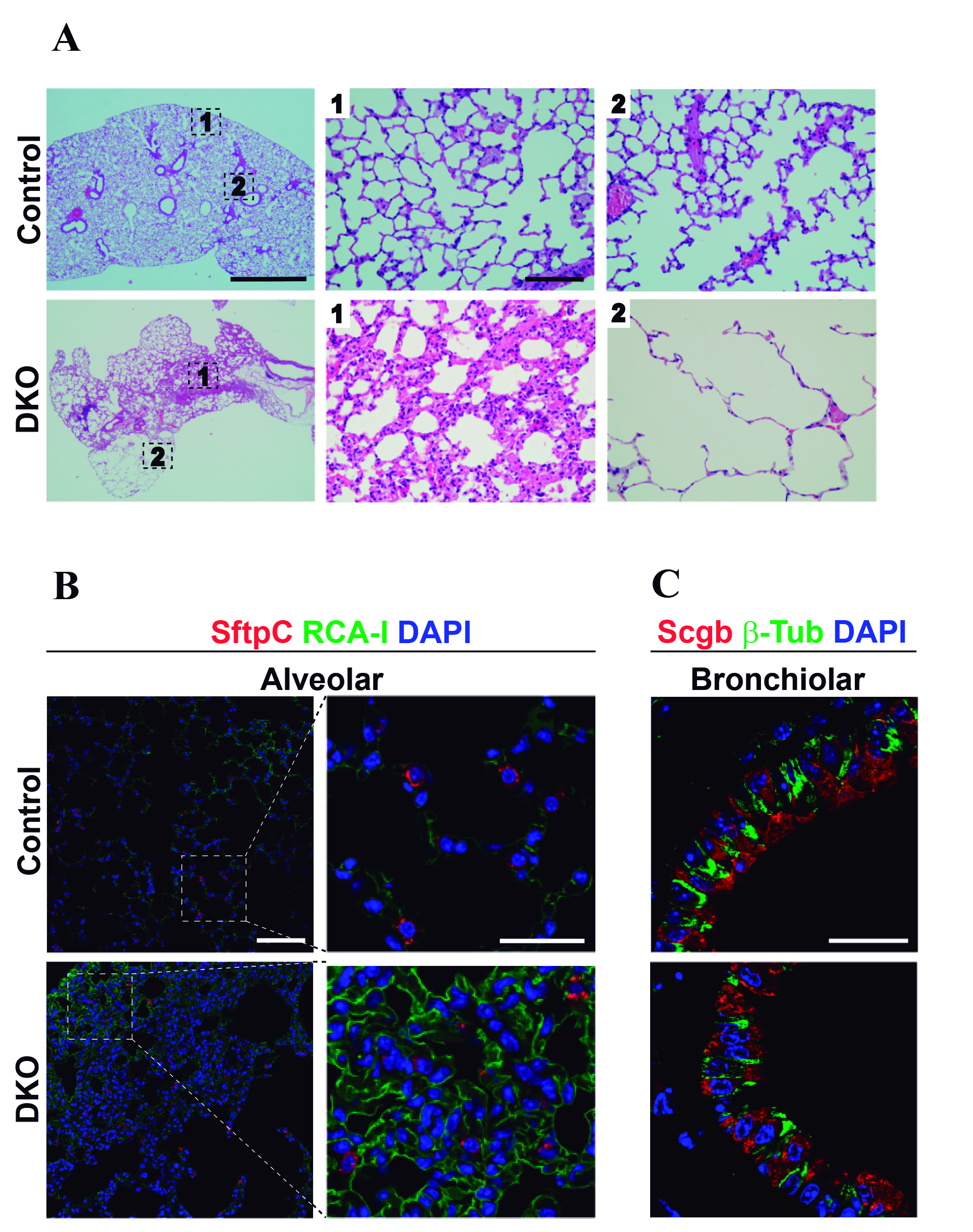

Supplement: Supplementary file 7 — Supplementary Figure 1 [file 41419_2019_2075_MOESM7_ESM.tif]

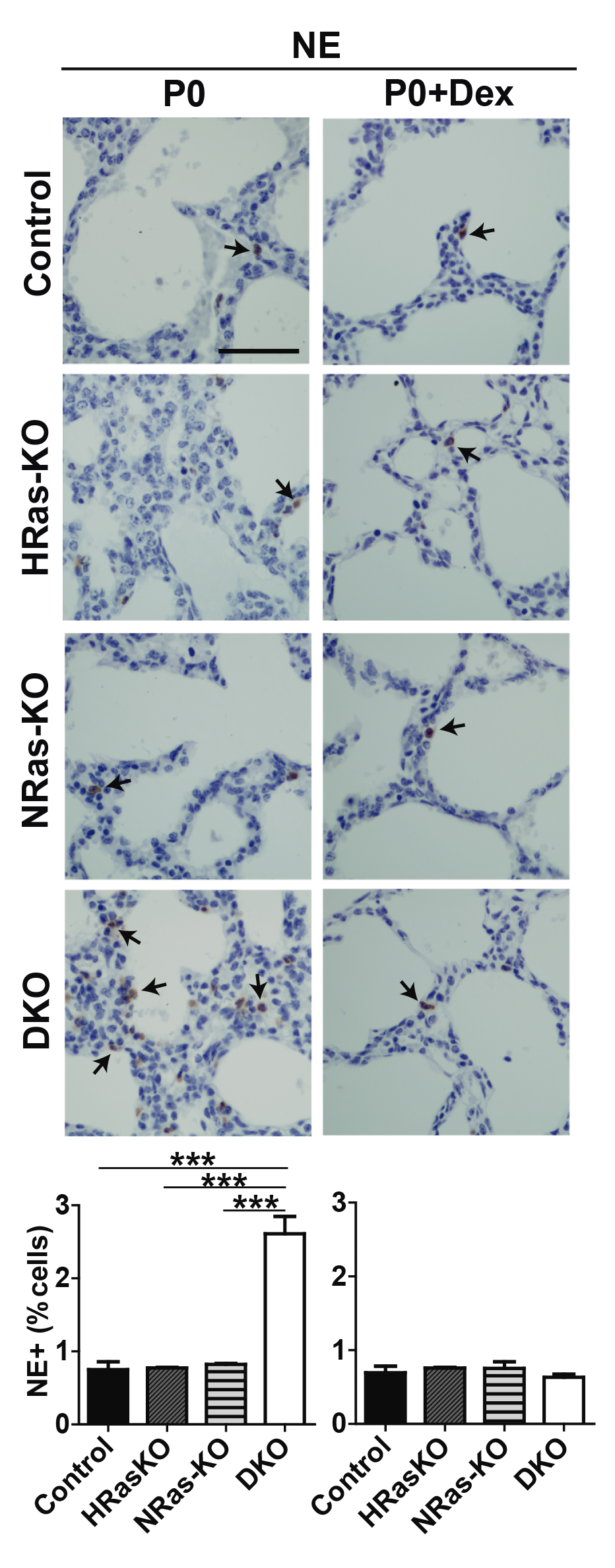

Supplement: Supplementary file 8 — Supplementary Figure 2 [file 41419_2019_2075_MOESM8_ESM.tif]

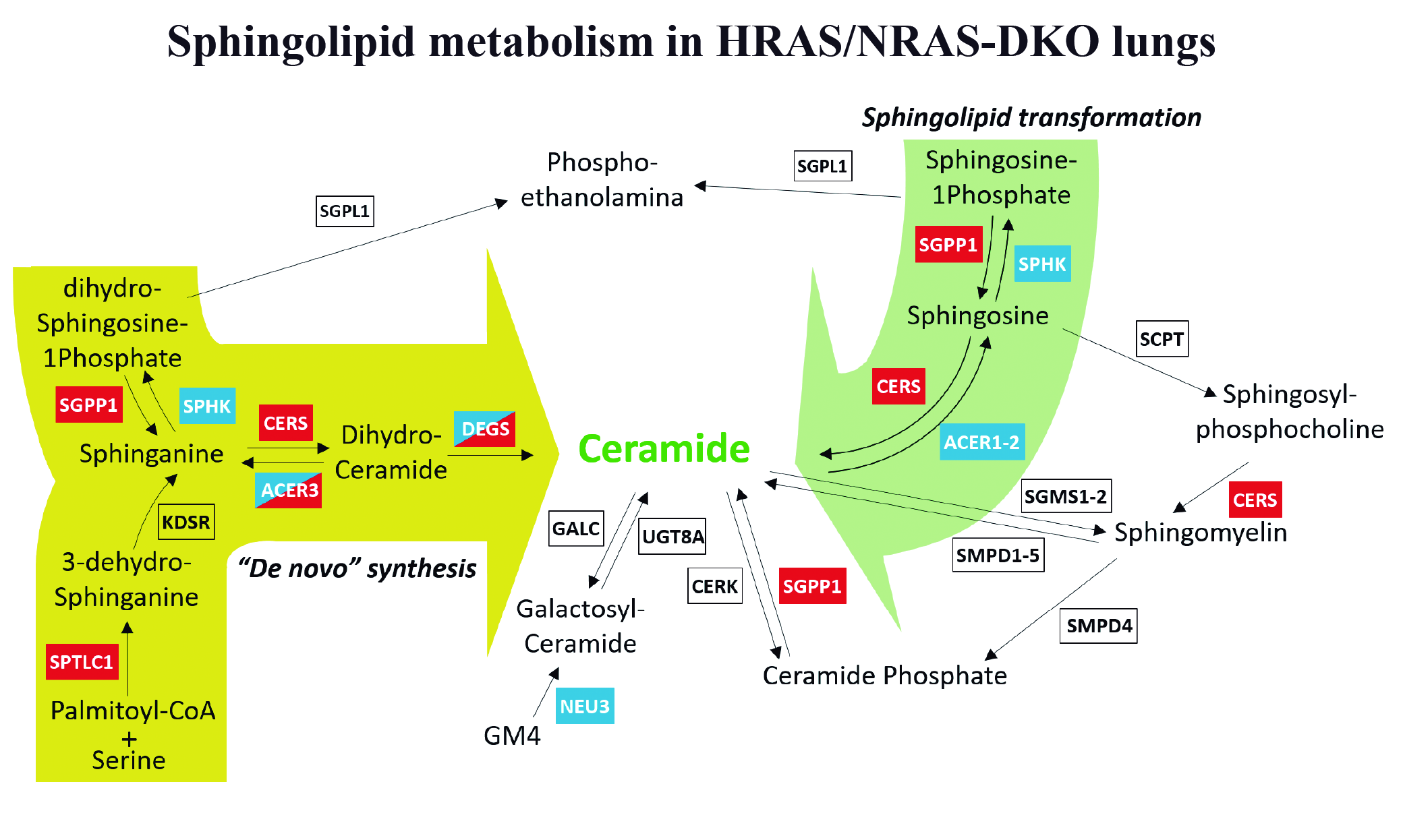

Supplement: Supplementary file 9 — Supplementary Figure 3 [file 41419_2019_2075_MOESM9_ESM.tif]
