# Supplementary material for: Concomitant deletion of HRAS and NRAS leads to pulmonary immaturity, respiratory failure and neonatal death in mice
Source: Cell Death Dis. 2019 Nov 4;10(11):838. doi: 10.1038/s41419-019-2075-2 (PMC6828777; doi:10.1038/s41419-019-2075-2)
Supplement: Supplementary file 10 — Author contribution [file 41419_2019_2075_MOESM10_ESM.pdf]

DECLARATION OF CONTRIBUTIONS TO ARTICLE

ADMC

Manuscript Number:

Journal Name: *Cell Death & Disease* (the 'Journal')

Proposed Title of the Contribution:

Concomitant deletion of HRAS and NRAS leads to pulmonary immaturity, (the 'Contribution)

Author(s):

Rocío Fuentes-Mateos, David Jimeno, Carmela Gómez, Nuria Calzada, Alberto (the 'Authors')

For all *CDD* articles, each person named as an author in the published version must be able to show he or she has contributed substantially to the article.

Authorship credit should be based on 1) substantial contributions to conception and design, acquisition of data, or analysis and interpretation of data; 2) drafting the article or revising it critically for important intellectual content; and 3) final approval of the version to be published. Authors should meet conditions 1, 2 and 3.

Any person who cannot be shown to have made a substantial contribution to the article cannot be listed as an author in the final version. The name of any person who is deemed to have made a minor contribution can, however, appear in the Acknowledgments section of the article.

Please complete the table below to indicate the contributions of all named authors to the manuscript.

Author Full Name:	Specification of Contribution to the Manuscript:
Rocio Fuentes-Mateos	Data acquisition and analysis. Figure preparation. Drafting
David Jimeno	Data acquisition
Carmela Gomez	Data acquisition
Nuria Calzada	Data acquisition
Alberto Fernandez-Medarde	Conception and design of the study. Data interpretation.Drafting
Eugenio Santos	Conception and design of the study. Data interpretation. Drafting

Please complete the table below to indicate the contributions of all named authors to the figures.

Figure 1:

Rocío Fuentes-Mateos (RFM): Preparation of figure, quantifications, data adquisition, Photographs, statistical analysis.
Nuria Calzada (NC): Genotyping.
Eugenio Santos (ES) & Alberto Fernández-Medarde (AFM): Figure corrections.

Figure 2:

Figure 2
RFM: Image adquisition, quantifications, statistical analysis.
Carmela Gomez (CG): PAS quantification (panels C, D). ES & AFM: Figure corrections.
Figure 3

Figure 3:

Figure 4
RFM: Staining, immunostaining, Image adquisition, quantifications, statistical analysis.
ES & AFM: Figure corrections.
Figure 5
RFM: Immunostaining, Image adquisition, quantifications, statistical analysis.

Figure 4:

Figure 6
RFM: Sample preparation, data analysis.
ES: Data analysis, figure design and corrections.

Figure 5:

Figure 7
RFM: Immunostaining, Image adquisition, quantifications, statistical analysis. ES & AFM: Figure corrections.

Figure 8
RFM: Staining, immunostaining, Image adquisition, quantifications, statistical analysis

Figure 6:

Supp. Fig 1:
RFM: Staining, immunostaining, Image adquisition, quantifications, statistical analysis. ES & AFM: Figure corrections.
Supp. Fig 2:
AFM: Figure preparation.
[illegible]

Signed for and on behalf of the Author(s):	Print Name:	Date:
	Eugenio Santos	16/09/2019
